# Intracellular Amplifiers of Reactive Oxygen Species Affecting Mitochondria as Radiosensitizers

**DOI:** 10.3390/cancers14010208

**Published:** 2021-12-31

**Authors:** Hong-Gui Xu, Viktor Reshetnikov, Marit Wondrak, Lisa Eckhardt, Leoni A. Kunz-Schughart, Christina Janko, Rainer Tietze, Christoph Alexiou, Hannes Borchardt, Achim Aigner, Wenjie Gong, Michael Schmitt, Leopold Sellner, Steffen Daum, Hülya Gizem Özkan, Andriy Mokhir

**Affiliations:** 1 Organic Chemistry Chair II, Department of Chemistry and Pharmacy, Friedrich-Alexander University Erlangen-Nürnberg (FAU), Nikolaus-Fiebiger-Str. 10, 91058 Erlangen, Germany; honggui.xu@fau.de (H.-G.X.); reshviktor1@gmail.com (V.R.); steffen.daum@gmail.com (S.D.); huelya.gizem.oezkan@fau.de (H.G.Ö.); 2 OncoRay—National Center for Radiation Research in Oncology, Faculty of Medicine and University Hospital Carl Gustav Carus, Technische Universität Dresden and Helmholtz-Zentrum Dresden—Rossendorf, 01307 Dresden, Germany; Marit.Wondrak@mailbox.tu-dresden.de (M.W.); Lisa.Eckhardt@uniklinikum-dresden.de (L.E.); leoni.kunz-schughart@oncoray.de (L.A.K.-S.); 3 National Center for Tumor Diseases (NCT), Partner Site Dresden, 01307 Dresden, Germany; 4 Department of Otorhinolaryngology, Head and Neck Surgery, Section of Experimental Oncology and Nanomedicine (SEON), Universitätsklinikum Erlangen, 91054 Erlangen, Germany; christina.janko@uk-erlangen.de (C.J.); Rainer.Tietze@uk-erlangen.de (R.T.); C.Alexiou@web.de (C.A.); 5 Rudolf-Boehm-Institute for Pharmacology and Toxicology, Clinical Pharmacology, Faculty of Medicine, Leipzig University, 04107 Leipzig, Germany; Hannes.Borchardt@medizin.uni-leipzig.de (H.B.); Achim.Aigner@medizin.uni-leipzig.de (A.A.); 6 Department of Medicine V, Heidelberg University Hospital, 69120 Heidelberg, Germany; wenjie.gong@med.uni-heidelberg.de (W.G.); michael.schmitt@med.uni-heidelberg.de (M.S.); lsellner.hd@gmail.com (L.S.); 7 Department of Hematology, First Affiliated Hospital of Soochow University, Suzhou 215006, China; 8 Takeda Pharmaceuticals, Cambridge, MA 02139, USA; 9 Merck, Im Laternenacker 5, 8200 Schaffhausen, Switzerland

**Keywords:** *N*-alkylaminoferrocene, cancer, mitochondrion, reactive oxygen species, prodrug, radiotherapy

## Abstract

**Simple Summary:**

Prodrugs that increase the level of reactive oxygen species (ROS) specifically in cancer cells while not affecting normal cells can potentially act as radiosensitizers for side effect free radiotherapy (RT). However, previously known ROS-amplifying prodrugs were found to not beuseful for this purpose. Since functional mitochondria are necessary for RT-resistance, we assumed that the problem could be solved by using dual prodrugs as radiosensitizers, both targeting mitochondria and simultaneously inducing ROS. In this paper, we explored this possibility. In particular, we developed an *N*-alkylaminoferrocene-based prodrug (2c) effective at low μMolar concentrations. Upon conversion to its active form 2c_BA in aqueous solution, it is efficiently taken up by cancer cells. This leads to the decrease of their mitochondrial membrane potential and the amplification of both, intracellular mitochondrial and total ROS generation. We found that 2c_BA acts as an efficient radiosensitizer in human head and neck squamous carcinoma cells in vitro.

**Abstract:**

Radiotherapy (RT) efficacy can be improved by using radiosensitizers, i.e., drugs enhancing the effect of ionizing radiation (IR). One of the side effects of RT includes damage of normal tissue in close proximity to the treated tumor. This problem can be solved by applying cancer specific radiosensitizers. *N*-Alkylaminoferrocene-based (NAAF) prodrugs produce reactive oxygen species (ROS) in cancer cells, but not in normal cells. Therefore, they can potentially act as cancer specific radiosensitizers. However, early NAAF prodrugs did not exhibit this property. Since functional mitochondria are important for RT resistance, we assumed that NAAF prodrugs affecting mitochondria in parallel with increasing intracellular ROS can potentially exhibit synergy with RT. We applied sequential Cu^+^-catalyzed alkyne-azide cycloadditions (CuAAC) to obtain a series of NAAF derivatives with the goal of improving anticancer efficacies over already existing compounds. One of the obtained prodrugs (2c) exhibited high anticancer activity with IC_50_ values in the range of 5–7.1 µM in human ovarian carcinoma, Burkitt’s lymphoma, pancreatic carcinoma and T-cell leukemia cells retained moderate water solubility and showed cancer specificity. 2c strongly affects mitochondria of cancer cells, leading to the amplification of mitochondrial and total ROS production and thus causing cell death via necrosis and apoptosis. We observed that 2c acts as a radiosensitizer in human head and neck squamous carcinoma cells. This is the first demonstration of a synergy between the radiotherapy and NAAF-based ROS amplifiers.

## 1. Introduction

**Figure 1:**
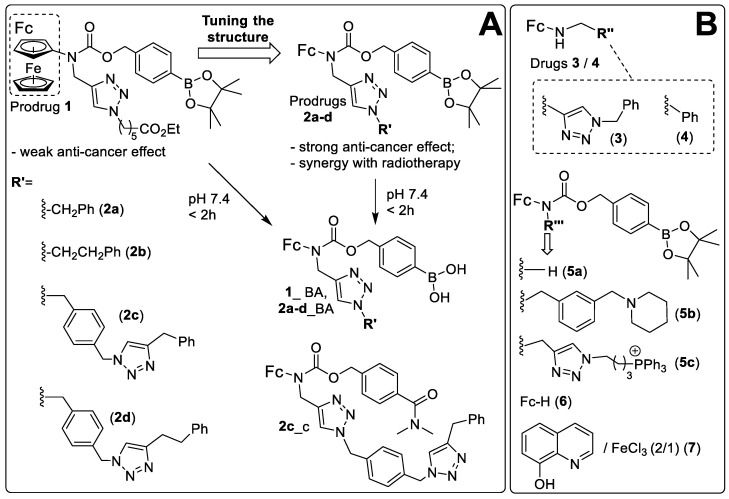
(**A**) Structures of the known prodrug 1 [21,24], its derivatives 2a-d and hydrolyzed at pH 7.4 forms of the prodrugs (1_BA, 2a_d_BA) as well as control 2c_c. (**B**) Structures of representative NAAF drugs 3 and 4 as well as reference compounds used in this work.

Radiotherapy (RT) using ionizing radiation (IR) is a common approach for the treatment of cancer [1,2]. It allows exclusively irradiating tumor areas, while sparing most of the body from the therapy-based toxicity. However, despite technological advances in 3D conformal radiation [3], the damage of the normal tissues located close to the tumor can still occur and may lead to side effects and the evolvement of RT-resistant cancer cells [4]. The former effect limits the power (dose) of the IR applicable in the treatment, whereas the latter decreases the success rate of repetitive therapy cycles. One possibility for the potentiation of RT includes its combination with radiosensitizers: chemotherapeutic drugs capable of enhancing anticancer effects of the IR, e.g., cisplatin, gemcitabine, 5-fluorouracil (5FU) or PARP inhibitors, e.g., veliparib [5,6,7,8]. The mechanism of their action relies either on the interaction with genomic DNA or the modulation of DNA metabolism. An alternative approach is based on the IR potentiation by drugs increasing intracellular levels of reactive oxygen species (ROS), e.g., sorafenib [9], l-S-buthionine (BSO) [10], As_2_O_3_ [11], diisopropylamine dichloroacetate (DADA) [12] and ferrocene derivatives [13,14]. However, despite their high antitumor efficacy, these drugs also affect healthy tissues and cells, which so far precludes potential clinical applications of RT approaches based on ROS-regulation. A promising strategy towards solving this problem could be the use of cancer-targeted prodrugs as radiosensitizers, which are activated only under tumor cell-specific conditions [15]. For example, cancer cells have the higher level of ROS, whereas the level of ROS in normal cells at homeostatic conditions is negligible [16,17,18]. Known ROS activated prodrugs include *N*-alkylaminoferrocene-based (NAAF)-prodrugs [18,19,20,21,22,23,24,25,26,27,28,29], pro-DNA alkylators [30,31], hydroxyferrocifens and their analogues [32,33], organochalcogen-based redox catalysts [34] and oligoferrocenes [35]. Unexpectedly, we have found that early NAAF prodrugs [18,19,21] exhibit no radiosensitizing effects (unpublished results) indicating that the intracellular ROS amplification alone is not sufficient to achieve the synergy with RT. Since functional mitochondria are crucially important for radioresistance [36], we hypothesized that NAAF prodrugs, which affect mitochondria of cancer cells additionally to the intracellular ROS amplification, could exhibit radiosensitizing properties. Though mitochondria in cancer cells are deficient and have abnormal membrane potential (MMP, ∆Ψ_m_ = 130–150 mV [37]), they still fulfill crucial for cancer cells functions including synthesis of nucleosides and ATP and play an important role in gene expression [38]. Therefore, many mitochondria targeting low molecular weight compounds [39,40] as well as nano-systems [41,42] are currently intensely explored as anticancer drugs. Unfortunately, no potent mitochondria-targeting NAAF prodrug was available for testing this hypothesis. In particular, although we have previously demonstrated that prodrug 1 (Figure 1) and its fluorogenic derivatives moderately affect mitochondria of cancer cells [25,28], their anticancer efficacy is very low [25].

One of the goals of this work was to obtain analogues of 1 with improved anticancer efficacy. This can be achieved by increasing lipophilicity of 1, ideally leading to enhanced passive uptake by cancer cells and increased anticancer efficacy [19,21,24]. However, compounds that are too lipophilic exhibit low solubility in aqueous buffers and are prone to formation of aggregates, which are not cell membrane permeable [24]. It should be possible to find a fine balance between increased lipophilicity and decreased water solubility by preparing focused libraries of prodrugs, where their properties are gradually altered in small steps. However, synthetic possibilities for chemical modification of NAAF prodrugs are restricted by oxidative instability of B-C bonds. For example, we have previously observed that single conjugations of carboxylic acid groups with amino groups in the presence of activating agents based on uronium salts or carbodiimides and Cu^+^-catalyzed alkyne-azide cycloadditions (CuAAC’s), conducted in aqueous buffers using Cu^2+^ salts as catalyst precursors, lead to complex product mixtures when conducted with the B-C bond containing derivatives. The problems become more severe when sequential reactions for the attachment of same or different chemical moieties are attempted. In particular, when the product yield in a single reaction is “y” (in %), the yield in the sequential reaction with N steps will be y^N^/100: e.g., for y = 60% and N = 2, y^N^/100 = 36%. Correspondingly, the number of side products will be higher in the sequential reactions. Sequential “click” reactions have not yet been used for synthesis of NAAF prodrugs. Recently, we have found conditions for a CuAAC reaction in non-aqueous buffers compatible with compounds containing B-C bonds. Using the single CuAAC, we have already prepared a series of conjugates of NAAF prodrugs with fluorescent dyes [22,25,28,29] and radio-tracers [27,43]. Here we applied both single (prodrugs 2a, 2b) and, for the first time, double sequential CuAAC reactions (prodrugs 2c, 2d) to generate a small library of derivatives of NAAF prodrug 1 (Figure 1). One of the compounds in this library (2c) turned out to be a substantially more potent mitochondria-targeted and ROS-amplifying prodrug than 1. On the example of 2c, we confirmed for the first time that NAAF prodrugs exhibit radiosensitizing properties in vitro. Furthermore, we obtained important new insights on the mechanism of action of mitochondria-targeted NAAF prodrugs.

## 2. Materials and Methods

### 2.1. General

Commercially available chemicals of the best quality from Sigma–Aldrich (Schnelldorf, Germany) were obtained and used without purification. Golgi-Staining-Green (BODIPY FL C5-ceramide complexed to BSA, catalog: B22650, Invitrogen™) and ER-Tracker-Green (BODIPY™ FL Glibenclamide, catalog: E34251, Invitrogen™) were obtained from ThermoFisher Scientific (Freiburg im Breisgau, Germany). NMR spectra were acquired on a Bruker Avance 300 or a Bruker Avance 400 spectrometer. Electrospray ionization (ESI) or atmospheric pressure photoionization (APPI) mass spectra were recorded on a Bruker ESI MicroTOF II focus or a Bruker maXis 4G. Elemental analysis (C, H and N) was performed in the microanalytical laboratory of the chemical institutes of the Friedrich Alexander University of Erlangen-Nürnberg, Erlangen, Germany. UV-visible spectra were measured on a Cary 100 UV-Vis Spectrophotometer (Agilent Technologies, Frankfurt am Main, Germany) by using quartz glass cuvettes (Hellma GmbH, Müllheim, Germany) with a sample volume of 1 mL. Fluorescence spectra were acquired on a Varian Cary Eclipse fluorescence spectrophotometer using fluorescence cuvettes (Hellma GmbH, Germany) with a sample volume of 1 mL. The fluorescence of live cells was quantified using a Guava easyCyteTM 6-2L flow cytometer from Merck Millipore (Darmstadt, Germany) or CytoFlex flow cytometer from Beckman Coulter, Inc (Fullerton, CA, USA). The data were processed using MS Excel. The microscopy images were taken with a Zeiss Axio Vert.A1 microscope. For dynamic light scattering measurements, a Zetasizer Nano series ZEN3600 (Malvern Instruments) with a 633 nm He-Ne laser was used with polystyrene cuvettes with a sample volume of 1000 µL (BRAND GmbH, Wertheim, Germany). The purity of the prodrugs used in the biological assays was determined by elemental analysis (C, H and N analysis). According to these data, the purity was greater than 95%.

### 2.2. Synthesis of Prodrugs and Their Characterization in Cell Free Settings

Previously known prodrugs 1 [23], 5a [18], 5b [22] and 5c [28] (main text, Figure 1) and intermediates S1 [24] and S2 [28] were synthesized as described elsewhere. The synthesis of prodrugs 2a–2d and their characterization (Figures S1–S42, SI (supporting information)) as well as protocols for the determination of n-octanol/water partition coefficients (logP), solubility of the prodrugs and prodrug-induced generation of ROS in cell free settings are described in the supporting information (SI).

### 2.3. Cells, Cell Culture and Cellular Assays

The human ovarian cancer cell line A2780 was purchased from Sigma–Aldrich. The Burkitt lymphoma cell line BL-2 and T-cell leukemia cell line Jurkat were obtained from DSMZ (Germany). SBLF9 fibroblasts were obtained from the group of PD Dr. B. Frey, Chair of Radiation Therapy, University Hospital Erlangen. The human pancreatic cancer cell line AsPC1 was obtained from the American Type Culture Collection (ATCC, Manassas, VA, USA). The human head and neck squamous cell carcinoma cell line SAS was obtained from the HSRRB (Japan), while FaDu cells represent a subline of FaDu-ATCC HTB-43 cells as described previously [44]. The above cell lines were cultivated following standard protocols from ECACC (93112519), DSMZ (ACC 625), ATCC (HTB-81) and Coriell. Suspension cells were grown to (0.5–1.5) × 10^6^ cells/mL and diluted as required. Adherent cells were cultivated to around 80% confluence. Effects of the prodrugs on the viability of cells, MMP, staining of cellular organelles (lysosomes, endoplasmic reticulum and Golgi), cell cycle, total and mitochondrial ROS, intracellular amount of glutathione and description of the study of synergistic effects of prodrug 2c and the ionizing irradiation are described in the SI.

## 3. Results and Discussion

### 3.1. Possible Reasons of Poor Anticancer Activity of Drug 1

To understand why prodrug 1 exhibits weak anticancer activity, we investigated its basic properties in cell free settings. First, we determined its n-octanol/water partition coefficient (logP) to evaluate the lipophilicity of 1 and its hydrolyzed form 1_BA. The latter compound is formed in aqueous neutral solution of 1 within <2 h. Analogously to other known NAAF-prodrugs [27], the hydrolyzed form is responsible for the biological activity of the prodrug. We found that 1 (logP = 4.87 ± 0.05) and especially 1_BA (logP = 2.83 ± 0.07) show moderate lipophilicity. We have previously observed that some lipophilic NAAF-prodrugs, e.g., 4-(*N*-ferrocenyl-*N*-benzylaminocarbonyloxymethyl)-phenylboronic acid pinacol ester [18,19,21,24], exhibit low reactivity towards ROS due to the formation of aggregates in aqueous solution, where the reactive arylboronic acid (BA) groups are not accessible to ROS. However, this was not the case for prodrug 1. For example, we observed that 1 reacts quickly with H_2_O_2_ in cell free settings, under formation of the corresponding NAAF, which catalyzes the generation of HO• from H_2_O_2_. The activity of 1 in the latter reaction was found to be identical to that of the positive control FeSO_4_ (Table 1). Thus, in cell free settings, 1 behaves as a very efficient NAAF-prodrug.

Previously, we determined that the efficacy of cellular uptake of prodrug 1 in A2780 cells is ca. 50% of that of the reference NAAF prodrug 4-(*N*-ferrocenyl-*N*-benzylaminocarbonyloxymethyl)-phenylboronic acid pinacol ester [25]. We confirmed the inefficient uptake of 1 by monitoring the increase of both boron and iron amounts in A2780 cells incubated with prodrug 1 by using atomic emission spectrometry (Table S1, SI). Thus, the low anticancer activity of 1 can at least partially be explained by the insufficient prodrug uptake.

### 3.2. Design, Synthesis and Characterization of the Prodrugs

We prepared a series of analogues of prodrug 1 with substituents of gradually increasing lipophilicity that was expected to improve the passive prodrug uptake. In particular, we replaced a long chain *N*-alkyl substituent in 1 (ClogP = 2.83) with substituents R’: benzyl (ClogP = 2.64, 2a), 2-phenylethyl (ClogP = 3.17, 2b), 4-((4-benzyl-1H-1,2,3-triazol-1-yl)methyl)benzyl (ClogP = 3.57, 2c) and 2-(4-((4-benzyl-1H-1,2,3-triazol-1-yl)methyl)phenyl)ethyl (ClogP = 3.95, 2d) (Figure 1). Specifically, 4-(*N*-ferrocenyl-*N*-propargylaminocarbonyloxymethyl)phenylboronic acid pinacol ester (compound S1, SI) was coupled to the corresponding organic azide derivatives under the conditions of the CuAAC reaction as described in detail in the SI. After chromatographic purification, all isolated products 2a–2d were of >95% purity according to the C, H, *N*-elemental analysis. We also prepared 2c_c, where the ROS-responsive moiety –B(OH)_2_ was replaced with a fragment –C(O)NMe_2_ (SI). This compound was used as a control, which does not react with ROS as a typical NAAF prodrug.

**Figure 2:**
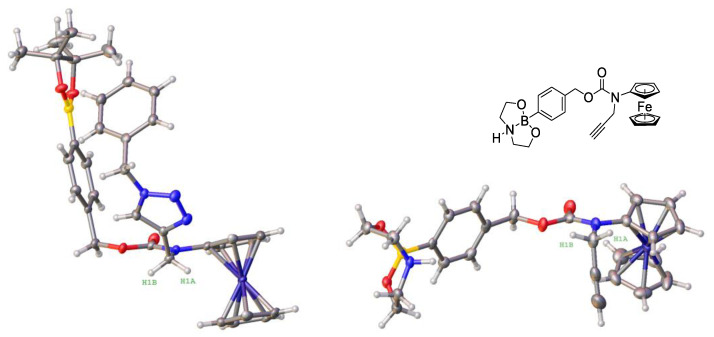
Comparison of the molecular structures of prodrug 2a (left structure) and previously published 4-(*N*-ferrocenyl-*N*-propargylaminocarbonyloxymethyl)phenylboronic acid *N*,*N*-diethanolamine ester (right structure [27]).

One of the prodrugs (2a) could be obtained in the crystalline form. We determined its crystal structure by X-ray crystallography (left structure, Figure 2), which confirmed the expected atom connectivity.

We found that hydrogen atoms H1a and H1b of the methylene group are directed towards the ferrocenyl moiety in the crystal structure of 2a. This is reflected in relatively short H1a--Fe (3.27 Å) and H1b--Fe (4.34 Å) distances, which, however, are close to or longer than the sum of van der Waals radii of hydrogen and iron atoms (3.06–3.39 Å [45]). Therefore, no strong intramolecular interactions can be assumed in this case and the realization of this conformation is probably governed by crystal packing effects. In contrast, in the previously reported structure of the related NAAF prodrug 4-(*N*-ferrocenyl-*N*-propargylamino-carnonyloxymethyl)phenylboronic acid *N*,*N*-diethanolamine ester (right structure, Figure 2) the methylene group is oriented away from the ferrocenyl moiety, leading to greater H1a--Fe (4.29 Å) and H1b--Fe (4.93 Å) distances [27].

### 3.3. Stability and Solubility of Prodrugs 2a–2d in the Aqueous Neutral Solution and Their Lipophilicity

To identify the active form of prodrugs 2a–2d in aqueous neutral buffers, we investigated their stability in the water rich solvent mixture containing triethylammonium acetate buffer (pH 7.0, 1.5 mM) and CH_3_CN (7/3, *v*/*v*). We observed that even freshly prepared solutions of analytically pure samples of 2a–2d (peaks eluted at 10.2–10.7 min) contain hydrolyzed species corresponding to boronic acid derivatives 2a_BA–2d_BA (peaks eluted at 7.1–7.5 min, 7–14% of the area of all peaks) (Figure S43, SI). After 2 h incubation of these samples, the peaks of 2a_BA–2d_BA become major ones (79–87% of the area of all peaks, Figure S43, SI). These data indicate that to analogously control 1 and the majority of other known NAAF-based prodrugs [27] the active forms of 2a–2d prodrugs are their boronic acid derivatives.

Next, we determined lipophilicity of the prodrugs and their BA-forms as described in the SI. We observed that lipophilicity is gradually increased in the series of the prepared prodrugs and their BA-derivatives, as compared to the parent prodrug 1 and its BA-derivative 1_BA, respectively (Table 1). This result was expected based on the CLogP values of the introduced substituents (see the previous section for the discussion on prodrug design principles).

To estimate solubility of the prodrugs, we diluted their stock solutions in dimethylsulfoxide (DMSO) 100-fold with Roswell Park Memorial Institute (RPMI) 1640 medium (Biochrom GmbH, Germany), supplemented with fetal bovine serum (FBS, 5%), l-glutamine (1%) and penicillin/streptomycin (1%, Biochrom GmbH, Germany). The latter medium was selected, since we used it for the cellular assays. The samples were monitored by bright field optical microscopy immediately and 48 h after the dilution. We observed first signs of precipitation at 50 µM for 2a and 2b and at 30 µM for 2c, 2d (Figure S44, SI). The prolonged incubation (48 h) of the prodrugs in the medium improved the solubility of 2a and 2b (first signs of precipitation observed at 100 µM), but not of 2c and 2d (Figure S44, SI). Then we monitored the formation of (not detectable in the previous experiment) aggregated species in prodrug solutions that appeared transparent (concentrations < 50 µM) by using dynamic light scattering. We found that prodrugs 2a and 2b form aggregates at concentrations above 30 µM, whereas in the case of prodrugs 2c and 2d, aggregate formation starts already at concentrations above 10 µM (Figure S45, SI).

### 3.4. Generation of ROS in the Presence of the Prodrugs in Cell Free Settings

As previously reported [18,19,20,21,22], the NAAF-prodrugs react with ROS like H_2_O_2_, first leading to the cleavage of the B-C-bond with the formation of the phenol derivatives (product 2c-1, Figure S49, SI). The latter compounds exist in equilibrium with the corresponding phenolates. The phenolates undergo the spontaneous 1,6-elimination of para-quinone methide (QM) followed by the release of CO_2_ with the formation of the *N*-alkylaminoferrocene NAAF drugs (product 2c-2, Figure S46, SI). By using electrospray ionization mass spectrometry (ESI–MS), we confirmed for one representative prodrug 2c that it reacts with H_2_O_2_ in cell free settings according to this common mechanism (ESI-MS, Figures S46–S48, SI). The NAAF is further oxidized by H_2_O_2_ or molecular oxygen with the generation of drug NAAF^+^ and highly toxic HO• or/and O_2_^•−^. Finally, the NAAF^+^ is reduced by glutathione (GSH), thereby closing the catalytic cycle, in which the NAAF acts as a catalyst. We evaluated the formation of HO• or/and O_2_^•−^ by using 2′,7′-dichlorodihydrofluorescein (DCFH) as a probe (Table 1, Figure S49, SI). This probe is oxidized to the fluorescent product 2′,7′-dichlorofluorescein (DCF) in the presence of HO• or/and O_2_^•−^. In particular, we found that NAAF drugs 3 (derived from the representative prodrug 2a) and 4 as well as prodrugs 2a and 2b exhibit similar catalytic efficacy in the conversion of H_2_O_2_ to HO• (Student’s *t*-test, Table 1). These data indicate that, under the conditions used in this assay, the prodrugs 2a and 2b are fully activated in the presence of ROS, with formation of the corresponding NAAF drugs. The ROS-generating activity of prodrugs 2c and 2d was found to be ~2-fold lower than that of 2a and 2b (*p* < 0.05, Student’s *t*-test). However, it still significantly exceeded that of ferrocene used as a negative control (*p* < 0.05, Student’s *t*-test). The lower activity of 2c and 2d correlates with their higher lipophilicity and their correspondingly higher propensity to form aggregates in aqueous solution (Figure S45, SI).

### 3.5. Anticancer Activity of the Prodrugs towards Different Cell Lines

We investigated the anticancer activity of all prepared prodrugs towards human ovarian cancer A2780 cells, which were selected as representative cell line. After a 96 h incubation time we found all prepared prodrugs to be more toxic in A2780 cells than the parent compound 1 (*p* < 0.01, Student’s *t*-test for all data pairs): IC_50_ values were determined to be in the range from 5.3 ± 1.9 (for 2c) to 12 ± 3 µM (for 2b) versus 30 ± 5 µM (for 1) (Table 2 and Table S2, SI).

This trend was also seen for 2a and 2b at shorter incubation times, whereas 2c and 2d did not reach IC_50_ under these conditions. The dose-response curves showing the dependence of cell viability on the prodrug concentration are given in Figure S50, SI. Similar results were obtained within prodrug pairs 2a/2b and 2c/2d, while differences between the pairs were found. In particular, increasing concentrations of prodrugs 2a and 2b caused a continuous reduction of the number of viable cells, whereas the effect of 2c and 2d was found to be saturated at 70%, 50% and 43% cell viability after 24, 48 and 96 h, respectively. This saturation can be explained by the tendency of the latter prodrugs to aggregate at >10 µM (Figure S45, SI), assuming that the aggregates are not active in contrast to the monomers. The enhanced anticancer effects of 2c and 2d at the longer incubation times may indicate anti-proliferative properties or time-dependent induction of cell death.

Among the prepared prodrugs, 2c was found to be most potent towards A2780 cells: IC_50_ = 5.3 ± 1.9 µM (96 h incubation). Therefore, it was selected for further biological studies. The anticancer efficacy of 2c towards A2780 cells is similar to that of the lysosome-targeting 5b [22] (IC_50_ = 7 ± 2 µM, Student’s *t*-test), which is the best currently known NAAF-prodrug in terms of anticancer efficacy in vitro and in vivo as well as cancer cell specificity. A previously reported conjugate 5c [24], consisting of an NAAF-prodrug covalently linked to a triphenylalkylphosphonium residue (TPP) and designed as a mitochondria-targeting prodrug, exhibited similar anticancer efficacy as well: IC_50_ = 5 ± 2 µM, Student’s *t*-test. However, 5c does not act as a typical NAAF prodrug according to the reported data [28]. In contrast, its anticancer efficacy could be interpreted as the direct toxicity of the TPP residue modulated by the NAAF moiety. The fact that cancer cell specificity of 5c was found to be limited precluded further in vivo studies on this compound. Therefore, throughout this paper, biological effects of 2c will be compared with 5b and the parent compound 1, rather than 5c.

We also evaluated the anticancer effects of 2c in other than A2780 cancer cells derived from different tumor moieties, including Burkitt’s lymphoma (BL-2 cells, Table S3, SI), pancreatic cancer (AsPC1 cells, Table S4, SI) and T-cell leukemia (Jurkat cells, Table S5, SI). We confirmed that all these cells contain the high level of ROS. Therefore, they were expected to be sensitive to ROS amplifying drugs [18,19,20,21,22,23,24,25,26,27,28,29]. Due to the slow kinetics of action of 2c observed in A2780 cells (Table S2, SI), incubation times of 48–96 h were selected. Prodrug 2c exhibited anticancer efficacy in all cell lines studied, exceeding that of the parent prodrug 1 (*p* < 0.05, Student’s *t*-test, Table 2 and Tables S3–S5, SI). These data indicate 2c as potentially applicable for the treatment of different tumor types.

One possible explanation of the higher activity of 2c as compared to 1 could be the stronger cellular uptake of 2c. According to logP values (Table 1), the energy independent or passive uptake of 2c was expected to be higher than that of 1. To confirm this estimation, we determined the uptake efficacy of 2c experimentally by monitoring both boron and iron level increase in A2780 cells incubated with 2c (5 µM). As controls, the cells incubated with a carrier only (DMSO, 1%, *v*/*v*) or with 1 (5 µM) were used (Table S1, SI). We observed that under the selected conditions the uptake efficacy of 2c is substantially higher than that of 1 (Student’s *t*-test, *p* < 0.001).

### 3.6. The Mechanism of the Anticancer Activity of Prodrug 2c

**Figure 3:**
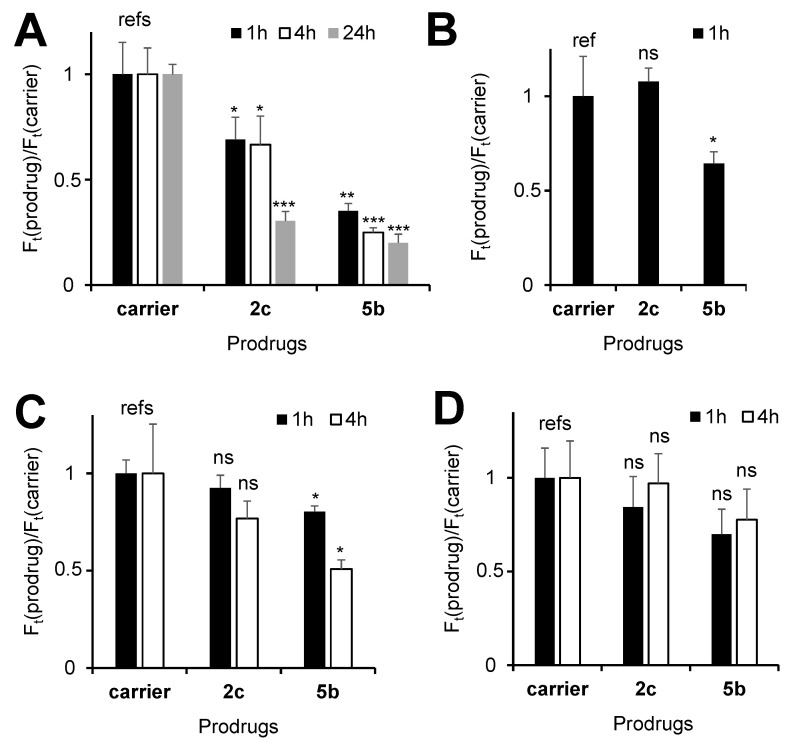
Effect of prodrugs 2c and 5b on relative organelle-specific staining (F_t_(prodrug)/F_t_(carrier)), with F: λ_ex_ = 488 nm, λ_em_ = 530 nm. (**A**) Mitochondria (Mit) staining with Rhodamine 123 (R123); t = 1, 4, 24 h. (**B**) Lysosomal staining with Acridine orange (AO); t = 1 h. (**C**) Endoplasmic reticulum (ER) staining with ER-Tracker-Green (ERG); *t* = 1, 4 h; (**D**) Golgi (G) staining with Golgi-Staining-Green (GO); t = 1, 4 h. *: *p* < 0.05; **: *p* < 0.01; ***: *p* < 0.001; ns: non-significant (Student’s *t*-test).

Encouraged by the favorable properties of 2c (high lipophilicity leading to the efficient intracellular uptake, sufficient solubility in aqueous buffers as well as high to moderate anticancer efficacy in different human cancer cells), we evaluated its mechanism of action. These experiments were conducted in two representative cells lines: Jurkat and A2780. We observed that 2c induces death of both Jurkat (Figure S51, SI) and A2780 cells (Figure S55, SI) mainly via necrosis, whereas apoptosis was found to play a minor role. In Jurkat cells, 2c caused the cell-cycle arrest in the G0/G1 phase (Figure S52, SI) analogously to the parent prodrug 1 [23] and the lysosome-targeting prodrug 5b [22]. However, while the prodrug 1 treatment led to a slight increase of both the MMP (Figure S53, SI) and intracellular GSH amounts (in both cases *p* < 0.05, Student’s *t*-test, Figure S54, SI), prodrug 2c behaved differently. In fact, it caused a decrease in the MMP (*p* < 0.01, Student’s *t*-test, Figure S53, SI) while leaving amounts of intracellular GSH unaffected in Jurkat cells (Figure S54). In A2780 cells, prodrug 2c reduced the MMP as well, dependent on the incubation time: values were determined at 69 ± 11 (1 h incubation) or 30 ± 5% (24 h incubation) compared to the MMP of untreated cells (Figure 3 and Figure S56, SI). However, no concentration dependency was found in the range of 5 to 30 µM, which is in agreement with the above-discussed formation of inactive aggregates at 2c concentrations >10 µM (Figure S45, SI). In contrast to Jurkat cells, the control 1 was able to reduce MMP in A2780 cells (Figure S56, SI). However, this decrease was again significantly weaker compared to prodrug 2c (e.g., at [prodrugs] = 5 µM and all tested incubation times, *p* < 0.05, Student’s *t*-test, Figure S56, SI).

**Figure 4:**
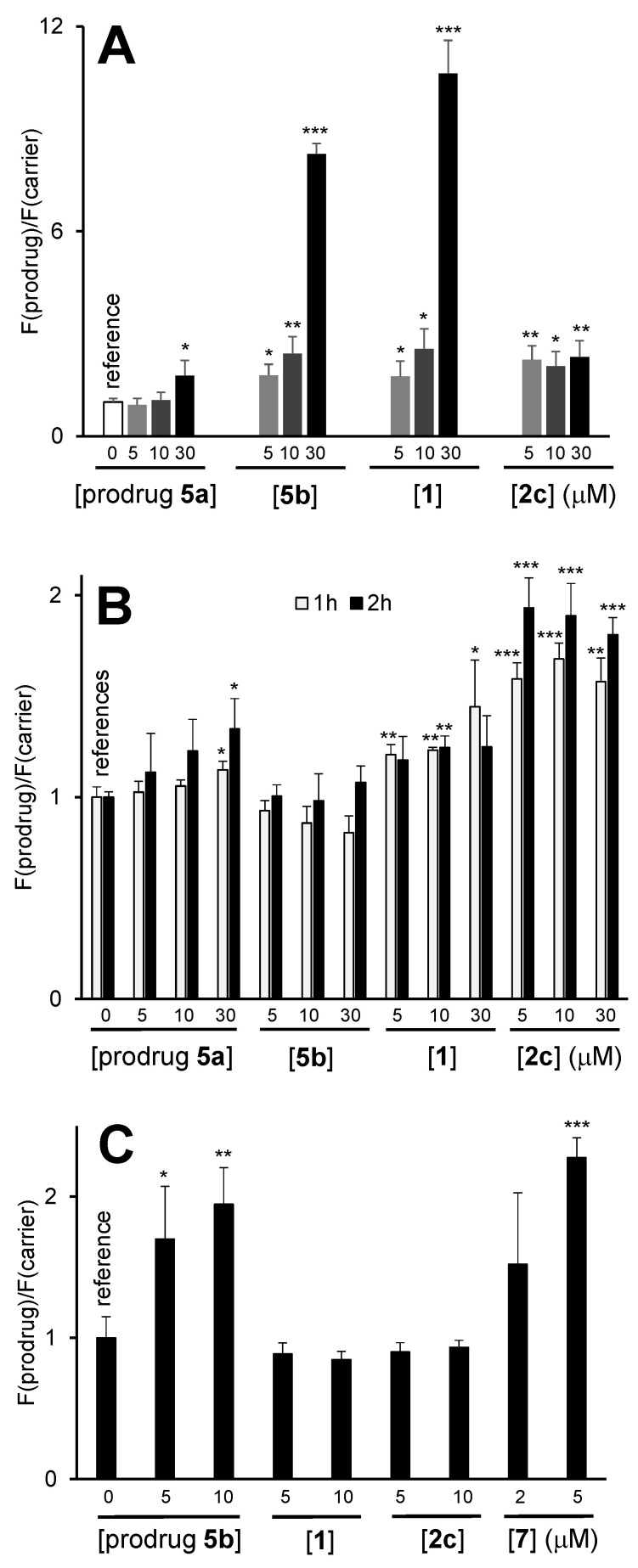
(**A**) Effects of prodrugs on the intracellular total ROS amount (tROS) in A2780 cells. F(prodrug)/F(carrier): the increase of the mean fluorescence (λ_ex_ = 488 nm, λ_em_ = 525 nm) of (CM-DCFH-DA)-loaded A2780 cells incubated with the prodrugs relative to that of the cells incubated with the carrier only (labelled “carrier”). The “carrier” sample was used as a reference. (**B**) Effects of prodrugs on the intracellular amount of mitochondrial ROS (mROS). F(prodrug)/F(carrier): the increase of the mean fluorescence (λ_ex_ = 488 nm, λ_em_ = 578 nm) of the A2780 cells incubated with the prodrugs, as determined using MitoSOX, relative to that of cells incubated with the carrier only (instead of the prodrugs). (**C**) Monitoring intracellular carbonyl derivatives in A2780 cells. F(prodrug)/F(carrier): the increase of the mean fluorescence (λ_ex_ = 488 nm, λ_em_ = 525 nm) in A2780 cells incubated with the prodrugs, as determined using DCCH, relative to that of the cells incubated with the carrier only (instead of the prodrugs). For (**A**–**C**). *: *p* < 0.05; **: *p* < 0.01; ***: *p* < 0.001 (Student’s *t*-test with respect to the “carrier” probe: indicated with “reference” on the plot).

Next, we evaluated effects of 2c on other than mitochondria organelles, including lysosomes (LY), endoplasmic reticulum (ER) and Golgi (GO). In these experiments, we stained the cells with established organelle-specific fluorescent dyes (Acridine orange: LY-specific; ER-Tracker-Green and Golgi-Staining-Green) and monitored the fluorescence changes in response to the treatment with prodrug 2c (Figure 3B–D). We found that the treatment of the cells with 2c affected neither LY nor ER nor GO. In contrast, the known LY-targeting control 5b strongly affected lysosomes already after a short incubation time of 1 h, as expected [22]. The latter prodrug also affected the MMP and the ER, which could be interpreted as a secondary effect caused by the initial 5b-induced disruption of lysosomes. These data thus confirm the high mitochondria-specificity of 2c. Since 2c is an NAAF prodrug, another expected effect would be the amplification of ROS in cells [18,19,20,21,22,23,24,25,26,27,28,29]. To evaluate whether this is the case, we used three different probes: the nonspecific probe 5-(6-)chloromethyl-2′,7′-dichlorodihydrofluorescein diacetate (CM-DCFH-DA) for the measurement of total ROS (tROS), the mitochondria-specific probe Red Mitochondrial Superoxide Indicator (MitoSOX) for the measurement of mitochondrial ROS (mROS) and 7-(diethylamino)coumarin-3-cerbohydrazide (DCCH) for monitoring ROS-induced formation of intracellular carbonyl-derivatives (Figure 4). We observed that 2c-induced tROS was significantly higher than its background levels in both A2780 (*p* < 0.01, Student’s *t*-test, Figure 4A) and Jurkat cells (*p* < 0.05, Student’s *t*-test, Figure S58, SI). At [prodrug] = 5 µM, the ROS increase in A2780 cells in the presence of 2c was similar to that induced by the parent prodrug 1. Since 2c exhibited substantially higher toxicity towards A2780 cells than 1 (5.3 ± 1.9 vs. 30 ± 5 µM, *p* < 0.05, Student’s *t*-test), these data indicated that the tROS is not the major determinant of 2c anticancer efficacy. Interestingly, we found mROS to be significantly increased in A2780 cells treated with 2c as compared to their non-treated counterparts (Figure 4B). In contrast, the LY-targeting prodrug 5b did not affect mROS, whereas the effects of the parent prodrugs 5a and 1 were found to be substantially weaker.

Despite producing mROS and tROS, prodrug 2c did not induce formation of significant amounts of carbonyl derivatives (Figure 4C). The analogous effect was observed for the parent prodrug 1, but was in contrast to that induced by the LY-targeting prodrug 5b. Based in these data, we conclude that the ROS-generating patterns of prodrugs 2c and 5b, including tROS, mROS and intracellular carbonyls, differ strongly from each other, thus indicating different modes of action of the prodrug 2c reported in this paper and the previously described prodrug 5b [22].

### 3.7. An Active Form of Prodrug 2c in Cells and the Role of Nitric Oxide in the Anticancer Activity of 2c

Despite the fact that 2c is activated in the presence of H_2_O_2_ as a typical NAAF prodrug (Figure S46, SI), in contrast to the control prodrug 1 as well as LY-targeting prodrug 5b, it does not exhibit high efficacy as a catalyst of ROS formation in cell free settings (Table 1). Furthermore, effects of 2c on intracellular ROS levels are also substantially different from those of NAAF prodrugs 1 and 5b as mentioned above. These differences might indicate that 2c from one side and 1 and 5b from another side act via different mechanisms.

**Figure 5:**
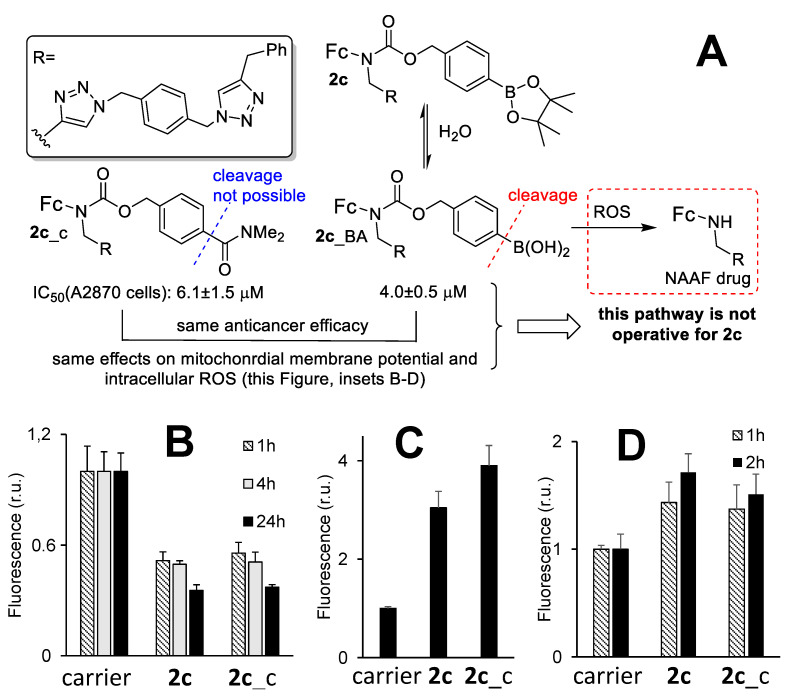
(**A**) An outline of the possible pathway of activation of 2c in cells: first, hydrolysis occurs with formation of 2c_BA, second, ROS-induced activation occurs with formation of the NAAF drug. ROS-resistant control 2c_c and 2c have identical cellular effects (**B**–**D**). Therefore, the ROS-induced 2c_BA activation is not operative for prodrug 2c. Effects of carrier (DMSO, 1%, *v*/*v*), 2c (5 µM) and 2c_c (5 µM) on (**B**) MMP (Fluorescence = F_t_(prodrug)/F_t_(carrier), where F: λ_ex_ = 488 nm, λ_em_ = 530 nm, r.u. = relative units, probe: R123, incubation time t = 1, 4, 24 h as indicated on the plot); (**C**) total ROS (tROS, same detection parameters as in B, probe: CM-DCFH-DA, t = 2 h) and (**D**) mitochondrial ROS (mROS, F: λ_ex_ = 488 nm, λ_em_ = 578 nm, probe: MitoSOX, t = 1, 2 h).

To gain some insights into the mode of action of 2c, we prepared a control compound 2c_c, in which a boronic acid residue was replaced with a resistant to ROS dimethylaminocarbonyl group (Figure 1 and Figure 5). The polarity of the latter group matches that of the B(OH)_2_ group, as it was confirmed by similar experimentally determined logP values of 2c_BA and 2c_c: 3.23 ± 0.11 and 3.90 ± 0.08, respectively (Table 1). Therefore, any major differences in cellular effects of these compounds were not expected to be due to differences in their uptake efficacy. If the activity of 2c relies exclusively on the cleavage of its B-C bond in the presence of ROS with formation of the cytotoxic NAAF drug (Figure 5A), the activity of the control 2c_c would be negligible.

We observed that not to be the case. Anticancer activity of 2c and 2c_c towards representative human cancer A2780 cells was observed to be the same: 4.0 ± 0.5 and 6.1 ± 1.5 µM, respectively (Student’s *t*-test: *p* = 0.0829, Table 2). Further, we investigated effects of 2c_c on MMP, mROS and tROS in A2780 cells and compared the data with those obtained for 2c (Figure 5B–D). The effects of 2c and 2c_c were found to be practically identical. Thus, these compounds exhibit not only similar anticancer activity, but also have the same mode of action. These data indicate that 2c is not activated in cells with formation of NAAF drugs, but rather acts as an intact drug 2c_BA (a hydrolyzed form of prodrug 2c, Figure 5A). The intracellular target molecule of 2c_BA is not known yet. However, it is apparent that this drug strongly affects mitochondria of cancer cells that can explain all other downstream effects: elevated mitochondrial and total ROS, cell death via apoptosis and necrosis.

It is known that nitric oxide (NO) strongly affects mitochondria by decreasing MMP and inducing ROS [46,47] that would be reminiscent with the effect of 2c. We found that the level of NO in A2780 cells treated with 2c is higher than that in A2780 cells treated with DMSO (carrier) only (*p* < 0.05, Student’s *t*-test, Figure S62, SI). The increased NO may provide a link between the increased ROS and decreased MMP observed in cancer cells in the presence of 2c.

### 3.8. Evaluation of Cancer Cell Specificity of the Prodrug 2c

**Figure 6:**
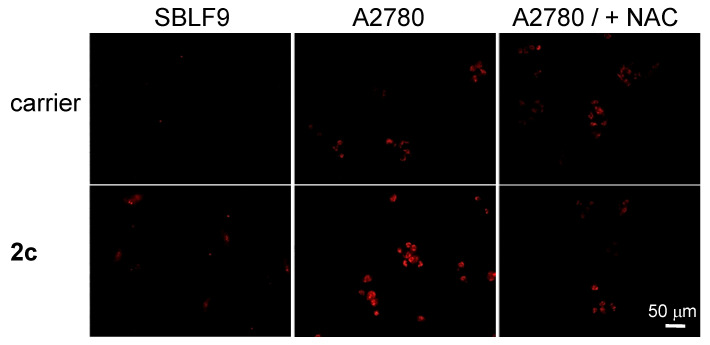
Effect of carrier (DMSO, 1%, *v*/*v*) and prodrug 2c (5 µM) (both incubated with the cells for 24 h) on mROS (stained using MitoSOX probes) in representative normal cells (SBLF9) and cancer cells (A2780). As indicated on the plot, in some cases *N*-acetylcysteine (NAC, 5 µM) was co-incubated with the carrier and the prodrug. Images obtained for the “carrier” and “2c” probes were compared qualitatively.

We evaluated the cancer cell specificity of 2c in two different experiments. In the first experiment, we monitored 2c—induced mROS generation in representative cancer (A2780) and normal cells (SBLF9 fibroblasts) (Figure 6). We found that 2c induces only a slight increase of mROS level in SBLF9 cells, whereas the effect is substantially stronger in A2780 cells. The mROS increase in A2780 cells can be fully eliminated by co-incubation of the prodrug with *N*-acetylcysteine (Figure 6).

**Figure 7:**
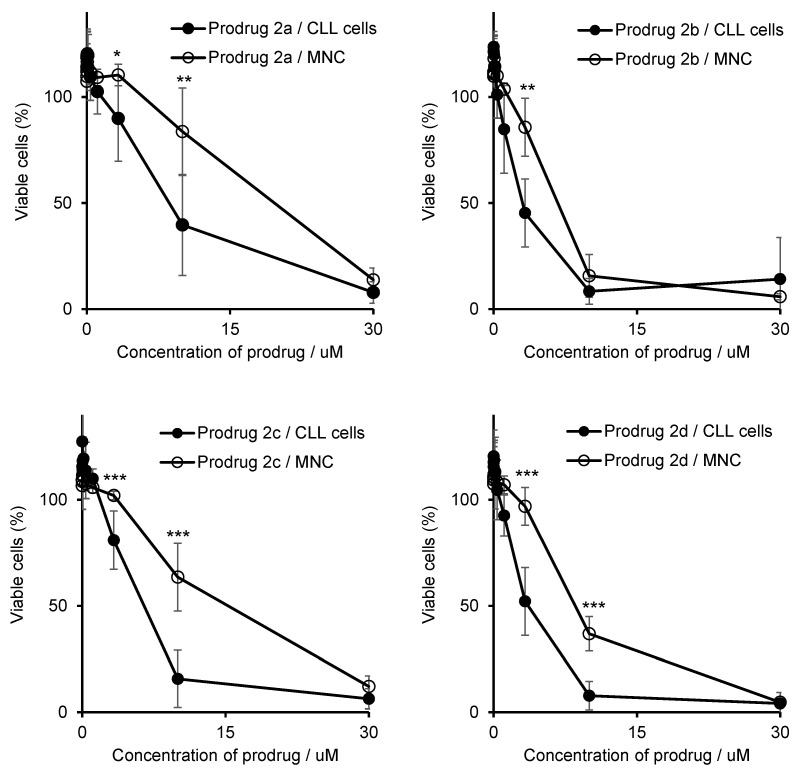
Effect of prodrugs 2a–2d on the viability of primary CLL cells and primary MNC’s. *: *p* < 0.05; **: *p* < 0.01; ***: *p* < 0.001 (Student’s *t*-test).

In the second experiment, cytotoxicity of the prodrugs towards chronic lymphocytic leukemia (CLL) cells isolated from six patients was determined and compared to that towards mononuclear cells (MNCs) from six healthy donors (Figure 7). The CLL cells are derived from B cells, whereas MNC are a mixture containing B cells, among others. Thus, these primary cancer and normal cells are genetically related to each other. Therefore, this is a good in vitro model for estimation of cancer cell specificity of new drugs. Notably, 2c was found to be significantly more toxic in the cancer cells than in the normal cells at concentrations of 3.3 and 10 µM (*p* < 0.001, Student’s *t*-test).

Concomitantly, IC_50_ of 2c were lower in CLL cells (6 ± 2 µM) compared to MNC cells (12 ± 3 µM, *p* < 0.01, Student’s *t*-test). Other prepared prodrugs (2a, 2b and 2d) exhibited some specificity towards primary CLL cells as well. However, in those cases the effect was not as profound as seen for 2c, thus confirming our selection of 2c as the best compound in the series of the prepared prodrugs.

### 3.9. Synergy of the NAAF-Prodrugs with Radiotherapy

Since prodrug 2c causes an MMP decrease (Figure 3A) and ROS increase (Figure 4) in cancer cells, we expected that it could exhibit anticancer effects synergistically with IR. To evaluate whether this is the case, we conducted experiments in two representative human head and neck squamous cell carcinoma (HNSCC) cell lines, SAS and FaDu. This selection was based on the following considerations: HNSCC is the 6th most common cancer and is usually treated by RT as well as chemotherapy (CT)/RT combination therapies, with a relatively unsatisfactory 5-year survival rate of ~50% for patients with advanced disease stage [48,49]. Therefore, the search for new radiosensitizers that are able to increase the responsiveness of HNSCC to RT is warranted.

The anticancer activity of the NAAF-based prodrugs in HNSCC has not been investigated before. Therefore, we first evaluated the effects of prodrug 2c, previously reported reference prodrug 5b as well as control compounds on SAS and FaDu cell viabilities. The effects were measured after relatively short incubation times of 24 and 48 h and compared to representative normal (retinal pigment epithelial ARPE-19, human fibroblast HF) cells (Tables S6 and S7, Figure S63, SI). We observed that the negative control 6 (ferrocene) and the slowly activated 2c did not reach measurable IC_50_ values under these conditions, whereas the positive non-specific control 7 exhibited high toxicity towards both, cancer and normal cells. Notably, the lysosome-targeting 5b was found to be more toxic in SAS cells than in both studied normal cells under the selected conditions. In contrast, FaDu cells turned out to be substantially more resistant to 5b (Table S6, SI). Despite the fact that 2c did not reach IC_50_ values according to the plots of cell viability versus prodrug concentration, the treatment of HNSCC cells with 2c led to a decrease of the number of viable cells in a concentration dependent manner (Figure S63, SI). Overall, these experiments confirmed that the NAAF-prodrugs 2c and reference prodrug 5b exhibit week to moderate anticancer activity towards HNSCC, thereby justifying further studies.

**Figure 8:**
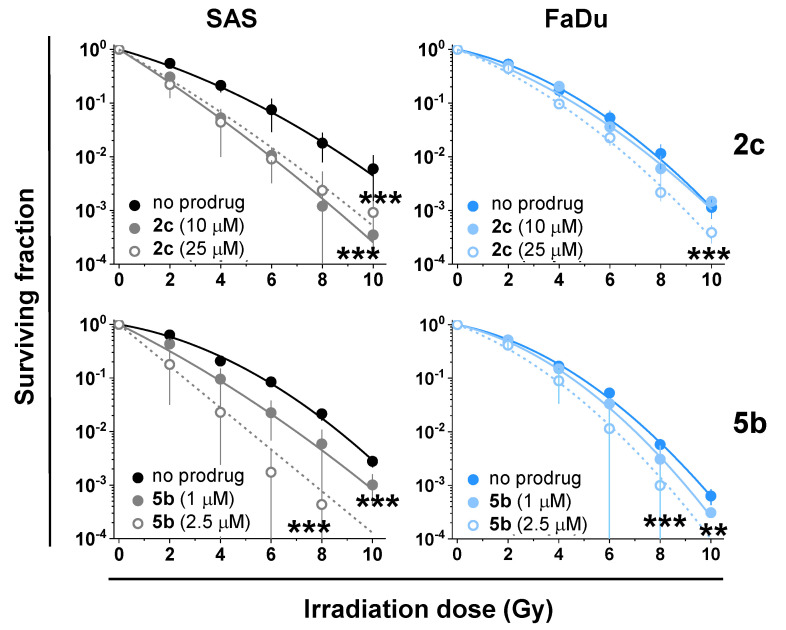
Representative data illustrating the synergy of prodrugs 2c and 5b with radiotherapy in SAS and FaDu cells. The graphs show clonogenic survival as a function of the irradiation dose. The data points represent mean values (±SD) from *n* = 3 independent experiments and the lines display the clonogenic survival curve fits. ** *p* < 0.01; *** *p* < 0.001.

For the evaluation of the synergy of IR with the NAAF-prodrugs, we compared effects of the individual effectors and their combinations on the colony formation capacity of SAS and FaDu cells (clonogenic assay). We selected the prodrug concentrations at around IC_80_-values, as determined in cell culture experiments (Table S8, SI). In both, cell culture-based and clonogenic assays, prodrug 2c was found to be less potent than 5b (Table S9, SI). For example, 25 µM 2c were able to reduce the growth of colonies of SAS and FaDu cells to 70 ± 10% and to 20 ± 8%, respectively, whereas a 10-fold lower dose of 5b was already sufficient for the reduction of colony formation of SAS cells (36 ± 15%) and of FaDu cells (36 ± 10%). Interestingly, FaDu cells were found to be more sensitive towards both tested NAAF-prodrugs in the clonogenic assay, which is in contrast to the trend observed in the experiment with cell cultures. Importantly, both NAAF-prodrugs 2c and 5b significantly potentiated the inhibitory effect of the IR on the ability SAS cells lines to form colonies (*p* < 0.001, Student’s *t*-test, Table 3, Figure 8).

The effect in FaDu cells was comparable (*p* < 0.001, Table 3), except that at the lowest tested concentration of 2c (10 µM) the prodrug did not affect the IR-induced inhibition of the colony formation. These data indicate that the NAAF-prodrugs exhibit the synergy with the IR under the optimized conditions. In contrast, ferrocene, which was used as a stable negative control, not reactive with ROS, did not act as a radiosensitizer at the selected conditions. Notably, the positive control 7 did not facilitate the IR effect in SAS cells either, but exhibited some synergy with the IR in FaDu cells (*p* < 0.01, Mann–Whitney test including Bonferroni–Holm correction).

## 4. Conclusions

We optimized the structure of the previously reported prodrug 1 by replacing the 5-ethoxycarbonylpenthyl residue with more hydrophobic rests R’. We thus obtained the prodrugs 2a-d (Figure 1) with the goal of fine-tuning the lipophilicity/cellular uptake efficacy without negatively affecting their solubility in aqueous buffers and cancer cell specificity. The best compound in this small series, 2c, exhibits significant anticancer efficacy in human ovarian carcinoma, human Burkitt’s lymphoma, human pancreatic carcinoma and T-cell leukemia cells, with IC_50_ values in the range of 5–7.1 µM. This is comparable to the lysosome-targeting prodrug 5b and the efficacies are substantially higher compared to 1. We observed that 2c causes the cell cycle arrest in G0/G1 phase and targets mitochondria, but not other organelles of cancer cells (lysosomes, endoplasmic reticulum, Golgi). This is accompanied by the increase of levels of both mitochondrial and total ROS as well as nitric oxide. However, it is not clear which of them is an upstream factor. In contrast to 2c, 5b does not induce an increase of mROS. Furthermore, it causes ROS-mediated formation of intracellular carbonyls, whereas 2c does not exert this effect. These data indicate that 2c and 5b act via different mechanisms and are, therefore, complementary to each other. This also suggests the possibility of synergistic effects between these prodrugs. Since 2c strongly affects mitochondria, mitochondria-driven apoptosis would be the obvious cell death mechanism for 2c. However, it does not seem to be the major route in this case. Necrosis is substantially more prominent. The latter can be caused by excessive ROS production leading to direct disturbance of cellular membrane.

Since mitochondria-targeting drugs often potentiate anticancer effects of radiotherapy, we investigated the radiosensitizing properties of 2c and the control prodrug 5b. The latter compound also affects mitochondria of cancer cells, which is a secondary effect caused by the 5b-induced disruption of lysosomes (Figure 3B). In both cases, we observed substantial synergistic effects with radiotherapy in human head and neck squamous cell carcinoma cell lines in the clonogenic assay. To the best of our knowledge, this is the first demonstration of the radiosensitizing effects of cancer specific ROS-amplifying prodrugs.

## Figures and Tables

**Table 1:** Selected properties of the prepared prodrugs and the control 1.

Prodrug	logP (Prodrug/Hydrolyzed Prodrug) ^i^	Solubility ^ii^	ROS Release Efficacy ^iv^
1	4.87 ± 0.05/2.83 ± 0.07	67 ± 5 ^iii^	0.99 ± 0.16
2a	5.27 ± 0.10/2.92 ± 0.14	100 (30)	0.74 ± 0.19
2b	5.37 ± 0.10/3.10 ± 0.12	100 (30)	0.76 ± 0.19
2c	5.37 ± 0.15/3.23 ± 0.11	30 (10)	0.35 ± 0.17
2c_c	3.90 ± 0.08	55 ± 5	0.050 ± 0.003
2d	5.56 ± 0.14/3.42 ± 0.14	30 (10)	0.37 ± 0.13

^i^ logP: n-octanol/water partition coefficient of the prodrugs and their hydrolyzed forms. Control 2c_c does not have a boronic acid residue, therefore, only one value is shown in the corresponding section of the table. ^ii^ Solubility of the prodrugs after their incubation for 48 h in Roswell Park Memorial Institute (RPMI) 1640 medium (Biochrom GmbH, Berlin, Germany) supplemented with fetal bovine serum (FBS, 5%), l-glutamine (1%) Penicillin/Streptomycin (1%, Biochrom GmbH, Germany) and containing dimethylsulfoxide (DMSO, 1%, *v*/*v*) (SI). The numbers in the brackets correspond to prodrug concentrations leading to aggregate formation (48 h incubation) determined by dynamic light scattering (SI). ^iii^ Solubility of prodrug 1 in Roswell Park Memorial Institute (RPMI) 1640 medium (Biochrom GmbH, Germany) supplemented with fetal bovine serum (FBS, 5%), l-glutamine (1%) Penicillin/Streptomycin (1%, Biochrom GmbH, Germany) and containing dimethylsulfoxide (DMSO, 1%, *v*/*v*) was determined as described previously [29]. ^iv^ ROS release efficacy, expressed as relative acceleration of the rate of fluorescence increase (λ_ex_ = 501 nm, λ_em_ = 523 nm) in the mixture of 2′,7′-dichlorodihydrofluorescein (DCFH) and H_2_O_2_ in aqueous solution buffered at pH 7.4. The ROS release efficacy for a background reaction (no prodrug added) is 0.04 ± 0.02, in the presence of ferrocene (negative control) −0.06 ± 0.02, in the presence of drug 3 (derived from prodrug 2a) −0.80 ± 0.19. The ROS release efficacy in the presence of FeSO_4_ was used as a reference: 1.0. Representative original data and detailed experimental conditions are provided in the SI.

**Table 2:** Anticancer effects (IC_50_) of prodrugs and controls towards different human cancer cell lines.

Prodrug/Control	IC_50_ (μM)/Human Cancer Cell Lines ^i^			
	A2780	BL-2	AsPC1	Jurkat
1	30 ± 5	17 ± 6	-	30 ± 4
2c	4.0 ± 0.5	5.7 ± 2.7	7.1 ± 1.0	5 ± 2
2c_c	6.1 ± 1.5	-	-	-
5b	7 ± 2	5 ± 3	6.7 ± 0.5	7.2 ± 0.1
6	>100	>100	-	>100

^i^ IC_50_: a drug concentration, at which 50% viable cells remain in the mixture. A2780: human ovarian cancer cell line (incubation time 96 h); BL-2: human Burkitt’s lymphoma cell line (incubation time 96 h); AsPC1: human pancreatic cancer cell line (incubation time 72 h); Jurkat: T-cell leukemia cell line (incubation time 48 h).

**Table 3:** Statistical comparison (*p*-values) of irradiation dose–response curves with and without sub-toxic concentrations of the drug candidates.

Prodrug	Concentration (µM)	Radiosensitization ^i^	*p*-Values ^ii^	
			SAS	FaDu
2c	10	+/−	<0.001	ns
	25	+	<0.001	<0.001
5b	1	+	<0.001	<0.01
	2.5	+	<0.001	<0.001
6	25	-	ns	ns
7	0.1	+/−	ns	<0.01

^i^ The radiosensitizing effect was evaluated semi-quantitatively as no radiosensitization (“-”), weak effect or effect in one cell line only (“+/−”), or clear/strong (“+”) radiosensitization. ^ii^ *p*-Values were determined by Mann–Whitney tests including Bonferroni–Holm correction using IBM SPSS Statistics 21; ns = not statistically significant (*p* > 0.05).

## Data Availability

All data are available by direct requests to the corresponding author.

## Supplementary Materials

The following are available online at https://www.mdpi.com/article/10.3390/cancers14010208/s1, Figures S1–S42: ^1^H-NMR, ^13^C-NMR and high resolution mass spectra (HRMS) of reported intermediates, prodrugs and drugs, Figure S43: HPLC profiles of prodrugs 2a–2d (30 µM) and the control probe incubated in CH_3_CN/triethylammonium acetate buffer—pH 7.0, 1.5 mM, 3/7, *v*/*v* for 0 and 2 h, Figure S44: Microscopic images (Objective 40×/0.5 Ph0, Eyepiece WF 10×/20 Br. foc.) of solutions/suspensions of prodrugs 2a–2d in RPMI 1640 medium obtained either immediately (labelled “0 h”) or 48 h after mixing all components (labelled “48 h”), Figure S45: Correlation of mean particle size (Z-average diameter) and concentration of prodrugs compounds in RPMI 1640 medium, Figures S46–S48: Structural formulas and exact masses of products identified in the mixtures of 2c with/without H_2_O_2_ by using high resolution ESI-TOF mass spectrometry, Figure S49: Increase of the fluorescence intensity (λ_ex_ = 501 nm, λ_em_ = 523 nm) upon oxidation of DCFH by H_2_O_2_ in the presence of FeSO_4_, Figure S50: Effects of prodrugs 2a–2d at different incubation times on viability of A2780 cells as determined by using the MTT assay described above, Figure S51: The mechanism of Jurkat cell death in the presence of prodrugs 1 and 2c (incubation time: 48 h), Figure S52: Effect of prodrugs 1 and 2c (48 h incubation) on Jurkat cell cycle, Figure S53: MMP of Jurkat cells after their incubation for 48 h with prodrugs 1 and 2c, Figure S54: Effect of prodrugs 1 and 2c (48 h incubation) on the intracellular amount of GSH in Jurkat cells, Figure S55: Effects of the prodrugs on the viability of A2780 cell lines after 48 h incubation, Figure S56: Effects of the prodrugs on MMP of A2780 cell lines after 1–48 h incubation, Figure S57: Representative flow cytometric histograms used for setting up Figure 3A–D (main text) and Figure 5A. Figure S58: Effects of prodrugs 2c and 5b on the intracellular total ROS amount (tROS) in Jurkat cells. Figure S59. Representative flow cytometric histograms used for setting up Figure 4A and Figure 5C. Figure S60. Representative flow cytometric histograms used for setting up Figure 4B and Figure 5D. Figure S61. Representative flow cytometric histograms used for setting up Figure 4C. Figure S62. Fluorescence of DAF-FM diacetate-loaded A2780 cells treated either with 2c or the carrier. *: *p* < 0.05, Student’s *t* test. Figure S63: Representative experimental data showing relative survival as function of drug concentration in two HNSCC cell lines and two normal cell lines as determined via the MTT assays. Table S1: Relative boron amount in A2780 cells treated with carrier, prodrugs 1 or 2c, Table S2: Effects of the prodrugs on the viability of A2780 cell lines (IC_50_ values) after different incubation times, Table S3: Effects of the prodrugs on the viability of BL2 cell line (IC_50_ values) after 48 and 96 h incubation, Table S4: Effects of the prodrugs on the viability of AsPC1 cell lines (IC_50_ values) after 72 h incubation, Table S5: Effects of the prodrugs on the viability of Jurkat cell lines (IC_50_ values) after 48 h incubation, Table S6: EC_50_ values (50% survival) in cancer SAS and FaDu cells determined via the MTTassay after 48 h of exposure to the prodrugs, Table S7: EC_50_ values (50% survival) in representative normal ARPE-19 and human fibroblasts (HF) cells determined via the MTT assay after 48 h of exposure to the prodrugs, Table S8: EC_20_ values (80% survival) in cancer SAS and FaDu cells determined via the MTT assay after 24 h and 48 h of exposure to the prodrugs, Table S9: Effects of the prodrugs on the capacity of SAS and FaDu cells to form colonies.
