# Supplementary material for: Intracellular Amplifiers of Reactive Oxygen Species Affecting Mitochondria as Radiosensitizers
Source: Cancers (Basel). 2021 Dec 31;14(1):208. doi: 10.3390/cancers14010208 (PMC8750417; doi:10.3390/cancers14010208)
Supplement: Supplementary file 1 [file cancers-14-00208-s001.zip › cancers-1534116-supplementary.pdf]

SUPPORTING INFORMATION

Intracellular amplifiers of reactive oxygen species affecting mitochondria as radiosensitizers

Hong-Gui Xu, Viktor Reshetnikov, Marit Wondrak, Lisa Eckhardt, Leoni A. Kunz-Schughart, Christina Janko, Rainer Tietze, Christoph Alexiou, Hannes Borchardt, Achim Aigner, Wenjie Gong, Michael Schmitt, Leopold Sellner, Steffen Daum, Hülya Gizem Özkan, Andriy Mokhir*

Content

1. Synthesis	S2-S29
2. Determination of crystal structure of prodrug **2a**	S29
3. Evaluation of hydrolysis of prodrugs in the aqueous neutral solution by using HPLC-UV-MS	S29-S30
4. Determination of n-octanol/water partition coefficients (LogP)	S31
5. Solubility of drugs in aqueous buffers	S31-S33
6. Activation of prodrug **2c** in the presence of H_2O_2	S33-S37
7. Prodrug-induced generation of ROS in cell free settings	S38-S39
8. Accumulation of prodrugs in A2780 cells	S39
9. Determination of the viability of cells in the presence of prodrugs and controls	S40-S48
10. Effect of prodrugs and controls on cellular organelles in A2780 cells: lysosomes (LY), endoplasmic reticulum (ER), Golgi (G) and mitochondria (Mit)	S48-S50
11. Monitoring total intracellular ROS (tROS)	S51-S52
12. Monitoring mitochondrial ROS (mROS)	S52-S53
13. Monitoring intracellular carbonyl derivatives	S53-S54
14. Generation of nitric oxide in A2780 cells	S54-S55
15. Effect of prodrug **2c** on mitochondrial reactive oxygen species in representative cancer (A2780) and normal cells (SBLF9 fibroblasts).	S55-S56
16. Determination of viability of primary cancer (CLL) and normal (MNC) cells in the presence of prodrugs	S56
17. Study of synergy of prodrugs and controls with radiotherapy for SAS and FaDu cells	S57-S61
18. References	S61

Synthesis

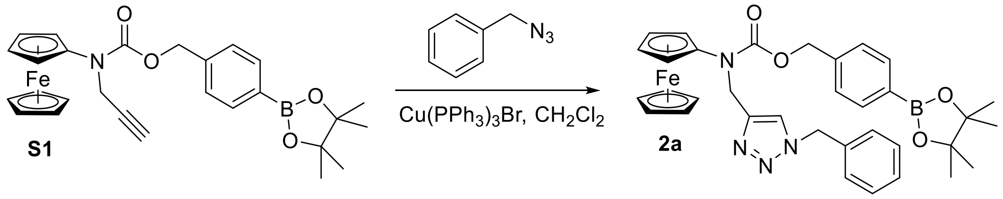

Prodrug **2a** Compound **S1** (100.0 mg, 200 µmol) was dissolved in anhydrous dichloromethane (4 mL). Benzyl azide (53.3 mg, 50 µL, 400 µmol) and bromotris-(triphenylphosphine)copper(I) (55.9 mg, 60 µmol) were added under nitrogen atmosphere. The reaction mixture was stirred overnight at 23° C, followed by the evaporation of dichloromethane in vacuo (10 mbar). The crude product was purified by column chromatography (silica gel, methanol/dichloromethane, 2.5/100, v/v, R_f = 0.64) and recrystallization from acetone. The precipitated product was finally washed with cold acetone. The product **2a** was obtained as orange crystals (71.6 mg, 113 µmol, yield: 57%). ^{1}H NMR (400 MHz, $CDCl_3$) δ = 7.8 (d, J = 7.6 Hz, 2H), 7.36-7.22 (m, 8H), 5.42 (br. s, 2H), 5.18 (s, 2H), 5.02 (s, 2H), 4.66 (br. s, 2H), 4.11 (s, 5H), 4.03 (s, 2H), 1.36 (s, 12H). ^{13}C NMR (100 MHz, $CDCl_3$) δ = 154.86, 145.58, 138.99, 135.01, 134.51, 129.04, 128.66, 128.00, 127.38, 122.48, 100.58, 83.86, 68.95, 67.54, 64.58, 62.87, 54.09, 46.14, 24.84. High resolution mass spectroscopy (HRMS) using APPI as an ionization method: calcd for $C_{34}H_{38}BFeN_4O_4$ ($[M+H]^+$): 633.2336; found m/z: 633.2334. Elemental analysis: calcd (%) for $C_{34}H_{37}BFeN_4O_4$: C 64.58, H 5.90, N 8.86; found: C 64.67, H 5.44, N 8,51.

Prodrug **2b** Compound **S1** (1.00 g, 2 mmol), (2-azidoethyl)benzene (0.59 g, 4 mmol) and bromotris(triphenylphosphine)-copper(I) (372.7 mg, 400.6 µmol) were dissolved in dichloromethane (30 mL) and stirred under nitrogen atmosphere at 25° C for twenty four hours followed by evaporation of dichloromethane in vacuo (10 mbar). The crude product was purified by column chromatography (silica gel, dichloromethane/acetone, 95/5, v/v, R_f = 0.48). Pure **2b** was obtained as orange solid (854 mg, 1.32 mmol, yield: 66%). ^{1}H NMR (300 MHz, $CDCl_3$) δ = 7.85 (d, J = 7.9 Hz, 2H), 7.39-7.25 (m, 6H), 7.09 (d, J = 5.5 Hz, 2H), 5.20 (s, 2H), 5.09 (s, 2H), 4.52-4.28 (m, 11H) 3.17 (br. s, 2H), 1.38 (s, 12H). ^{13}C NMR (100 MHz, dmso-d_6) δ = 143.98, 139.68, 137.53, 134.51, 134.21, 128.61, 128.34,

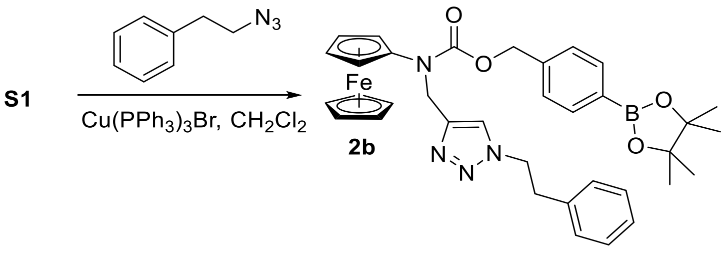

127.11, 126.51, 123.6, 100.55, 83.65, 68.74, 66.71, 64.14, 62.38, 50.39, 45.43, 35.76, 24.62. HRMS (APPI): calcd for $C_{35}H_{39}BFeN_4O_4$ ([M-e$^-$]$^+$): 646.2415; found m/z: 646.2422. Elemental analysis: calcd (%) for $C_{35}H_{39}BFeN_4O_4$: C 65.02, H 6.08, N 8.67; found: C 64.68, H 6.29, N 8.88.

Prodrug **2c** Intermediate **S2** (300.0 mg, 436.4 µmol), 3-phenylpropyne (152.1 mg, 1.31 mmol) and bromotris(triphenyl-phosphine)copper(I) (81.2 mg, 87.3 µmol) were dissolved in dichloromethane (15 mL) and stirred under nitrogen atmosphere at 25° C for 24 hours followed by evaporation of dichloromethane in vacuo (10 mbar). The crude product **2c** was purified by column chromatography (silica gel, dichloromethane/acetone, 90/10, v/v, R_f = 0.44). The product was obtained as orange solid (260 mg, 323 µmol, yield: 74%). ^{1}H NMR (400 MHz, DMSO-d_6): δ = 7.97 (br. s, 1H), 7.80 (s, 1H), 7.66 (d, J = 7.8 Hz, 2H), 7.35 (d, J = 7.5 Hz, 2H), 7.29-7.18 (m, 9H), 5.55 (s, 2H), 5.51 (s, 2H), 5.17 (s, 2H), 4.91 (s, 2H), 4.55 (br. s, 2H), 4.06 (s, 2H), 4.00 (t, J = 2(x2) Hz, 2H), 3.95 (s, 2H), 1.28 (s, 12H). ^{13}C NMR (100 MHz, CD_3CN) δ = 148.23, 146.46, 140.88, 140.81, 137.27, 137.01, 135.65, 129.55, 129.52, 129.47, 129.45, 128.29, 127.23, 123.89, 122.94, 84.94, 69.83, 68.07, 65.37, 63.51, 54.02, 53.87, 40.52, 32.48, 25.22. HRMS (APPI): calcd for $C_{44}H_{47}BFeN_7O_4$ [M+H]$^+$: 804.3135; found m/z: 804.3129. Elemental analysis: calcd (%) for $C_{44}H_{46}BFeN_7O_4$: C 65.77, H 5.77, N 12.20 found: C 65.70, H 5.82, N 12.12.

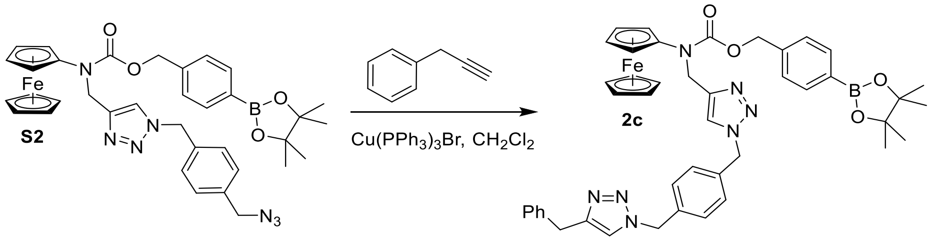

Prodrug **2d** Prodrug **2d** was obtained analogously to prodrug **2c** except that 4-phenylbutyne (136.4 mg, 1.05 mmol) was used in place of 3-phenylpropyne. The purified product was isolated as orange crystals (220 mg, 270 µmol, yield: 62%). TLC (silica gel, eluent

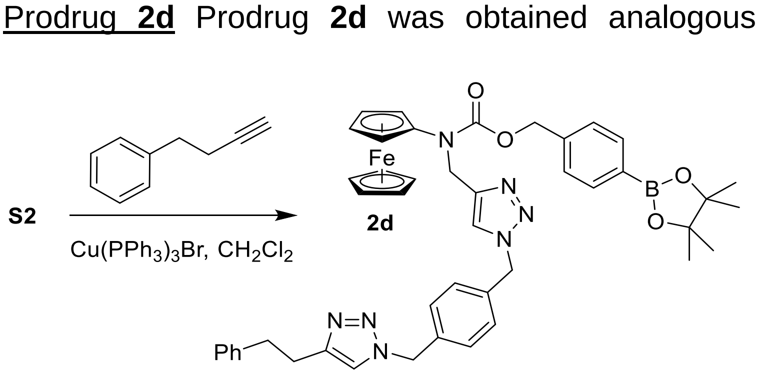

methanol/ dichloromethane, 2.5/97.5, v/v) R_f = 0.22. ^{1}H NMR (300 MHz, Acetone-d_6): δ = 7.74 (d, J = 8 Hz, 4H), 7.51 (s, 1H), 7.41-7.14 (m, 10H), 5.62-5.55 (m, 4H), 5.20 (s, 2H), 4.99 (s, 2H), 4.65 (br. s, 2H), 4.09-4.06 (m, 5H), 3.99-3.98 (m, 2H), 2.93 (s, 4H), 1.34 (s, 12H). ^{13}C NMR (75 MHz, Acetone-d_6) δ = 148.02, 146.26, 142.44, 140.89, 137.52, 137.06, 135.76, 132.68, 129.79, 129.46, 129.24, 129.19, 128.21, 126.84, 123.98, 122.46, 102.18, 84.73, 69.77, 67.92, 65.19, 63.52, 53.89, 53.64, 46.53, 36.28, 28.38, 25.30. HRMS (ESI-TOF): calcd for $C_{45}H_{48}BFeN_7O_4$ [M-e$^-$]$^+$: 817.3205; found m/z: 817.3212. Elemental analysis: calcd (%) for $C_{45}H_{48}BFeN_7O_4$: C 66.11, H 5.92, N 11.99; found: C 66.25, H 5.68, N 11.89.

<u>Intermediate **S3**</u> A mixture containing 4-(hydroxymethyl)benzoic acid (2000 mg, 13.1 mmol) and 1-[3-(dimethylamino)propyl]-3-ethylcarbodiimide hydrochloride (EDC, 2827 mg, 14.5 mmol) in dichloromethane (50 mL) was stirred for 20 min at 23 ^{o}C (active ester intermediate can be monitored by TLC using ethyl acetate as an eluent), followed by adding dimethylamine (2 M in THF, 7.9 mL, 15.8 mmol). After stirring for 10 min, the reaction mixture was concentrated by partial solvent evaporation and purified by column chromatography (silica gel, dichloromethane/methanol, 25/1, v/v) to give S3 as a colorless oil (750 mg, 4.18 mmol, 32%). TLC (silica gel, dichloromethane/methanol, 10/1, v/v) R_f = 0.40. ^{1}H NMR (400 MHz, $CDCl_3$) δ = 7.55 – 7.29 (m, 4H), 4.78 (s, 2H), 3.86 (s, 1H), 3.19 (s, 6H). ^{13}C NMR (100 MHz, $CDCl_3$) δ = 171.93, 143.32, 134.60, 127.08, 126.46, 64.17, 39.69, 35.54. HRMS (APPI): calcd for $C_{10}H_{14}NO_2$ [M+H]$^+$: 180.1019; found m/z: 180.1017.

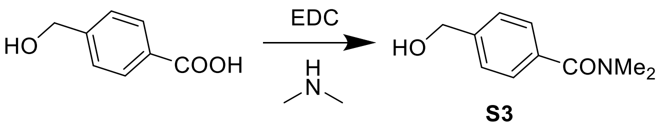

S3

<u>Intermediate **S4**</u> **S3** (32 mg, 0.17 mmol) and ferrocenylcarbonylazide (40 mg, 0.16 mmol) were dissolved under nitrogen atmosphere in anhydrous toluene (3 mL) and heated to 105 °C. The reaction mixture was stirred for a further 2 h at 105 °C. The solvent was removed by evaporation and the crude product was purified by column

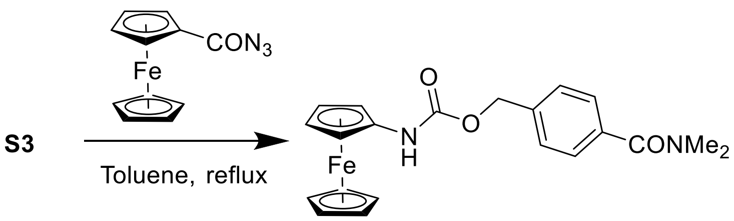

S4

chromatography (silica gel, ethyl acetate, R_f = 0.46). Pure **S4** was obtained as orange foam (65 mg, 0.16 mmol, yield: 97%). ^{1}H NMR (400 MHz, Acetone-d_6) δ = 8.05 (s, 1H), 7.63 – 7.29 (m, 4H), 5.18 (s, 2H), 4.56 (s, 2H), 4.10 (s, 5H), 3.93 (t, *J*=1.9, 2H), 2.99 (s, 6H). ^{13}C NMR (100 MHz, Acetone-d_6) δ = 171.00, 154.56, 139.35, 137.58, 128.35, 128.12, 97.73, 69.69, 66.22, 64.62, 61.08, 39.50, 35.20. HRMS (APPI): calcd for $C_{21}H_{22}FeN_2O_3$ $[M-e^-]^+$: 406.0974; found m/z: 406.0982.

<u>Intermediate **S5**</u> **S4** (300 mg, 0.74 mmol) was dissolved in anhydrous DMF (3.7 mL) under a nitrogen atmosphere. Then Cs_2CO_3 (722 mg, 2.22 mmol), CsI (190 mg, 0.74 mmol) were added to the solution. After stirring for 0.5 h at 22 °C, propargyl chloride (110 mg, 107 uL, 1.48 mmol) was added and the mixture was stirred overnight. After dilution with ethyl acetate and filtration, the solvent was removed and the crude product was purified by column chromatography (silica gel, iso-hexane/acetone, 3/1, v/v). Pure **S5** was obtained as orange oil (260 mg, 0.58 mmol, yield: 79%). TLC (silica gel, dichloromethane/methanol, 20/1, v/v) R_f = 0.20. ^{1}H NMR (400 MHz, Acetone-d_6) δ = 7.58 – 7.39 (m, 4H), 5.26 (s, 2H), 4.60 (s, 2H), 4.57 (d, *J*=2.5, 2H), 4.19 (s, 5H), 4.08 – 4.00 (m, 2H), 2.99 (s, 6H), 2.89 (s, 1H). ^{13}C NMR (100 MHz, Acetone-d_6) δ = 170.95, 158.72, 138.83, 137.74, 128.41, 128.15, 81.39, 73.64, 69.85, 67.64, 65.25, 63.38, 40.31, 39.39, 35.06. HRMS (ESI-TOF): calcd for $C_{24}H_{24}FeN_2O_3$ $[M-e^-]^+$: 444.1131; found m/z: 444.1117.

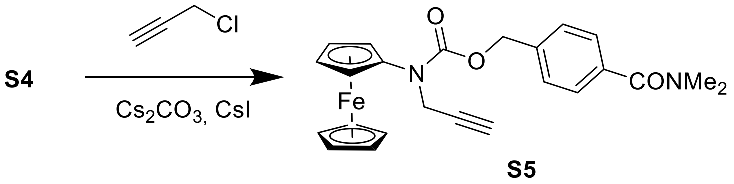

<u>Synthesis of intermediate **S6**</u> Intermediate **S6** was obtained analogously to intermediate **S2** except that intermediate **S5** (260 mg, 0.59 mmol) was used in place of **S1** and iso-hexane/acetone (2/1, v/v) was used in place of dichloromethane/acetone (95/5, v/v). The purified product was

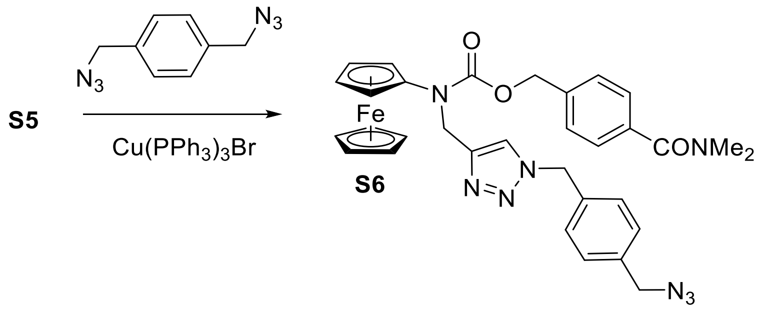

obtained as orange solid (178 mg, 0.28 mmol, yield: 48%). TLC (silica gel, iso-hexane/acetone, 1/1, v/v) R_f = 0.40. ^{1}H NMR (400 MHz, Acetone-d_6) δ = 7.79 (s, 1H), 7.60 – 7.21 (m, 8H), 5.61 (s, 2H), 5.22 (s, 2H), 5.01 (s, 2H), 4.66 (s, 2H), 4.43 (s, 2H), 4.08 (s, 5H), 3.99 (t, *J*=2.0, 2H), 3.02 (s, 6H). ^{13}C NMR (75 MHz, Acetone-d_6) δ = 170.93, 154.74, 146.09, 138.78, 137.68, 136.97, 136.91, 129.65, 129.18, 128.58, 128.14, 123.94, 102.08, 69.64, 67.47, 65.08, 63.43, 54.56, 53.79, 46.45, 39.47, 35.15. HRMS (ESI-TOF): calcd for $C_{32}H_{32}FeN_8O_3$ $[M-e^-]^+$: 632.1942; found m/z: 632.1935.

2c_c Control compound **2c**_c was obtained analogously to **2c** except that intermediate **S6** (70 mg, 0.11 mmol) was used in place of **S2** and iso-hexane/acetone (1/1, v/v) was used in place of dichloromethane/acetone (90/10, v/v). The purified product was obtained as orange solid (48 mg, 0.06 mmol, yield: 58%). TLC (silica gel, iso-hexane/acetone, 1/2, v/v) R_f = 0.43. ^{1}H NMR (400 MHz, Acetone-d_6) δ = 8.06 – 7.03 (m, 15H), 5.57 (s, 2H), 5.55 (s, 2H), 5.20 (s, 2H), 5.01 (s, 2H), 4.75 (s, 2H), 4.12 (s, 5H), 4.07 (s, 2H), 3.99 (s, 2H), 2.98 (s, 6H). ^{13}C NMR (100 MHz, Acetone-d_6) δ = 171.00, 147.98, 146.30, 140.72, 138.82, 137.74, 137.31, 137.07, 129.48, 129.33, 129.27, 129.25, 128.61, 128.17, 127.01, 123.91, 122.87, 70.28, 67.47, 65.51, 63.39, 53.77, 53.65, 46.26, 39.52, 35.19, 32.59. HRMS (ESI-TOF): calcd for $C_{41}H_{40}FeN_8O_3$ $[M-e^-]^+$: 748.2567; found m/z: 748.2569. Elemental analysis: calcd (%) for $C_{41}H_{40}FeN_8O_3$: C 65.78, H 5.39, N 14.97 found: C 65.50 H 5.24 N 14.79.

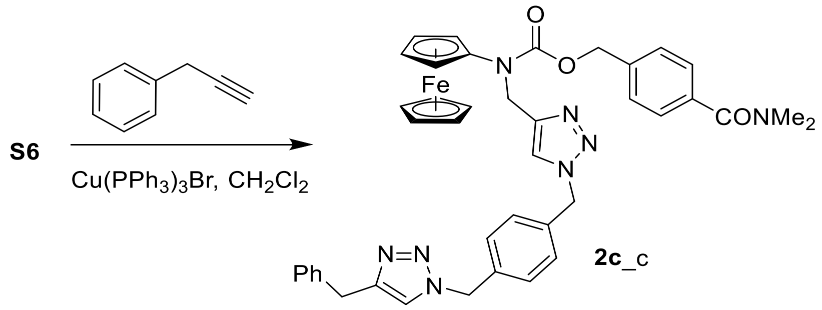

Intermediate **S8** Intermediate **S8** was obtained analogously to **S1** except that N-Boc-protected aminoferrocene **S7** was used in place of 4-(ferrocenylaminocarbonyloxymethyl)phenylboronic acid pinacol ester. The product was isolated as orange oil (49 mg, 145 µmol, yield 87%). TLC (silica gel, eluent cyclohexane/acetone, 4/1, v/v) R_f = 0.64. ^{1}H NMR (400 MHz, $CDCl_3$) δ = 4.68 (d, *J* = 152.5, 10H), 2.25 (s, 1H), 1.48 (s, 9H). ^{13}C NMR (100 MHz, $CDCl_3$) δ = 81.32, 71.81,

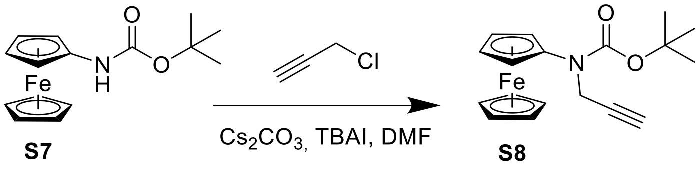

62.53, 39.49, 28.40. HRMS (ESI-TOF): calcd for $C_{18}H_{21}FeNO_2$ [M-e$^-$]$^+$: 339.0916; found m/z: 339.0921.

Intermediate **S9** Intermediate **S9** was obtained analogously to prodrug **2a** except that

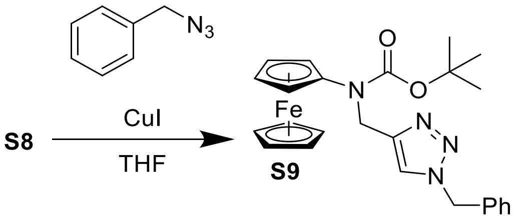

intermediate **S8** was used in place of **S1**. The product was isolated as orange powder (469 mg, 990 µmol, yield 80%). TLC (silica gel, eluent cyclohexane/acetone, 4/1, v/v) R_f = 0.31. ^1H NMR (400 MHz, Acetone-d6) δ = 7.85 (s, 1H), 7.45 – 7.27 (m, 5H), 5.64 (s, 2H), 4.90 (s, 2H), 4.64 (s, 2H), 4.07 (s, 5H), 4.00 – 3.92 (m, 2H), 1.44 (s, 9H). ^{13}C NMR (100 MHz, Acetone-d6) δ = 146.63, 137.10, 129.67, 129.08, 128.90, 123.75, 81.14, 69.61, 64.92, 63.20, 54.17, 46.11, 28.46. HRMS (ESI-TOF): calcd for $C_{25}H_{28}FeN_4O_2$ [M-e$^-$]$^+$: 472.1556; found m/z: 472.1555.

3*2HCl Intermediate **S9** (50 mg, 110 µmol) was grinded to powder and hydrochloric acid

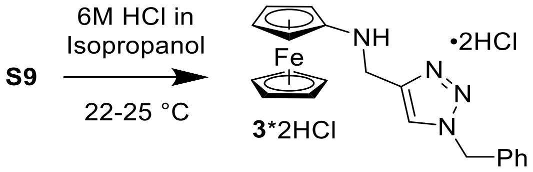

in iso-propanol (5.5 M, 1.7 mL) was added. This operation was conducted under nitrogen. The mixture was stirred at 22-25 °C overnight. The obtained product was precipitated. It was filtered and dried in vacuo (10 mbar). The product was obtained as orange powder (32 mg, 70 µmol, yield 68%). TLC (silica gel, eluent dichloromethane/methanol, 20/3, v/v) R_f = 0.65. ^1H NMR (400 MHz, MeOD) δ = 7.99 (s, 1H), 7.41 – 7.28 (m, 5H), 5.61 (s, 2H), 4.49 (s, 2H), 4.41 – 4.31 (m, 7H), 4.26 – 4.21 (m, 2H). ^{13}C NMR (100 MHz, MeOD) δ = 136.59, 130.03, 129.68, 129.17, 127.17, 93.55, 71.10, 68.61, 64.17, 55.01, 47.82. HRMS (ESI-TOF): calcd for $C_{20}H_{20}FeN_4$ [M-e$^-$]$^+$: 372.1032; found m/z: 372.1032. Elemental analysis: calcd (%) for $C_{20}H_{22}Cl_2FeN_4$: C 53.96, H 4.98, N 12.59; found: C 54.03, H 5.16, N 12.62.

Intermediate **S10** Intermediate **S10** was obtained analogously to intermediate **S8** except

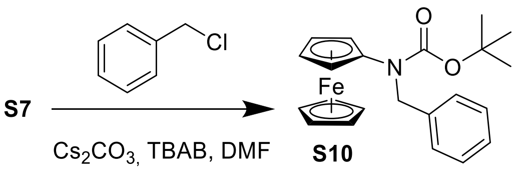

that benzylchloride was used in place of propargylchloride. The product was isolated as orange oil (400 mg, 1.02 mmol, yield 77%). TLC (silica gel, eluent cyclohexane/acetone, 4/1, v/v) R_f = 0.48. ^1H NMR (300 MHz, dmso-d6) δ = 7.50 – 7.15 (m, 5H), 4.92 (s, 2H), 4.43 (s, 2H),

4.13 (s, 5H), 4.00 – 3.92 (m, 2H), 1.47 (s, 9H). ^{13}C NMR (75 MHz, DMSO-d_6) δ = 140.48, 129.23, 127.55, 127.21, 102.85, 80.95, 69.61, 64.83, 63.07, 53.82, 28.45. HRMS (ESI-TOF): calcd for $C_{22}H_{25}FeNO_2$ [M-e$^-$]$^+$: 391.1229; found m/z: 391.1230.

<u>**4***HCl</u> was obtained analogously to **3***2HCl except that **S6** was used in place of **S5**. The

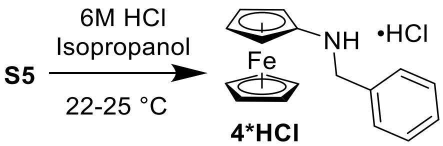

product was obtained as orange powder (302 mg, 920 µmol, yield 90%). TLC (silica gel, eluent dichloromethane/methanol, 20/3, v/v) R_f = 0.79. ^{1}H NMR (300 MHz, MeOD) δ = 7.49 – 7.31 (m, 5H), 4.42 – 4.32 (m, 9H), 4.30 – 4.24 (m, 2H). ^{13}C NMR (75 MHz, MeOD) δ = 131.86, 131.64, 130.80, 130.04, 93.83, 71.09, 68.54, 64.28, 57.52. HRMS (ESI-TOF): calcd for $C_{17}H_{17}FeN$ [M-e$^-$]$^+$: 291.0705; found m/z: 291.0704. Elemental analysis: calcd (%) for $C_{17}H_{18}ClFeN$: C 62.32, H 5.54, N 4.28; found: C 62.21, H 5.63, N 4.31.

^{1}H-NMR, ^{13}C-NMR and high resolution mass spectra (HRMS) of reported intermediates, prodrugs and drugs

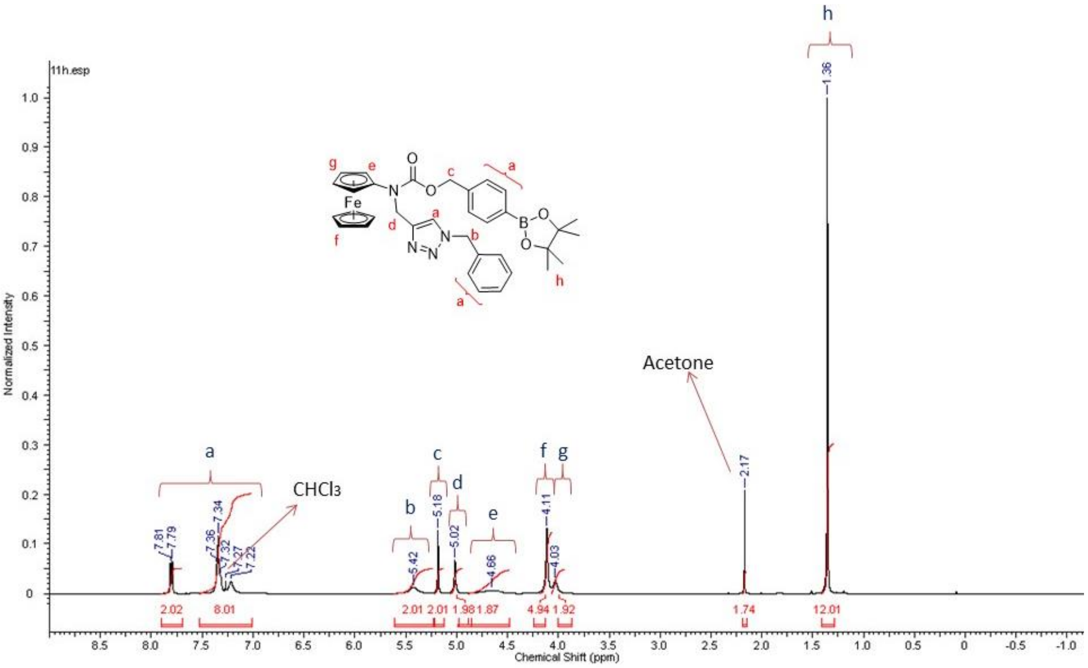

Figure **S1.** ^{1}H-NMR spectrum of prodrug **2a** in $CDCl_3$.

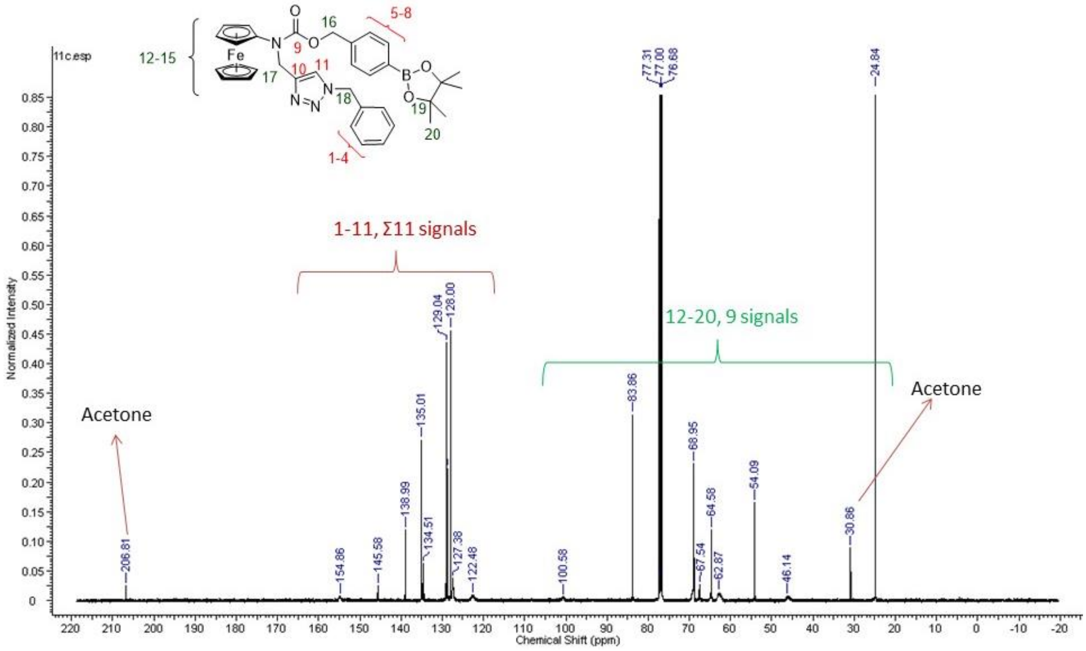

Figure **S2.** ^{13}C-NMR spectrum of prodrug **2a** in $CDCl_3$.

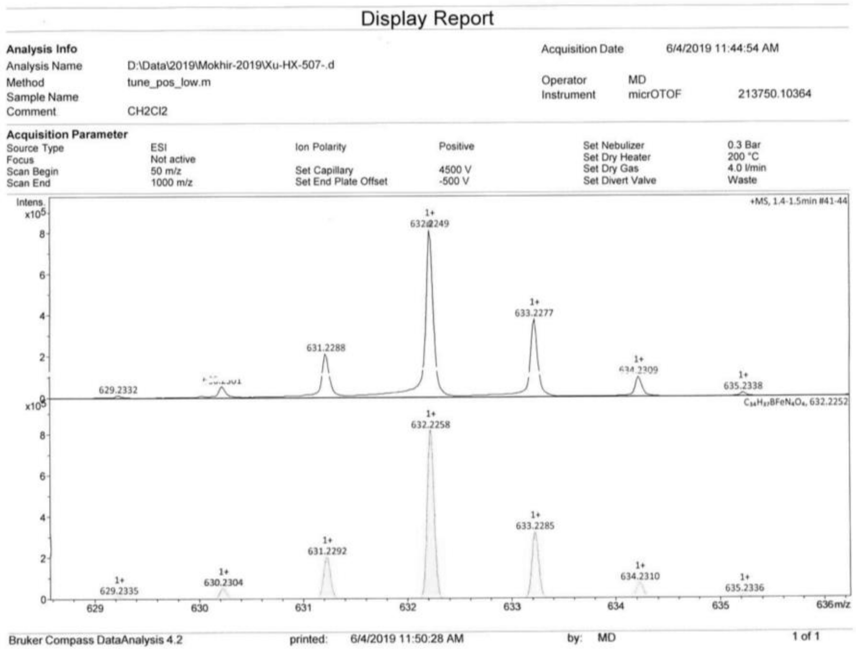

Figure **S3.** High resolution ESI-TOF mass spectrum of prodrug **2a**: upper plot – experimental spectrum; bottom plot – theoretical spectrum.

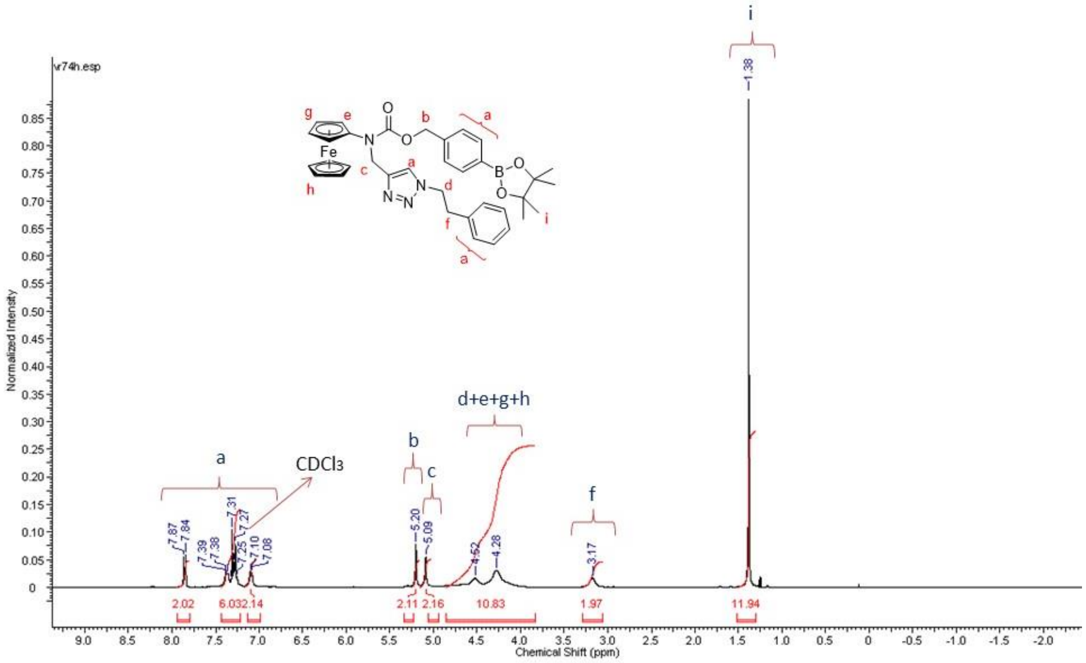

Figure **S4.** ^{1}H-NMR spectrum of prodrug **2b** in $CDCl_3$.

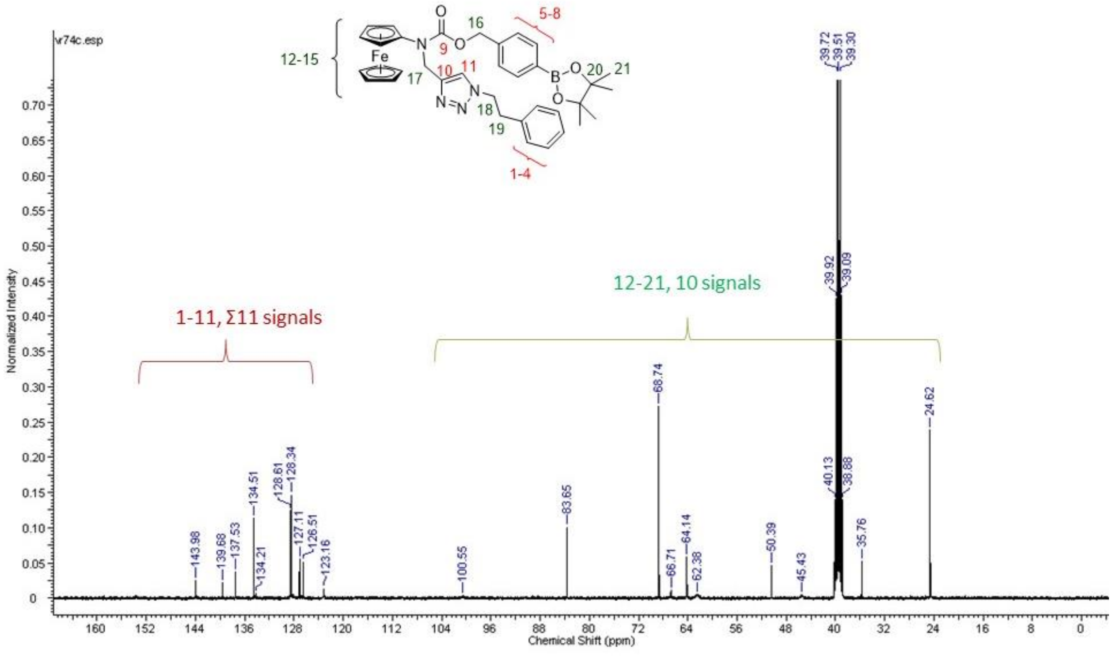

Figure **S5.** ^{13}C-NMR spectrum of prodrug **2b** in DMSO-d_6.

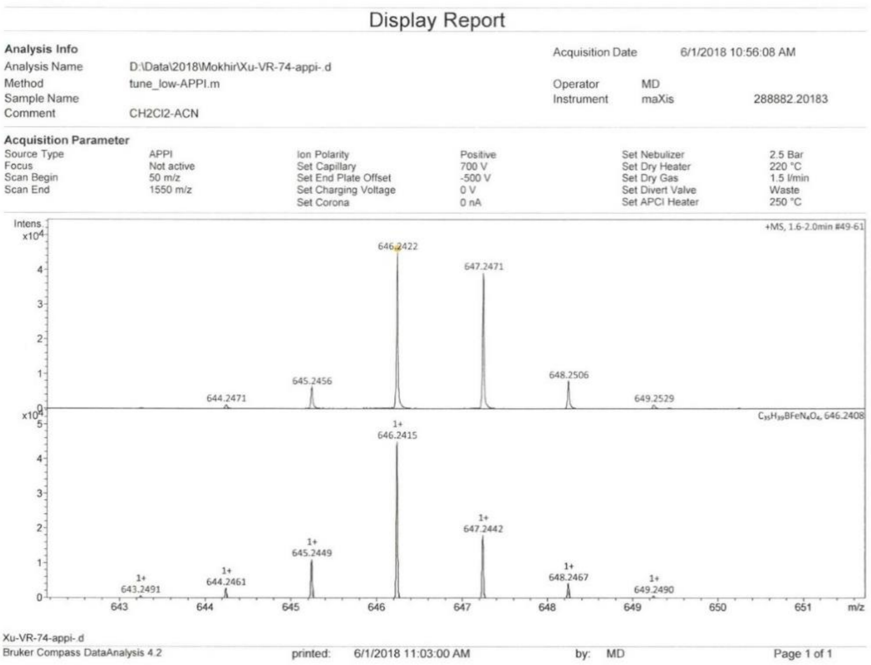

Figure **S6.** High resolution APPI mass spectrum of prodrug **2b**: upper plot – experimental spectrum; bottom plot – theoretical spectrum.

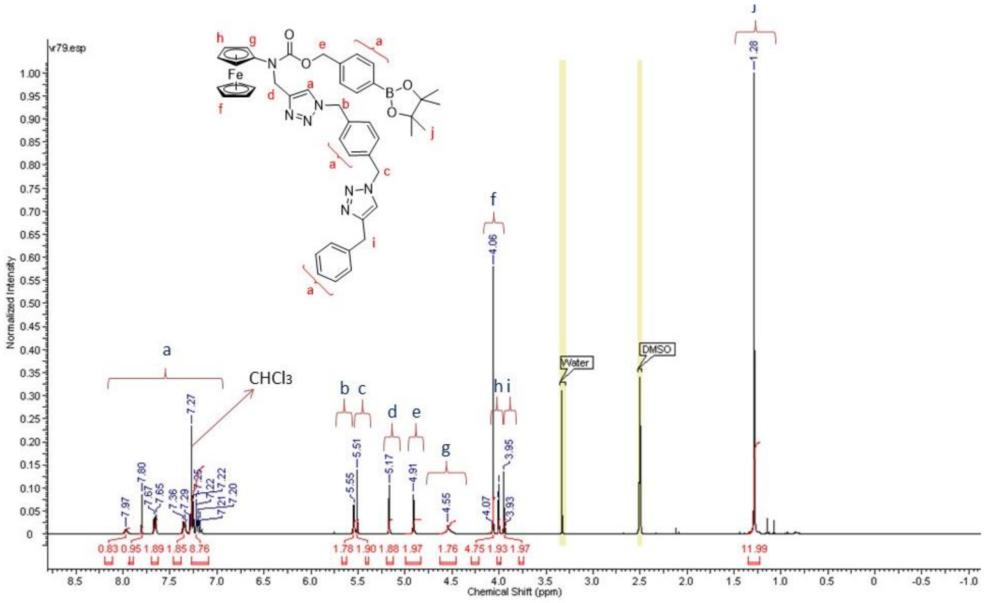

Figure **S7.** ^{1}H-NMR spectrum of prodrug **2c** in DMSO-d_6.

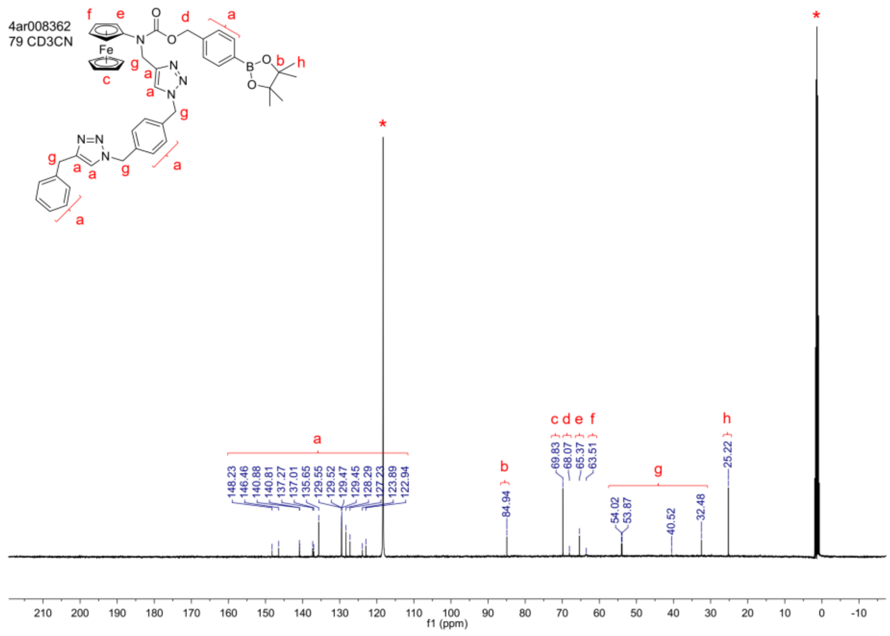

Figure **S8.** ^{13}C-NMR spectrum of prodrug **2c** in CD_3CN.

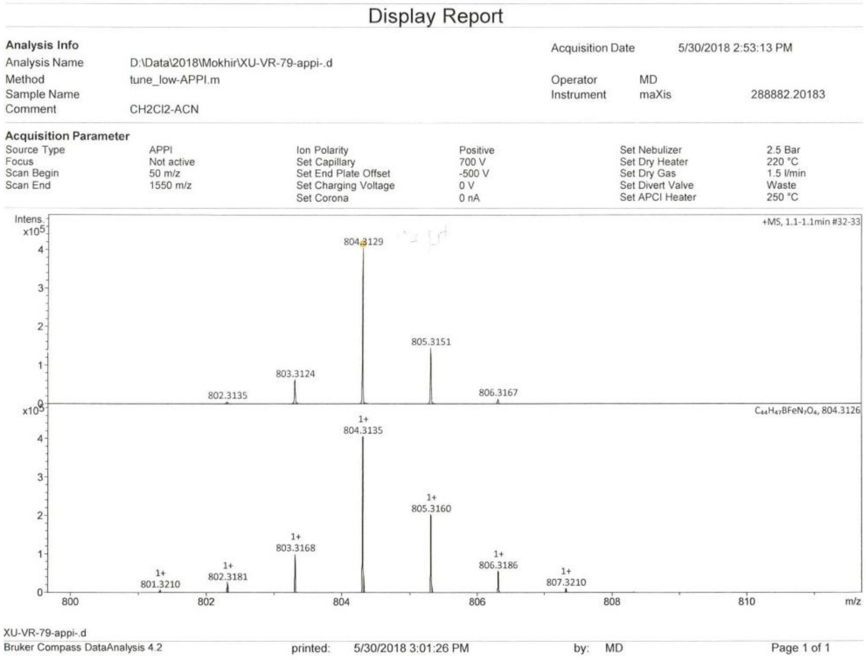

Figure **S9.** High resolution APPI mass spectrum of prodrug **2c**: upper plot – experimental spectrum; bottom plot – theoretical spectrum.

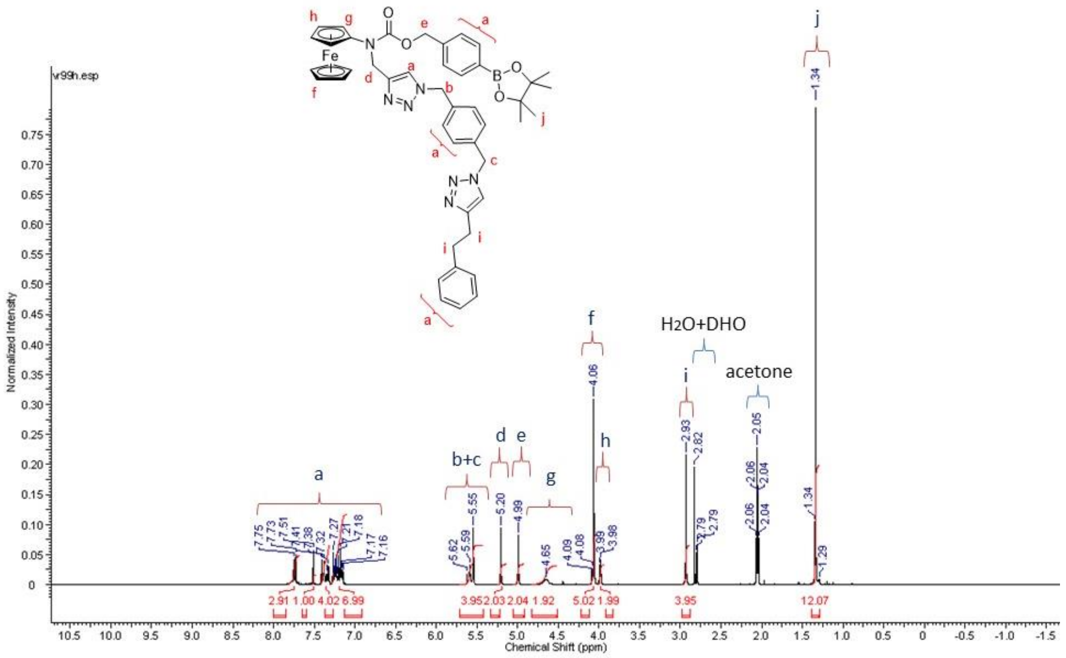

Figure **S10.** ^{1}H-NMR spectrum of prodrug **2d** in acetone-d_6.

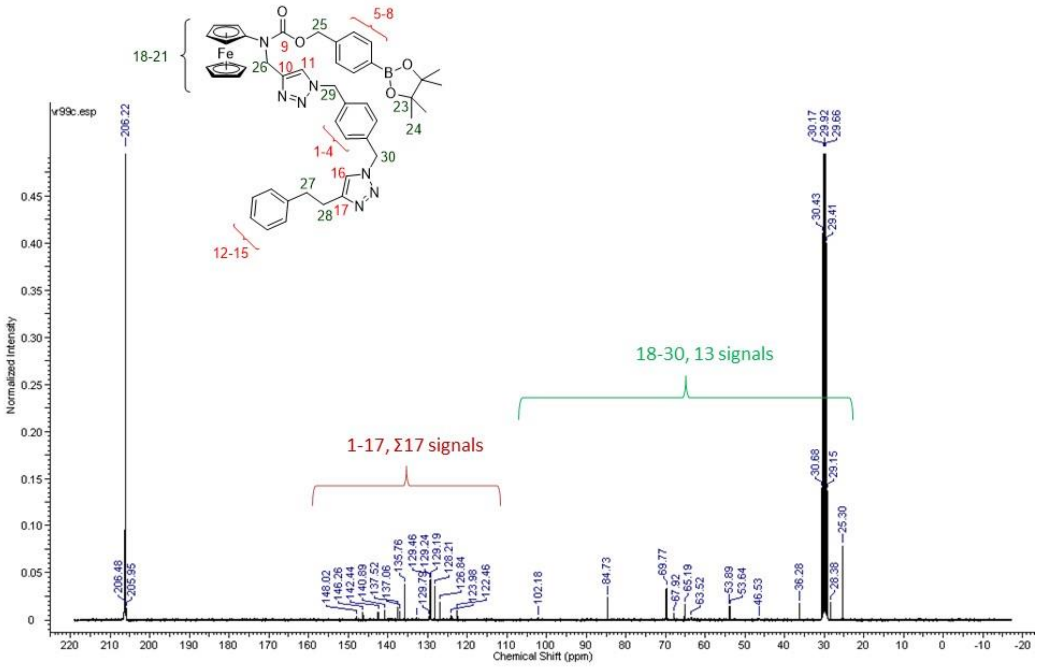

Figure **S11.** ^{13}C-NMR spectrum of prodrug **2d** in acetone-d_6.

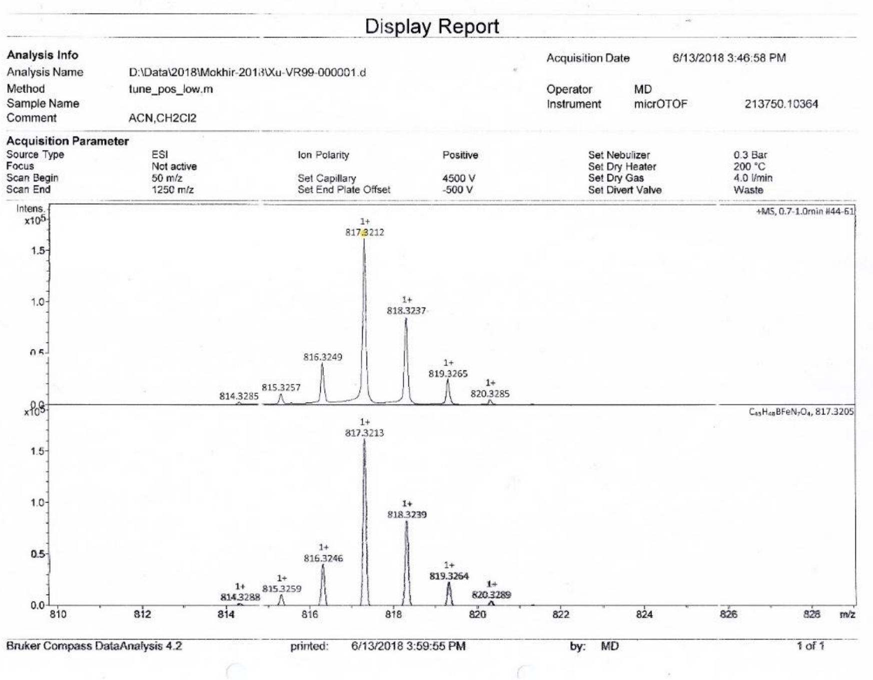

Figure **S12.** High resolution ESI-TOF mass spectrum of prodrug **2d**: upper plot – experimental spectrum; bottom plot – theoretical spectrum.

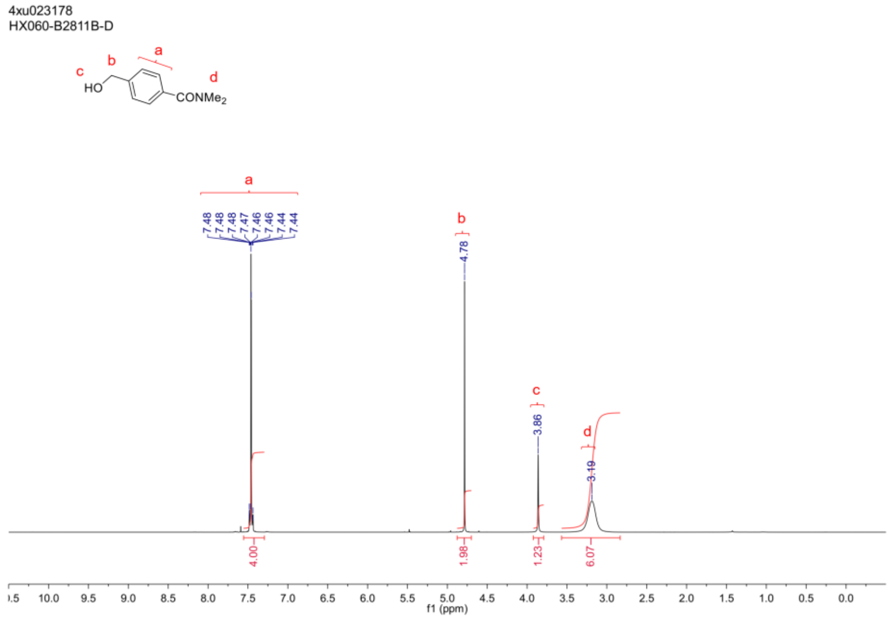

Figure **S13**. ^{1}H-NMR spectrum of intermediate **S3** in $CDCl_3$.

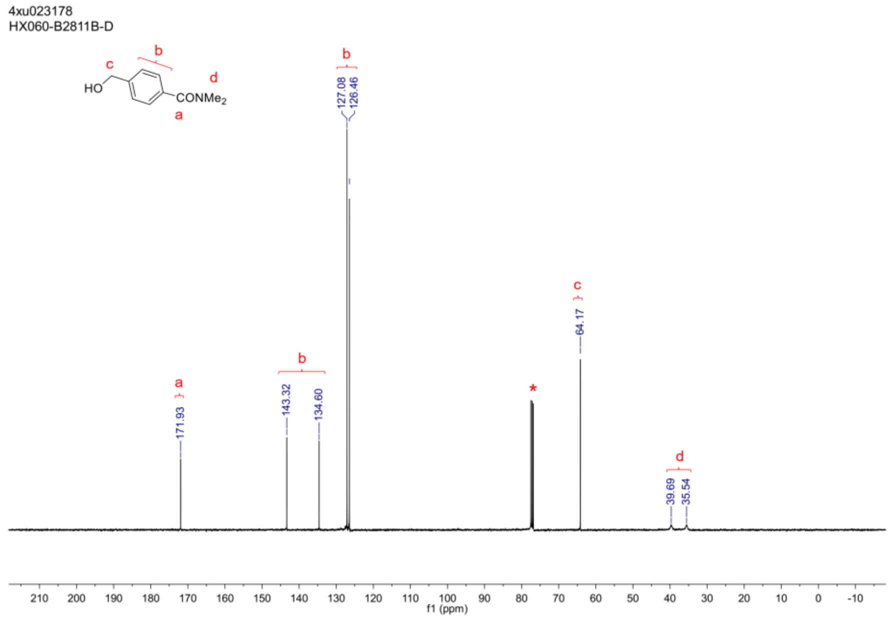

Figure **S14**. ^{13}C-NMR spectrum of intermediate **S3** in $CDCl_3$.

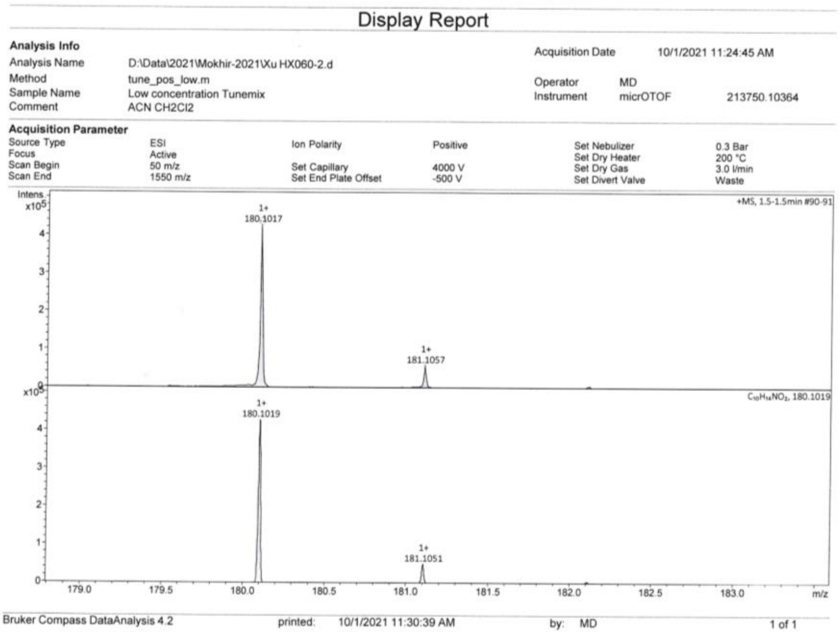

Figure **S15**. High resolution ESI-TOF mass spectrum of intermediate **S3**: upper plot – experimental spectrum; bottom plot – theoretical spectrum.

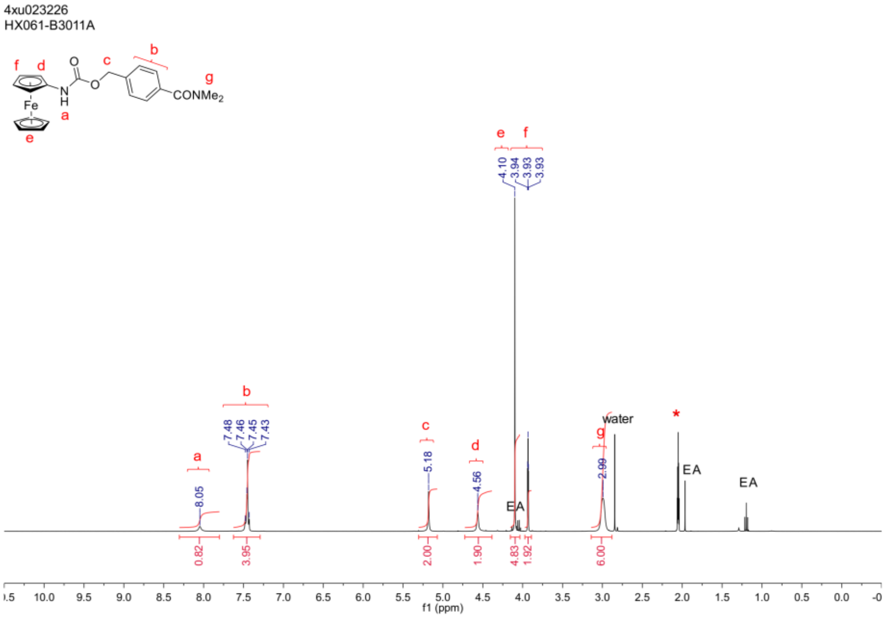

Figure **S16**. ^{1}H-NMR spectrum of intermediate **S4** in acetone-d$_6$.

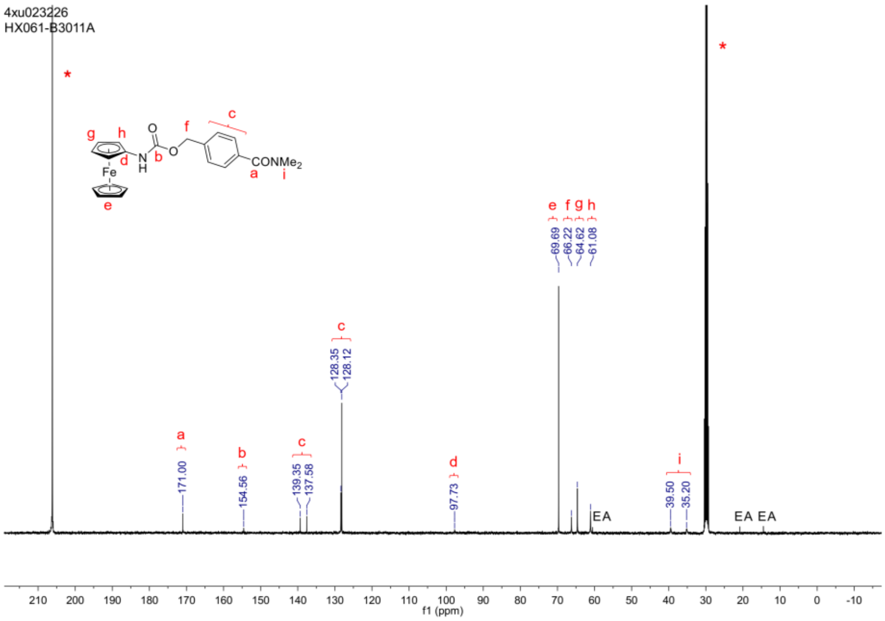

Figure **S17**. ^{13}C-NMR spectrum of intermediate **S4** in acetone-d$_6$.

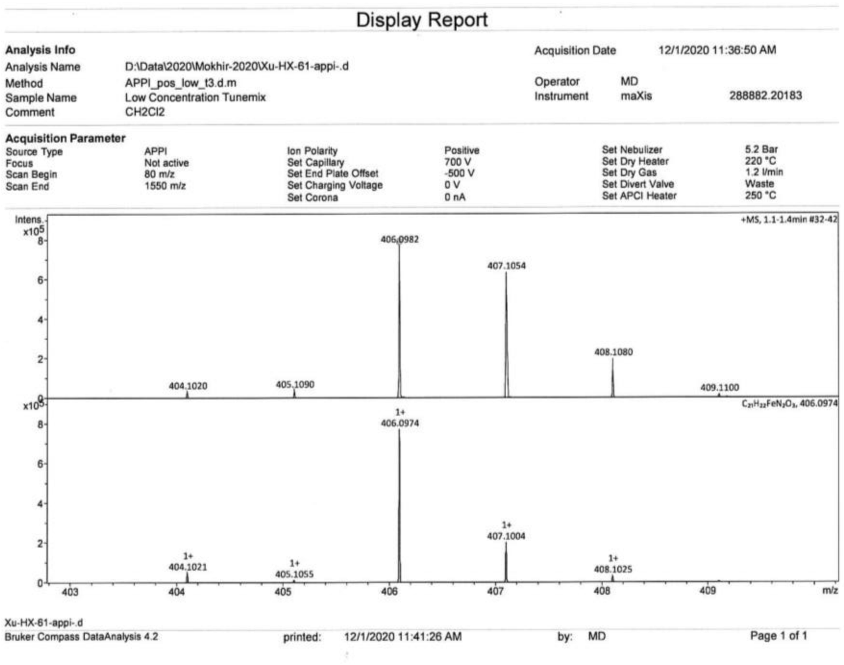

Figure **S18**. High resolution APPI mass spectrum of intermediate **S4**: upper plot – experimental spectrum; bottom plot – theoretical spectrum.

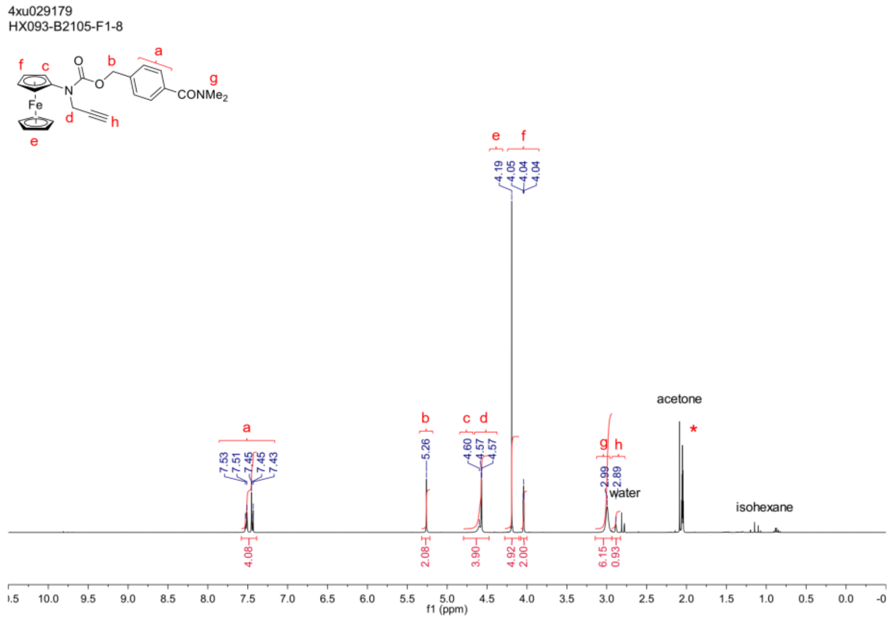

Figure **S19**. ^{1}H-NMR spectrum of intermediate **S5** in acetone-d_6.

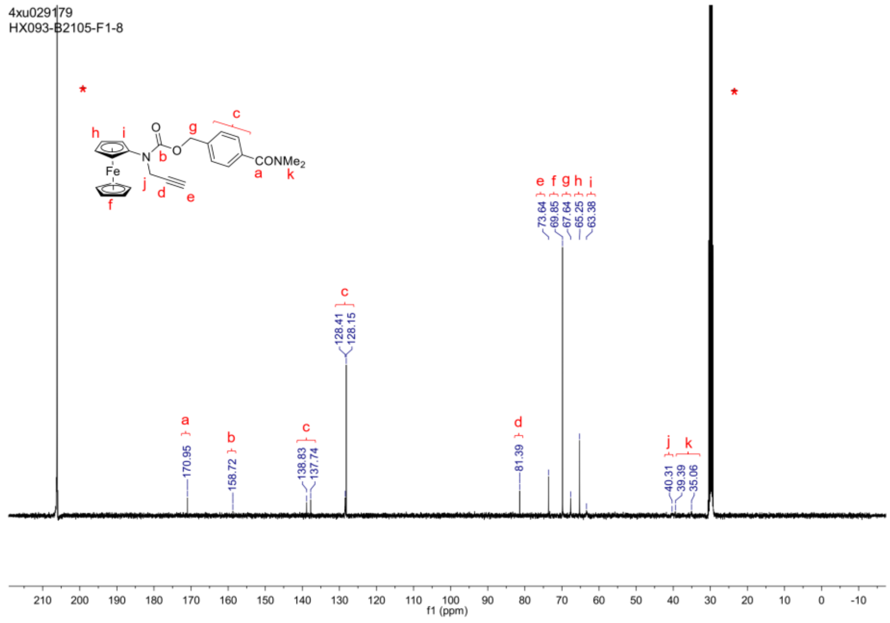

Figure **S20**. ^{13}C-NMR spectrum of intermediate **S5** in acetone-d$_6$.

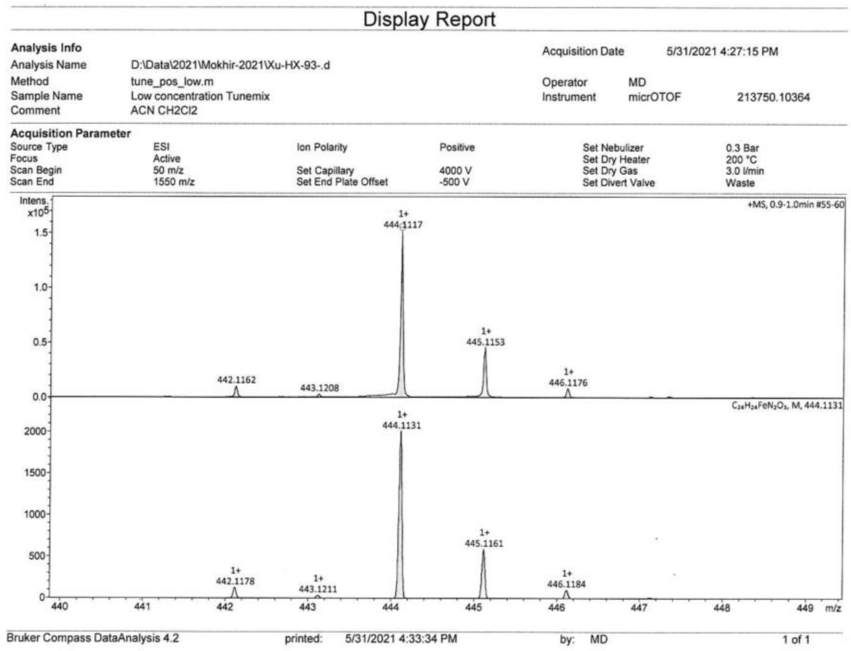

Figure **S21**. High resolution ESI-TOF mass spectrum of intermediate **S5**: upper plot – experimental spectrum; bottom plot – theoretical spectrum.

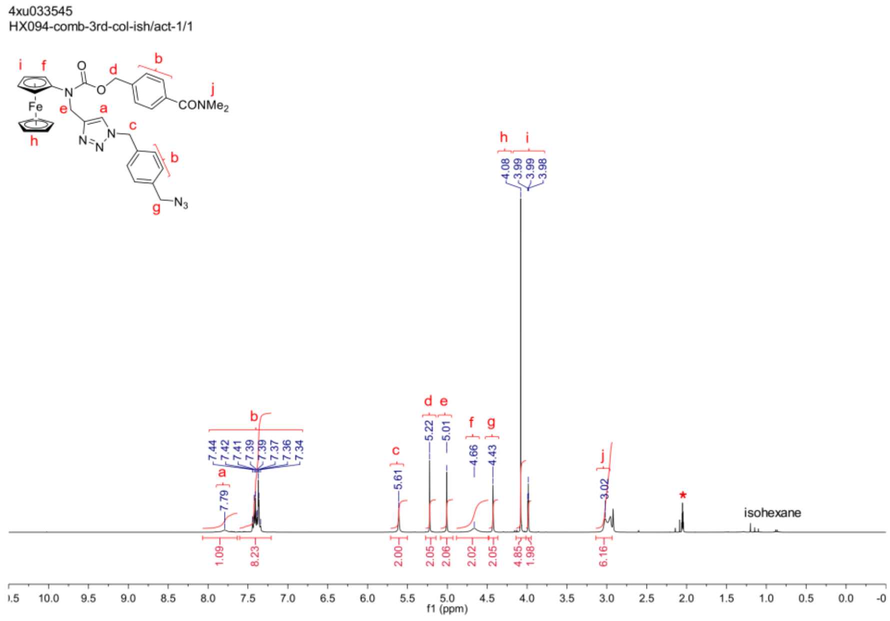

Figure **S22**. ^{1}H-NMR spectrum of intermediate **S6** in acetone-d_6.

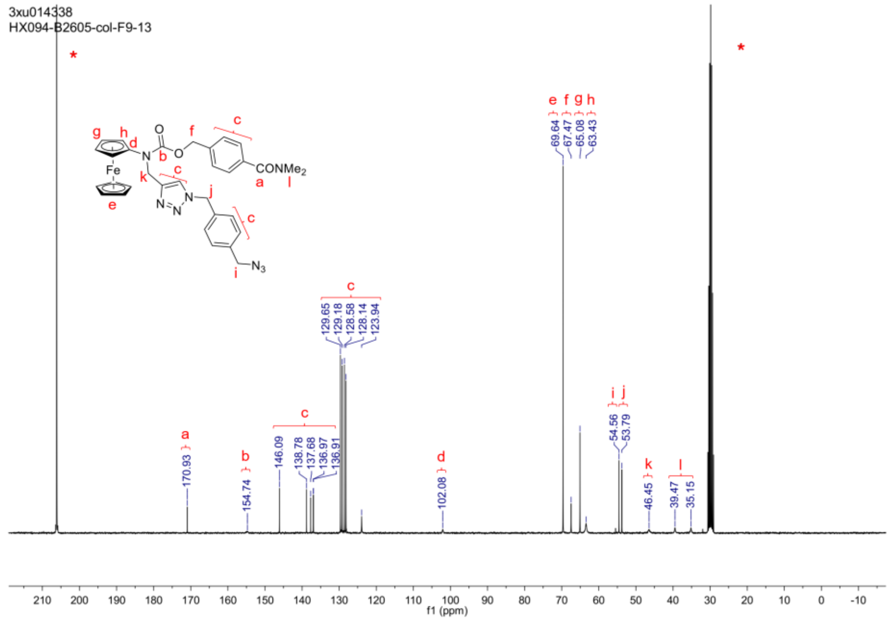

Figure **S23**. ^{13}C-NMR spectrum of intermediate **S6** in acetone-d_6.

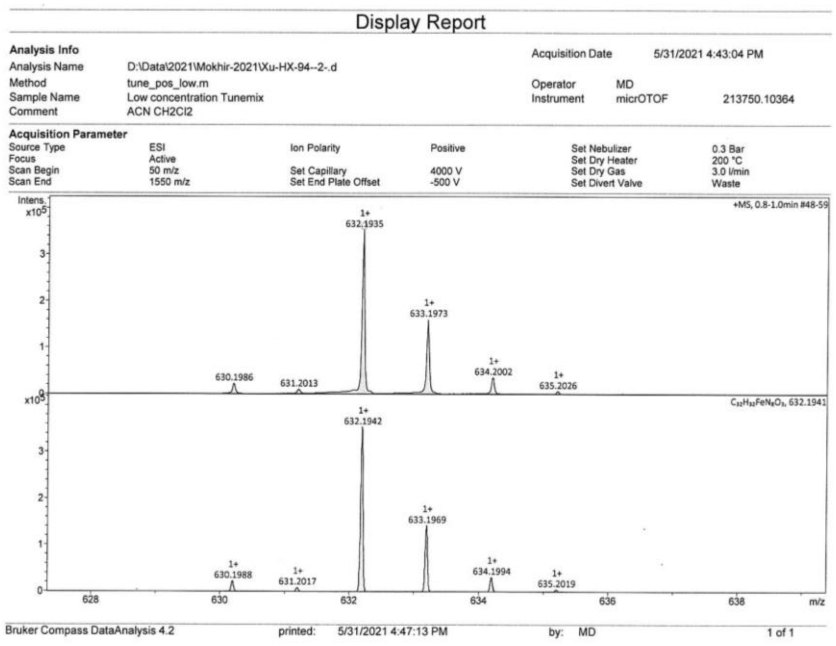

Figure **S24**. High resolution ESI-TOF mass spectrum of intermediate **S6**: upper plot – experimental spectrum; bottom plot – theoretical spectrum.

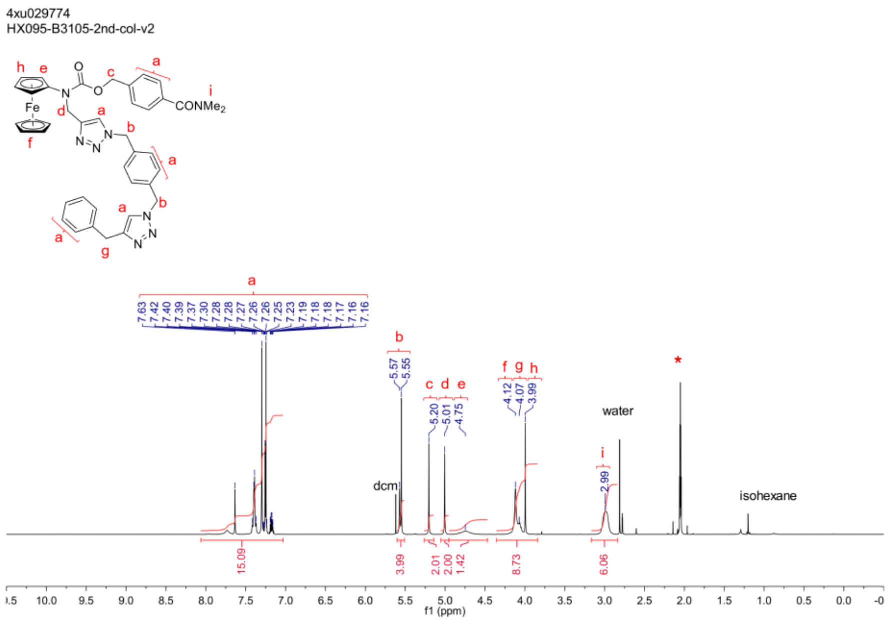

Figure **S25**. ^{1}H-NMR spectrum of intermediate **2c**_c in acetone-d_6.

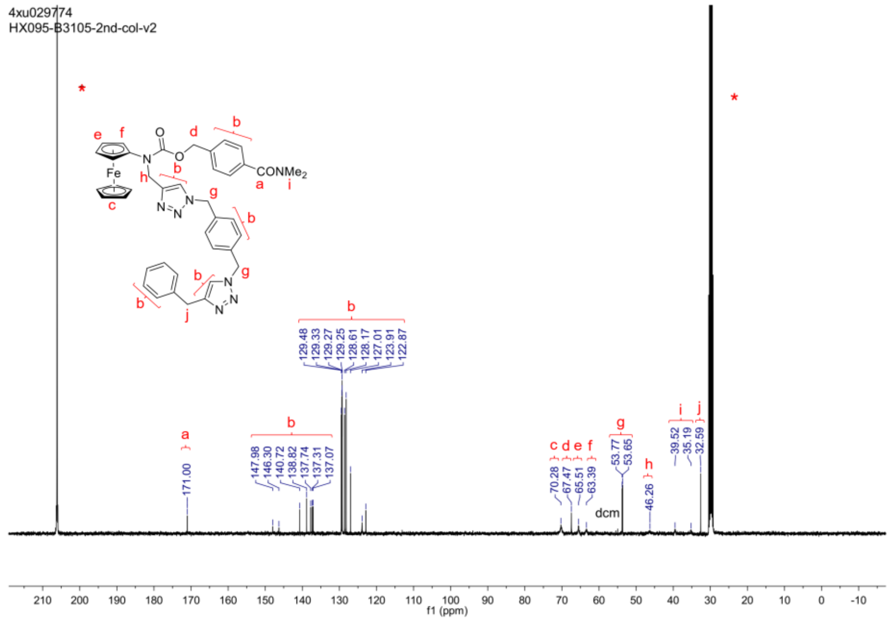

Figure **S26**. ^{13}C-NMR spectrum of compound **2c**_c in acetone-d_6.

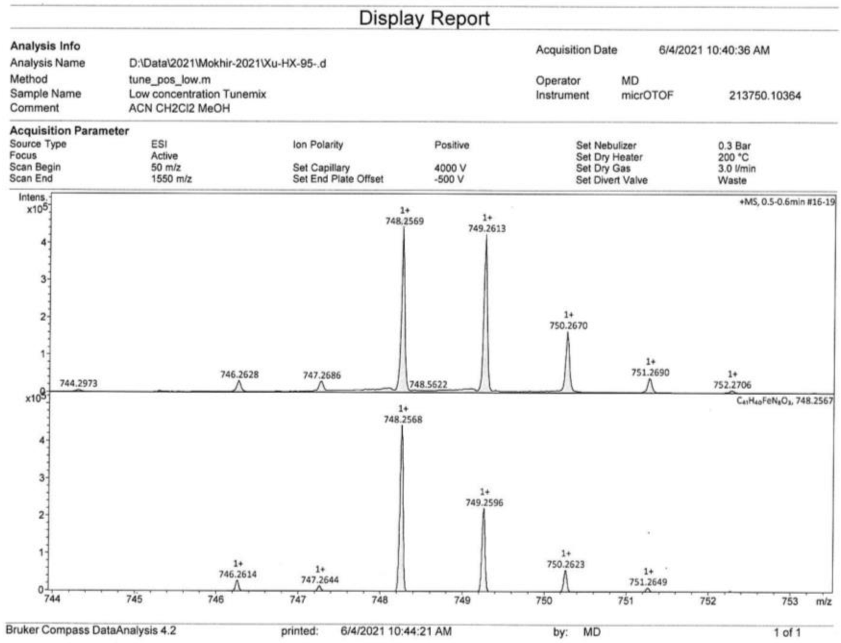

Figure **S27**. High resolution ESI-TOF mass spectrum of **2c**_c: upper plot –experimental spectrum; bottom plot – theoretical spectrum.

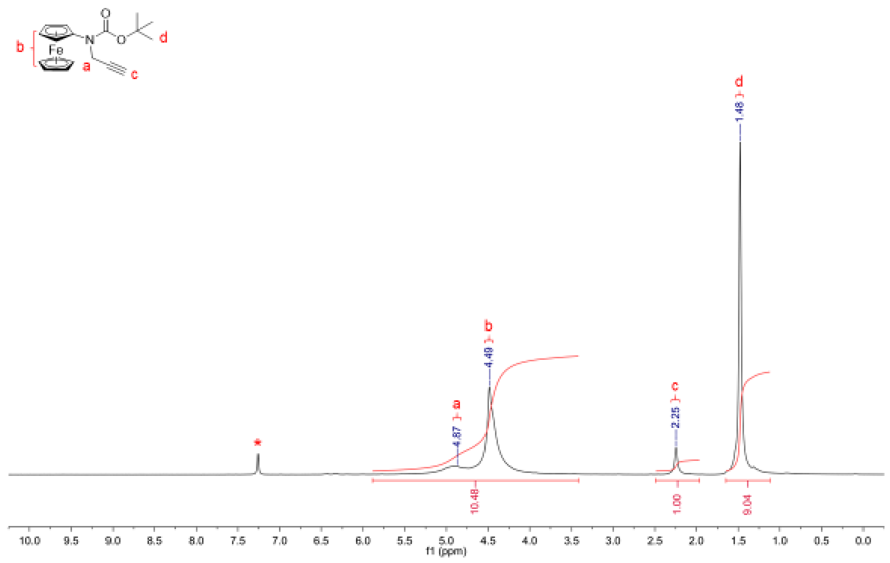

Figure **S28**. ^{1}H-NMR spectrum of intermediate **S8** in $CDCl_3$. NMR solvent residual peaks are marked with *.

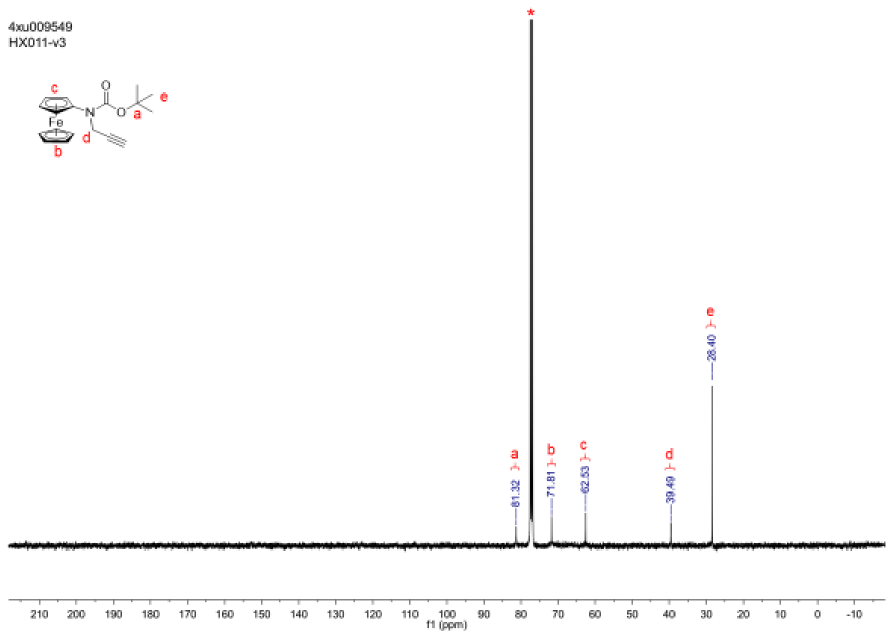

Figure **S29**. ^{13}C-NMR spectrum of intermediate **S8** in $CDCl_3$. NMR solvent residual peaks are marked with *.

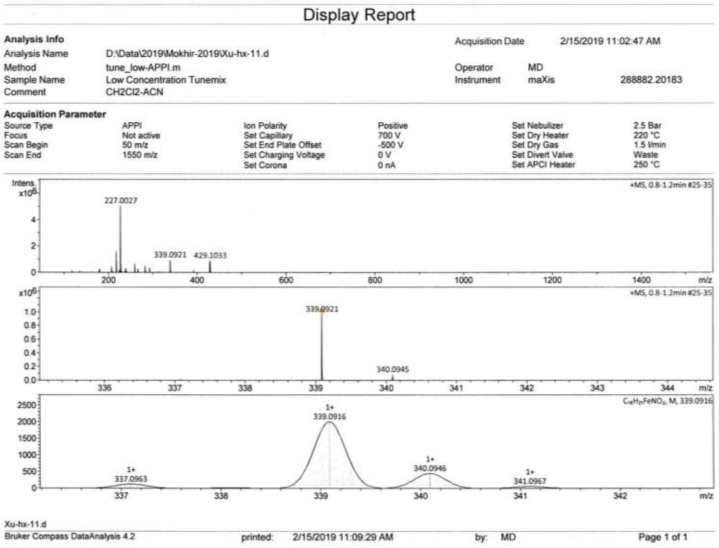

Figure **S30**. High resolution APPI mass spectrum of intermediate **S8**: upper plot – experimental spectrum; bottom plot – theoretical spectrum.

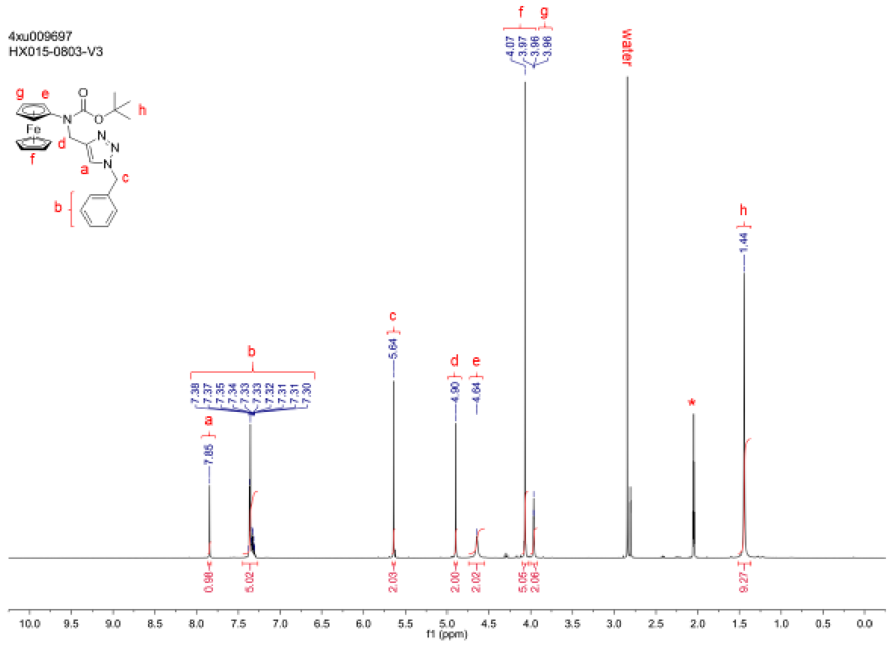

Figure **S31**. ^{1}H-NMR spectrum of intermediate **S9** in acetone-d$_6$. NMR solvent residual peaks are marked with *.

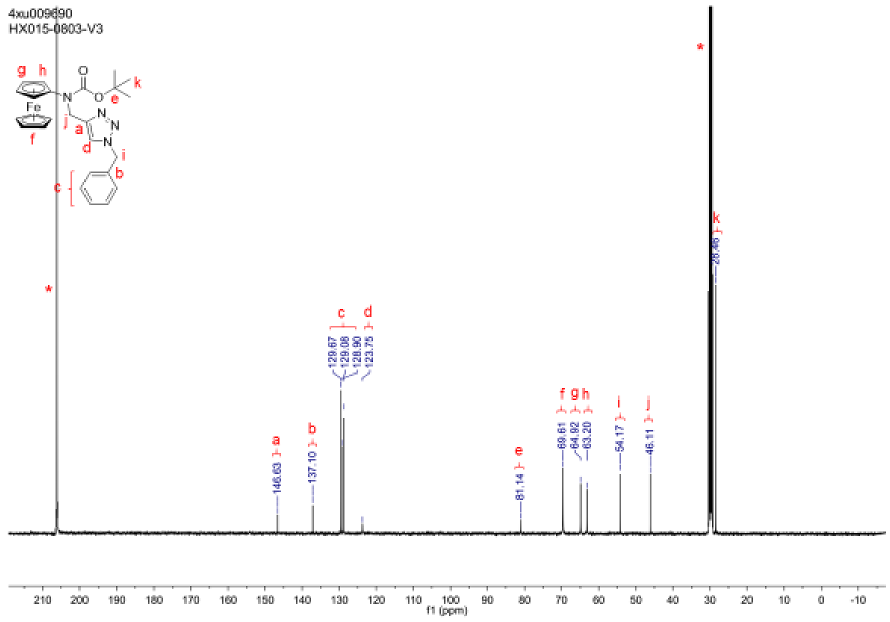

Figure **S32**. ^{13}C-NMR spectrum of intermediate **S9** in acetone-d$_6$. NMR solvent residual peaks are marked with *.

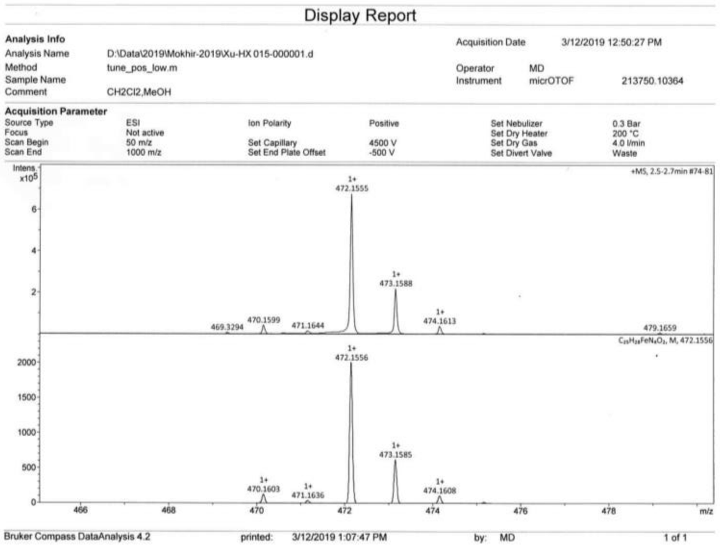

Figure **S33**. High resolution ESI-TOF mass spectrum of intermediate **S9**: upper plot – experimental spectrum; bottom plot – theoretical spectrum.

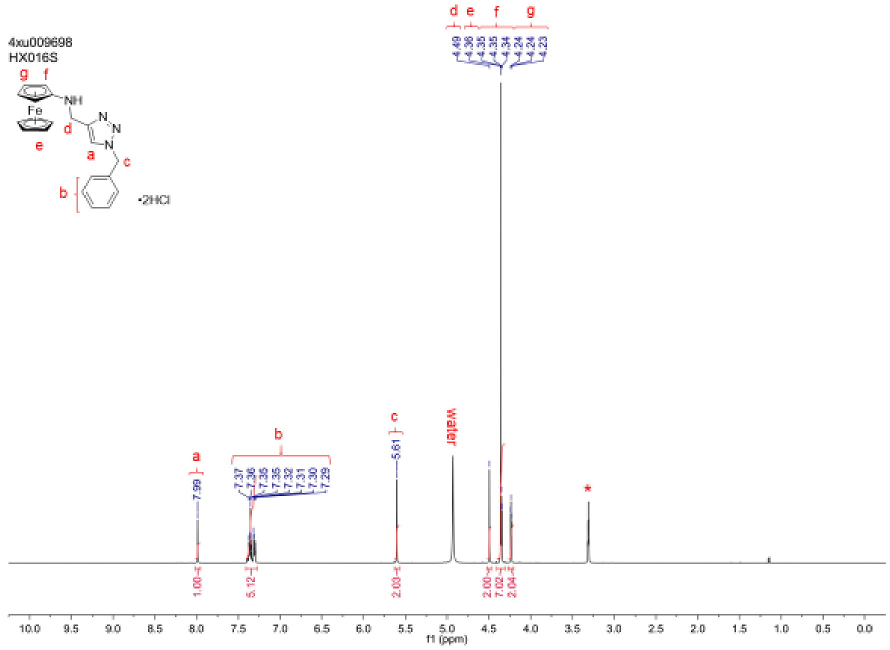

Figure **S34**. ^{1}H-NMR spectrum of HCl salt of drug **3** (**3***2HCl) in methanol-d$_4$. NMR solvent residual peaks are marked with *.

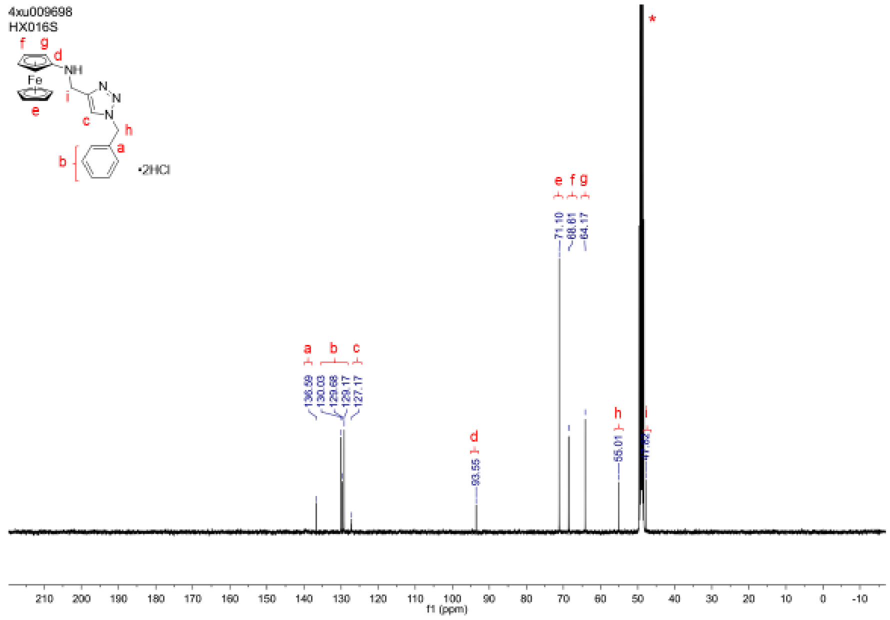

Figure **S35**. ^{13}C-NMR spectrum of HCl salt of drug **3** (**3***2HCl) in methanol-d$_4$. NMR solvent residual peaks are marked with *.

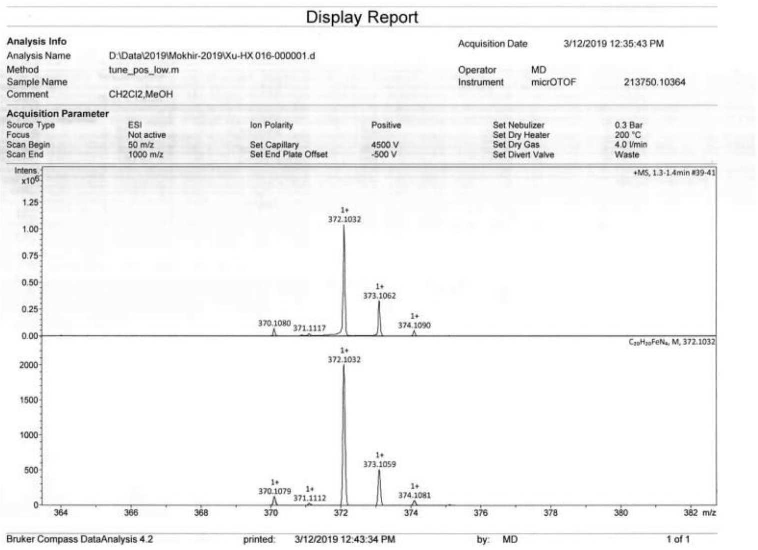

Figure **S36**. High resolution ESI-TOF mass spectrum of HCl salt of drug **3** (**3***2HCl): upper plot – experimental spectrum; bottom plot – theoretical spectrum.

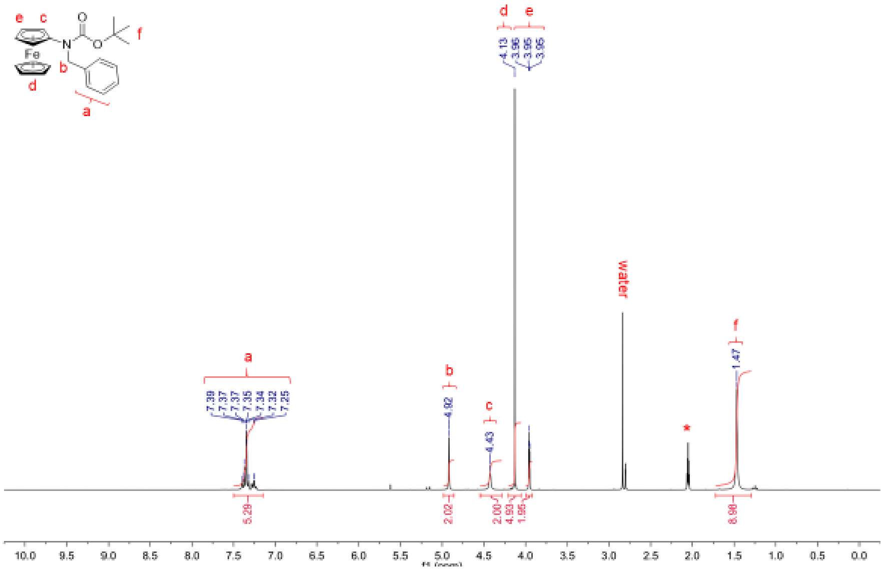

Figure **S37**. ^{1}H-NMR spectrum of intermediate **S10** in acetone-d6. NMR solvent residual peaks are marked with *.

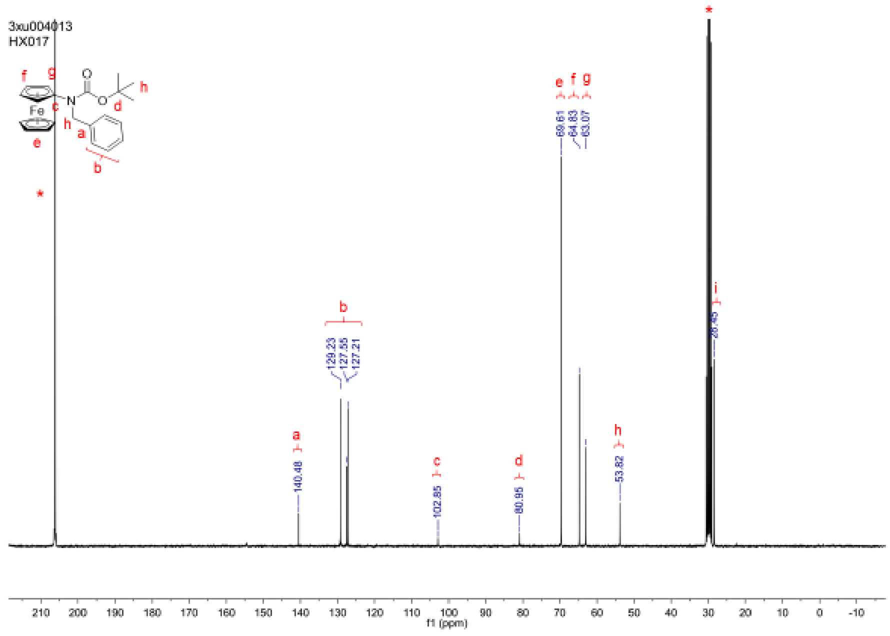

Figure **S38**. ^{13}C-NMR spectrum of intermediate **S10** in acetone-d_6. NMR solvent residual peaks are marked with *.

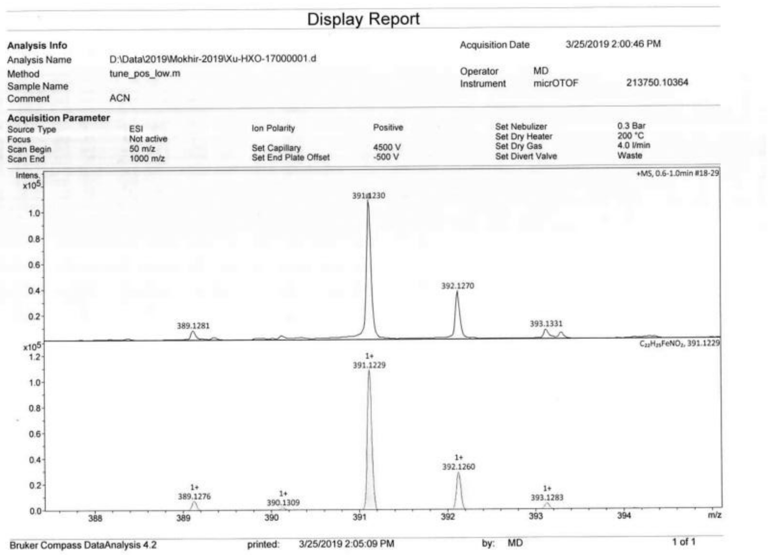

Figure **S39**. High resolution ESI-TOF mass spectrum of intermediate **S10**: upper plot – experimental spectrum; bottom plot – theoretical spectrum.

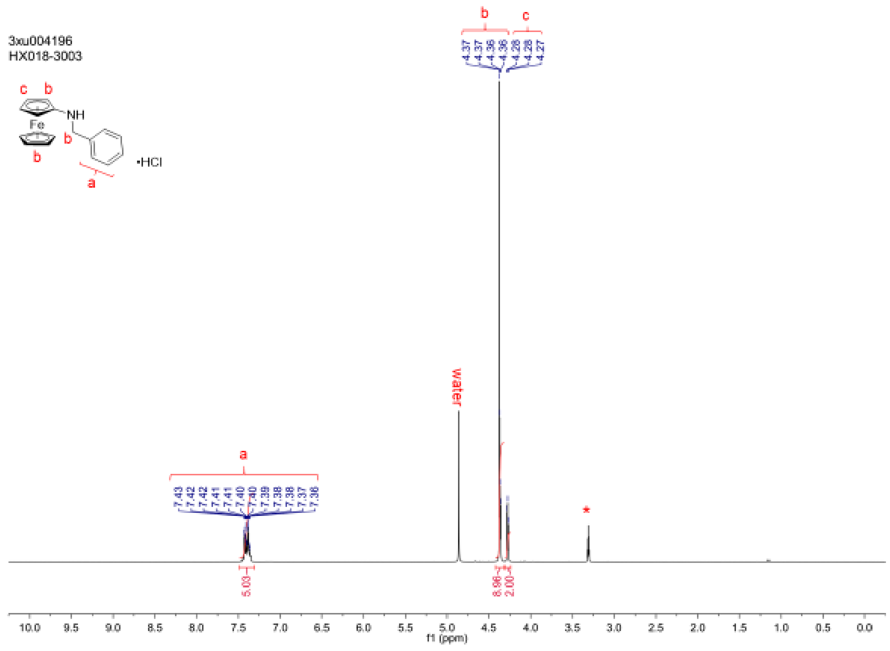

Figure **S40**. ^{1}H-NMR spectrum of HCl salt of drug **4** (**4***HCl) in methanol-d$_4$. NMR solvent residual peaks are marked *.

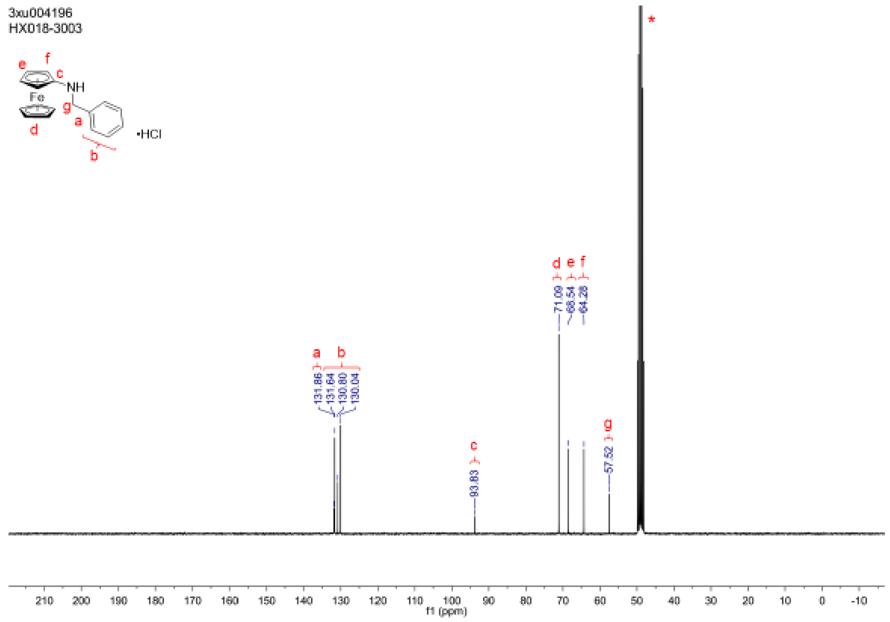

Figure **S41**. ^{13}C-NMR spectrum of HCl salt of drug **4** (**4***HCl) in methanol-d$_4$. NMR solvent residual peaks are marked with *.

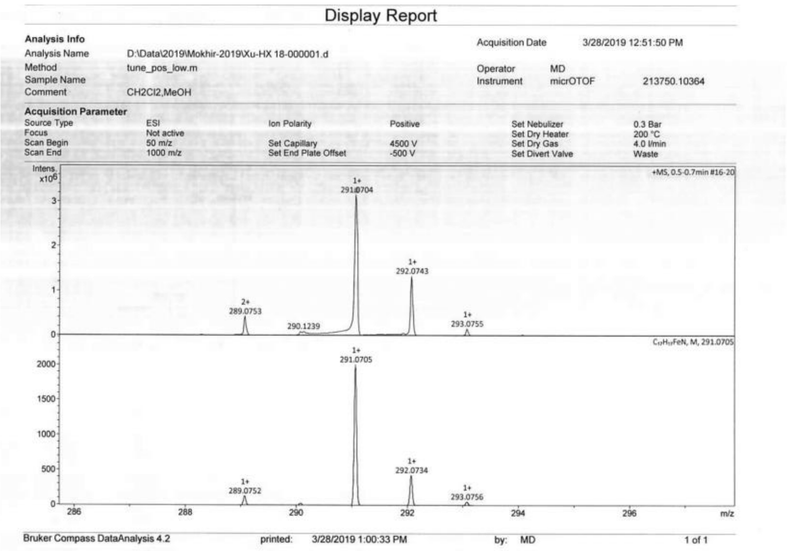

Figure **S42**. High resolution ESI-TOF mass spectrum of HCl salt of drug **4** (**4***HCl): upper plot – experimental spectrum; bottom plot – theoretical spectrum.

Determination of crystal structure of prodrug 2a

Crystals of prodrug **2a** were obtained by its re-crystallization from acetone (Figure 1). All details of crystal structure determination were deposited to Cambridge Structural Database (CCDC) under deposition number 1958761.

Evaluation of hydrolysis of prodrugs in the aqueous neutral solution by using HPLC-UV-MS

Stock solutions of prodrugs (**2a-2d**, 3 mM) in DMSO were first prepared. Then they were diluted 100-fold (final concentration: 30 µM) with CH_3CN/TEAA (triethylammonium acetate buffer - pH 7.0, 1.5 mM, 3/7, v/v). As a control pure DMSO was diluted in the same way. This probe was used to acquire background HPLC traces. After the probes (20 µL) of these solutions were incubated for 0 or 2 h at 37 ^{o}C, they were injected to HPLC coupled to UV-visible detector (detection of absorbance at 254 nm) and electrospray mass spectrometric detector (detection of positively charged ions in the m/z range of 300-1200) and the analytes were separated by using gradients of CH_3CN (containing formic acid, 0.1 %, v/v) in water (containing ammonium formate, 10 mM and formic acid, 0.1 %, v/v) from 15% to 95%. (Figure S43).

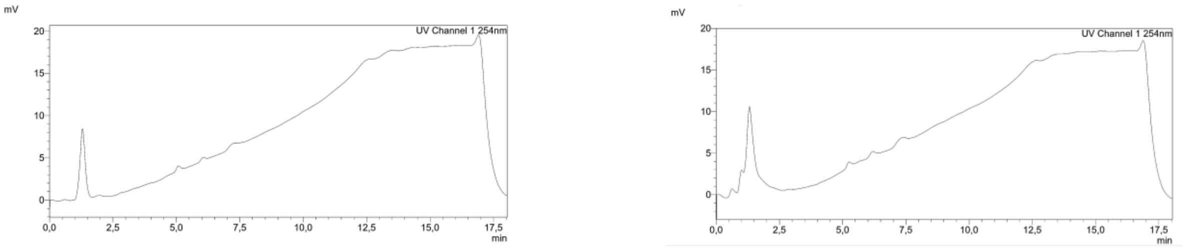

Control probe lacking any prodrug (left plot - 0 h, right plot - 2 h)

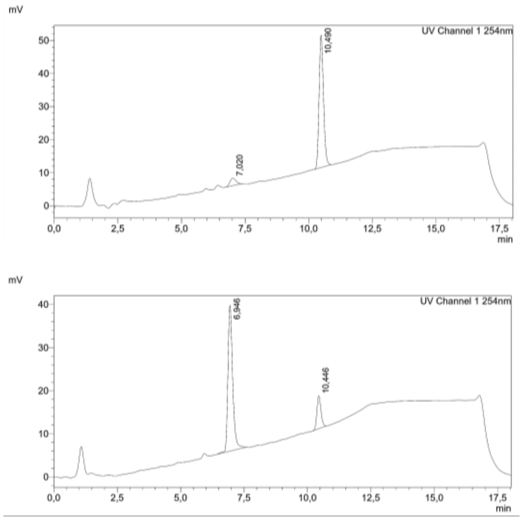

2a (upper plot - 0 h, lower plot - 2 h)

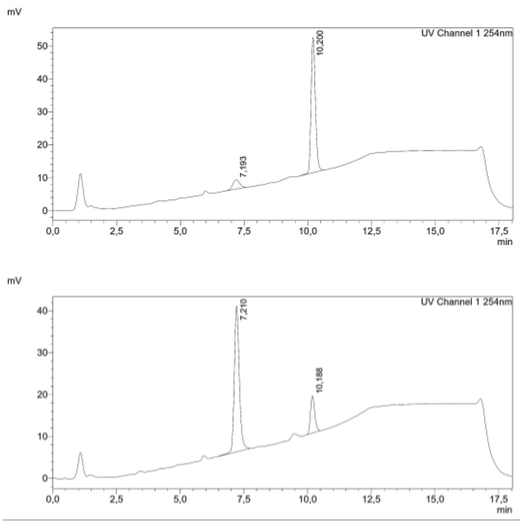

2c (upper- 0 h, lower - 2 h)

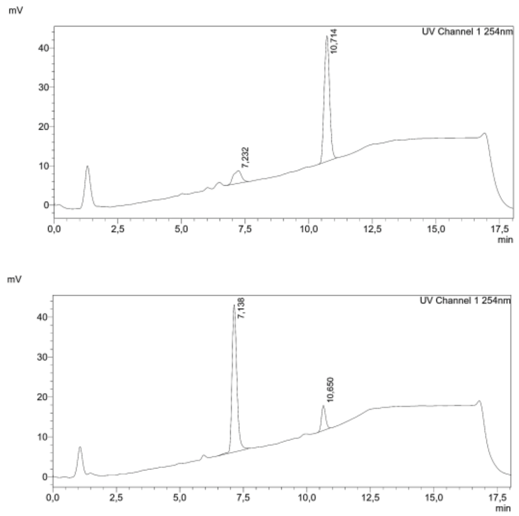

2b (upper - 0 h, lower- 2 h)

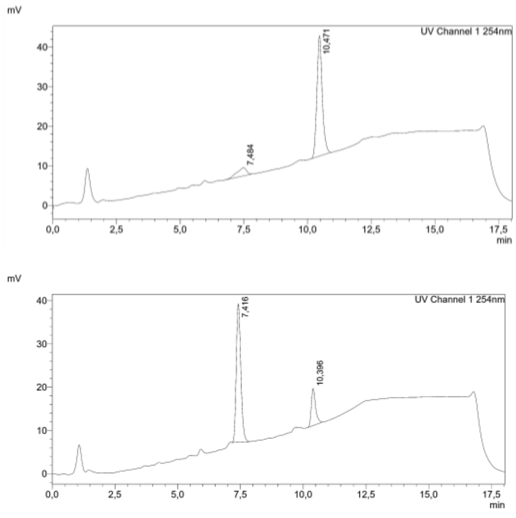

2d (upper - 0 h, lower - 2 h)

Figure **S43**. HPLC profiles of prodrugs **2a-2d** (30 µM) and the control probe incubated in CH_3CN/triethylammonium acetate buffer - pH 7.0, 1.5 mM, 3/7, v/v for 0 and 2 h.

Determination of n-octanol/water partition coefficients (LogP)

Solutions of references and new prodrugs were prepared by dissolving them in the mixture of dichloromethane/CH_3OH at proper concentrations. Commercially available reverse phase (RP) plates for thin layer chromatography (TLC, Macherey-Nagel, Germany, Alugram Aluminiumfolien RP-18 W/UV_{254}, stationary phase thickness: 0.15 mm) were used. The plates were manually spotted with freshly prepared solutions of the analytes at 10 mm distance from the edge of the plate. As the eluent mixture acetonitrile/aqueous 3-(N-morpholino)propanesulfonic acid (MOPS) buffer (100 mM, pH 7.4) (6/4, v/v) was used. The solvent migration distance was around 4.5 cm. The plates were visually inspected under the UV-light (254 nm) and each zone was clearly marked and its distance was manually measured. All measurements were done in at least triplicate and average values were used in further calculations. All experiments were performed at ambient temperature (22 ± 2 ^oC). A set of reference compounds was used, for which logP-values were previously reported: perylene (6.25), anthracene (4.50), benzophenone (3.18), phenylboric acid (1.59).[S1,S2] A calibration plot of R_f versus logP, which was obtained based on these data, was used to determine logP values of new prodrugs (Table 1).

Solubility of drugs in aqueous buffers

Observing prodrug precipitation by using bright field optical microscopy

Roswell Park Memorial Institute (RPMI) 1640 medium supplemented with FBS (5 %), L-glutamine (1 %), and penicillin/streptomycin (1 %) was filled into 96-well microtiter plates (100 μL for each well) and compounds in DMSO stock solution (1 μL) were added. Each well was monitored by using bright field optical microscopy both immediately after preparing these solutions/suspensions and after 48 h incubation at 37 °C in the chamber filled with CO_2 (5 %). The data obtained are provided in Figure S44.

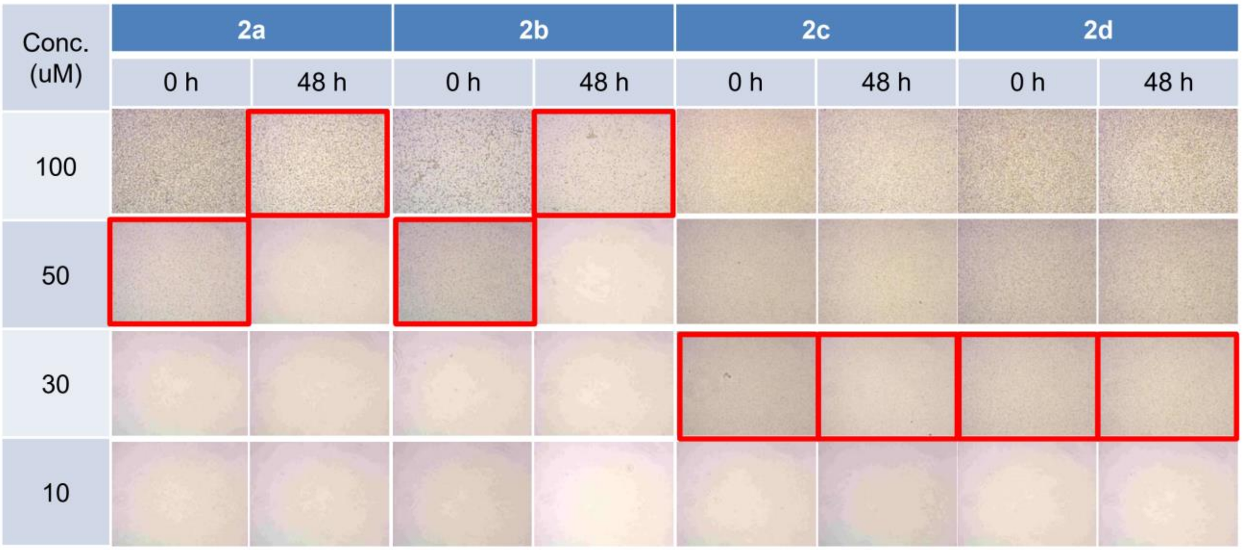

Figure **S44.** Microscopic images (Objective 40x/0.5 Ph0, Eyepiece WF 10x/20 Br. foc.) of solutions/suspensions of prodrugs **2a**-**2d** in RPMI 1640 medium taken either immediately (labelled “0 h”) or 48 h after mixing all components (labelled “48 h”). The wells containing the lowest concentration of the prodrug within data columns, where the precipitate was observed, are labelled with red rectangles.

Detection of oligomers in solutions of prodrugs by using dynamic light scattering

Compounds at different concentration (10 µL) were added to autoclaved microtubes (Eppendorf® microtubes 3810X with lid, polypropylene, 1.5 mL) containing 1 mL cell culture RPMI 1640 medium (the same medium was used in the MTT assay with A2780 cells, 48 h incubation with the drugs). The microtubes were vortexed for around 5 seconds and put into the cell incubator (Galaxy 170S, New Brunswick®). They were allowed standing at 37 °C in the chamber filled with CO_2 (5%) for 48 h before measurement. Then the samples were transferred to the disposable measuring cuvettes for dynamic light scattering (BRAND® macro-cuvette, polystyrene, 2.5 mL). Each sample was measured 3 times at 25 ^{o}C (Figure S45).

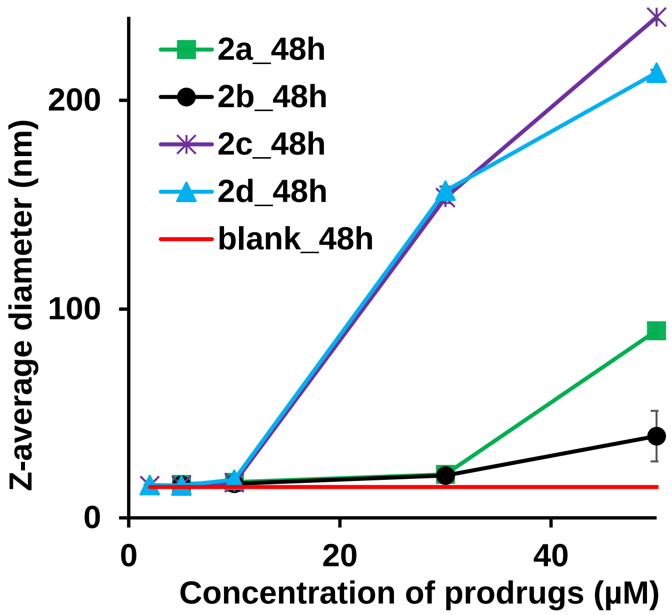

Figure **S45.** Correlation of mean particle size (Z-average diameter) and concentration of prodrugs compounds in RPMI 1640 medium. No prodrug was added to the "blank" sample. The DLS measurement was conducted 48h after preparation of prodrug solutions/suspensions.

Activation of prodrug 2c in the presence of H_2O_2

A freshly prepared solution of **2c** (10 µL, 2.5 mM in DMSO) was added to acetonitrile (CH_3CN, 100 µL). The resulting solution was mixed with water (897 µL) followed by the addition of either H_2O_2 (2.5 µL, 30% (w/w) in H_2O) or water (2.5 µL, negative control). These solutions were kept at 22 °C for 5 minutes. Then they were diluted with CH_3CN (1 mL) and analyzed by high resolution ESI-TOF mass spectrometry (Figures S46-S48).

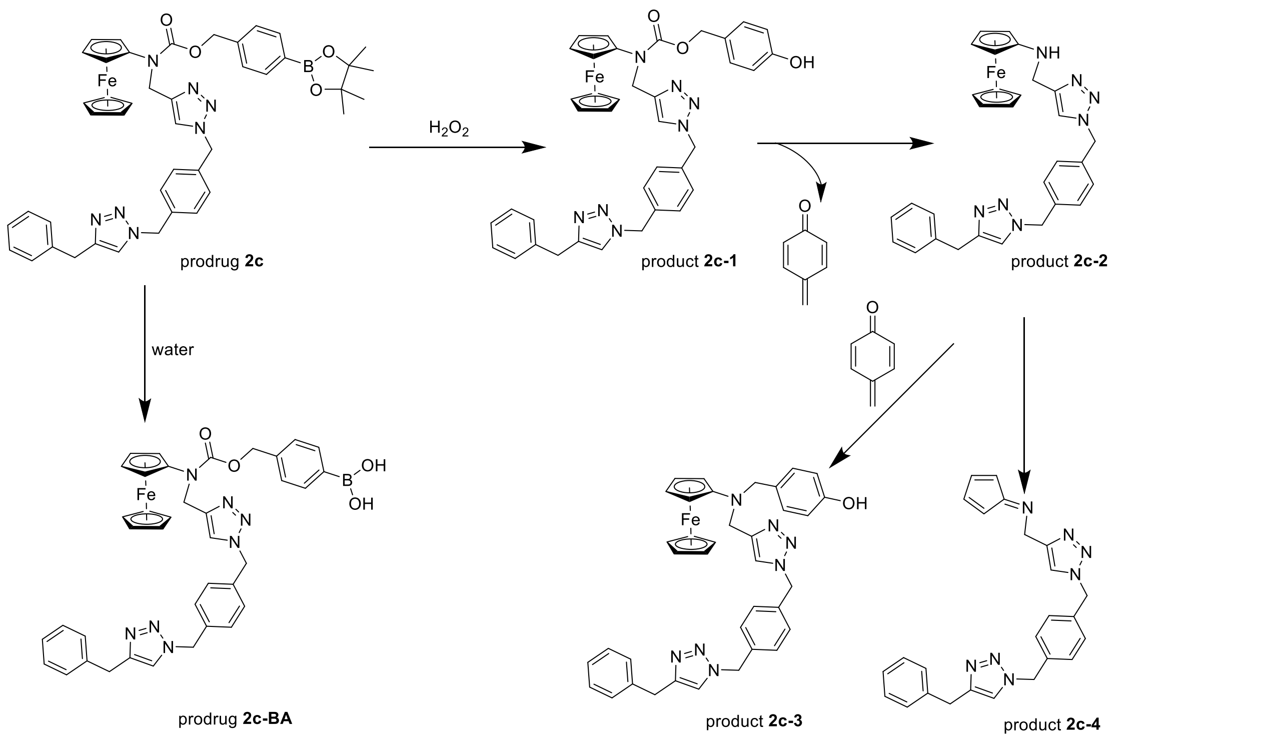

Figure **S46**. Structural formulas and exact masses of products identified in the mixtures of **2c** with/without H_2O_2 by using high resolution ESI-TOF mass spectrometry (Figures S50, S51).

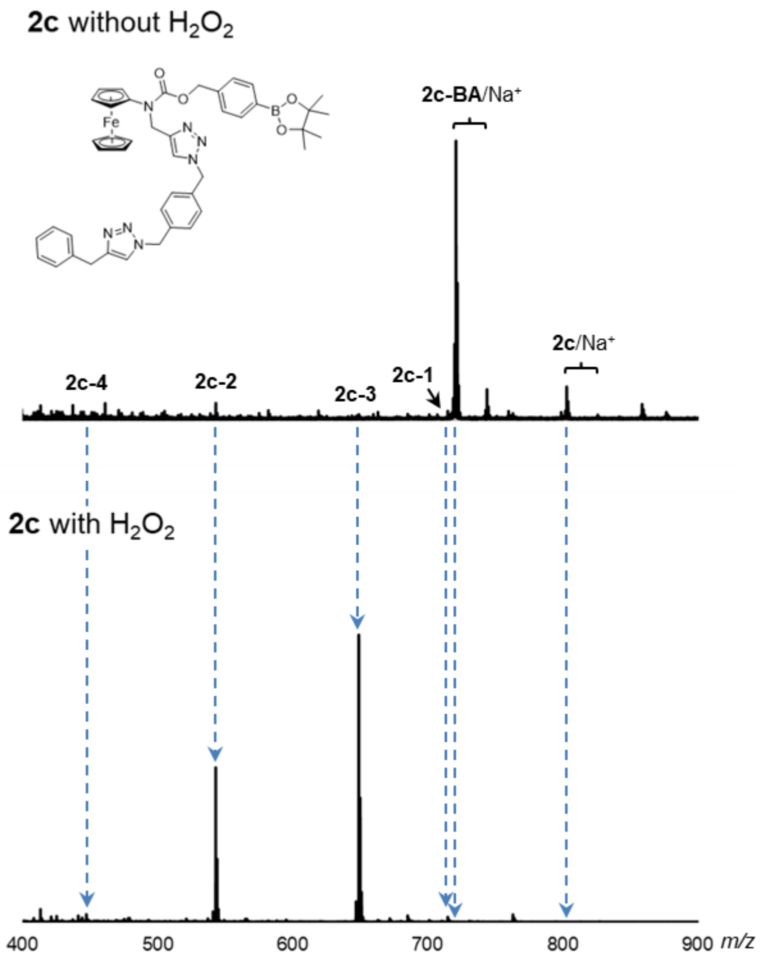

Figure **S47**. ESI-TOF mass spectra of prodrugs **2c** incubated for 5 minutes either in the absence of H_2O_2 or in its presence. Other experimental conditions are as described above. Expanded regions of the spectra shown in this figure are provided below in Figure S48.

[M+Na]+

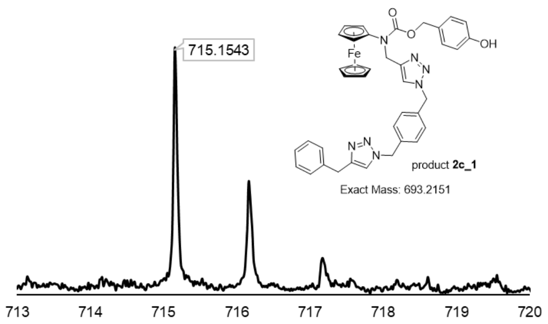

[M]+

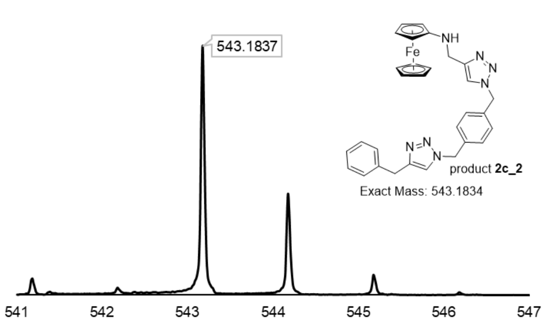

[M]+

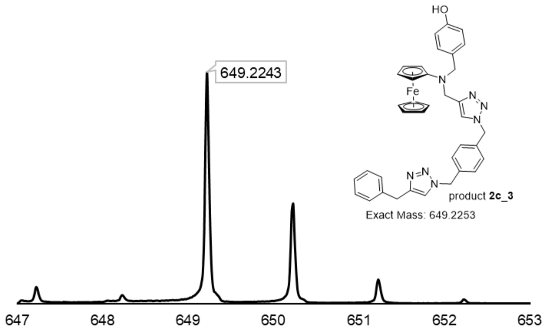

[M+Na]+

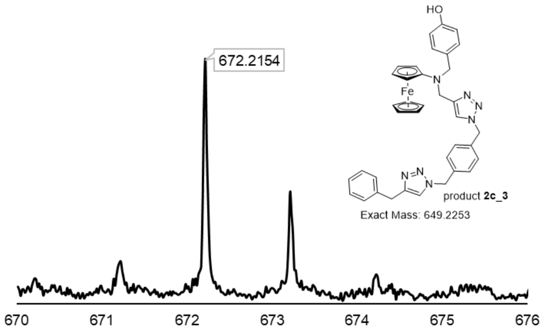

[M+Na]+

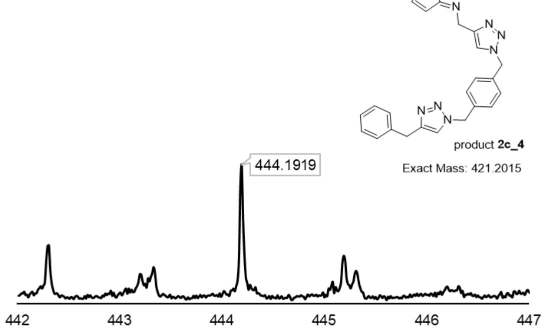

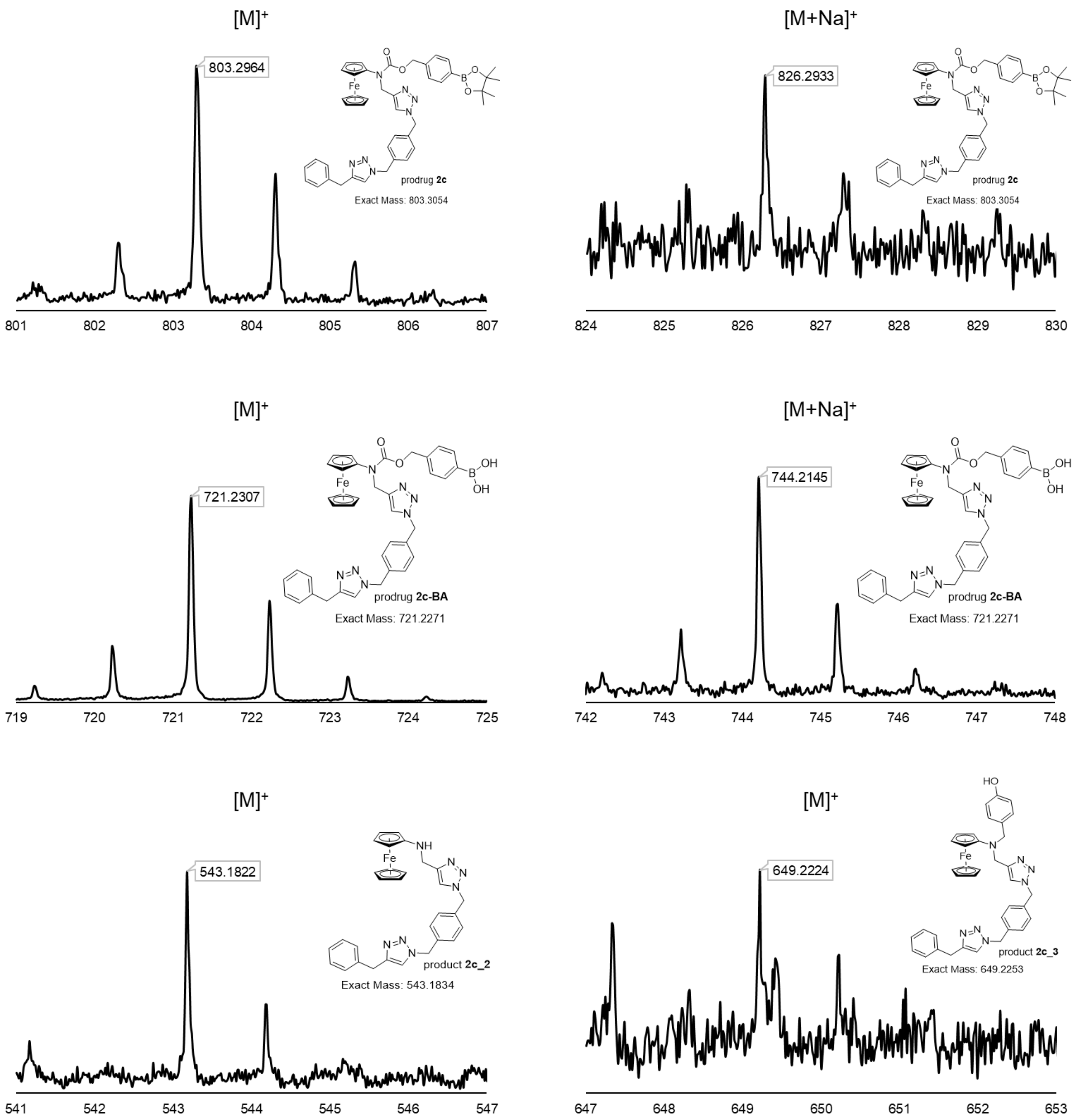

Figure **S48**. Expanded regions of the ESI-TOF mass spectra of mixtures of **2c** with H_2O_2 provided in Figure S47.

Prodrug-induced generation of ROS in cell free settings

2',7'-Dichlorodihydrofluorescein diacetate (DCFH-DA, 4.9 mg) was dissolved in DMF (100 µL) and mixed with aqueous NaOH (0.1 M, 900 µL). The resulting mixture was incubated for 30 min at 22 °C in the dark to obtain a stock solution of 2',7'-dichlorodihydrofluorescein (DCFH, 10 mM). Next, DCFH (1 µL) was added to the solution containing MOPS buffer (100 mM, pH 7.4), N,N,N'N'-ethylenediaminetetraacetic acid (EDTA, 10 mM) and glutathione (GSH, 5 mM) (1 mL, final concentration of DCFH 10 µM). The reaction mixture was heated to 37 °C and H_2O_2 (1 µL, final concentration 10 mM) was added. The reaction was monitored by fluorescence spectroscopy: λ_{ex} = 501 nm, λ_{em} = 523 nm. After around 5 min, prodrugs (1 mM in DMF, 10 µL) or positive control $FeSO_4$ (1 mM in water, 10 µL) were added, and the fluorescence monitoring was continued until the fluorescence signal growth was stalled after approximately 100 to 300 min. A representative kinetics of ROS release in the presence of prodrugs is given in Figure S49. The data are presented in Table 1 as relative acceleration of the rate of the fluorescence increase, where the rate of the fluorescence increase obtained in the presence of positive control $FeSO_4$ was set to 1 (reference).

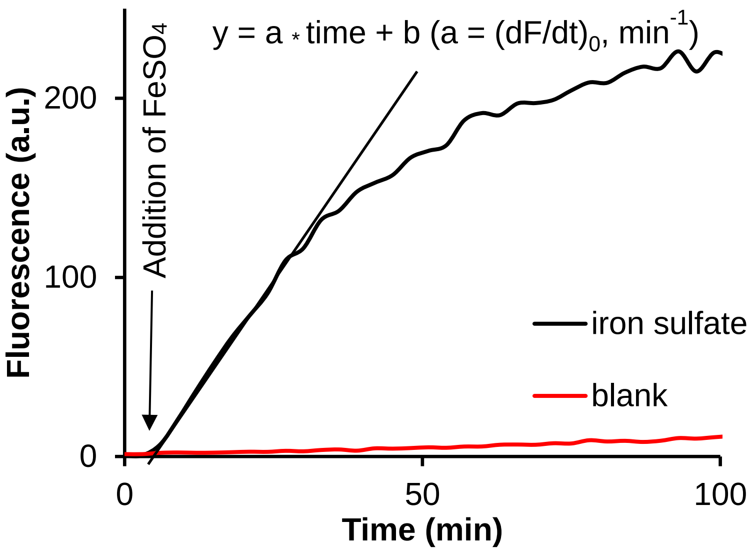

Figure **S49**. Increase of the fluorescence intensity (λ_{ex} = 501 nm, λ_{em} = 523 nm) upon oxidation of DCFH by H_2O_2 in the presence of $FeSO_4$. Experimental details are provided in the description above. The time point of the $FeSO_4$ addition is indicated with a black

colored arrow. After the initial lagging period, the oxidation reactions reach their maximum rates (indicated with a solid line for “iron sulfate” sample), which are provided in Table 1.

Accumulation of prodrugs in A2780 cells

A2780 cells (around 150 cells/µL, 40 mL) were seeded in T175 flasks (Greiner, Germany) in RPMI 1640 medium supplemented with 5% FBS, 1% L-glutamine, and 1% penicillin/streptomycin and incubated for 3-4 days at 37°C in the chamber filled with 5% CO_2. Next, a fresh portion of RPMI 1640 medium supplemented with 5% FBS, 1% L-glutamine, and 1% penicillin/streptomycin was added to replace old medium. Prodrugs (5 mM, 400 µL in DMSO, final concentration: 5 µM) or carrier (DMSO, 400 µL, final concentration: 1%. v/v) were added into the bottles and the cells were further incubated at 37°C for 4 h. The cells were washed with DPBS (2 x 10 mL) and trypsinated (trypsin/EDTA, 0.025%/0.01%, v/v, 2 mL). After the cells were harvested and centrifuged (5 min, 1000 rpm) in 50 mL falcon tube, the cell pellet was re-suspended in DPBS (1 mL), the suspension - transferred to 1.5 mL EP tube and centrifuged one more time (2 min, 2000 rpm). The obtained cell pellet was shaken with concentrated HNO_3 (65%, 100 µL) in an Eppendorf Thermomixer for 10 min at 95° C, 600 rpm. After cooling down, water (900 µL) was added. The insoluble components were separated by centrifugation (30 min, 13000 rpm) and the supernatant was analyzed by atomic emission spectrometry (AES) for the detection of B, Zn and Fe. The standard solutions for the AES were from Agilent or/and Bernd Kraft.

Table **S1**. Relative boron amount in A2780 cells treated with carrier, prodrugs **1** or **2c**.

	Relative boron amount[i]	Relative iron amount[i]
carrier	1.0 $\pm$ 0.5	1 $\pm$ 0
**1**	2.0 $\pm$ 0.1	1.4 $\pm$ 0.1
**2c**	15 $\pm$ 1	5.0 $\pm$ 0.1

[i] Zn amount was used as an internal standard.

Determination of viability of cells in the presence of prodrugs and controls

A2780 and BL-2 cells The medium was removed, and the cells were washed two times with DPBS, trypsinated, and resuspended in RPMI 1640 medium (A2780) supplemented with 5% FBS (for 24 h and 48 h assay) or 10% FBS (for 96 h assay), 1% L-glutamine, and 1% penicillin/streptomycin.

A2780 cells: This suspension was spread in the wells of a 96-well microtiter plate (25000 cells for A2780 per well per 100 μL) and left standing at 37 °C in the chamber filled with CO_2 (5%) for overnight. Stock solutions of prodrugs of different concentrations (1 μL, solvent dmso, final concentrations in wells were 2, 5, 10, 30, 50 μM) were added to the wells and incubated for specific periods of time. Four experiments were conducted for each concentration of the prodrug. Finally, 3-(4,5-dimethylthiazol-2-yl)-2,5-diphenyltetrazolium bromide (MTT, 20 μL of the solution prepared by dissolving MTT (5 mg) in DPBS buffer (1 mL)) was added to each well, incubated for 3 h, treated with sodium dodecyl sulfate (SDS) solution (90 μL, 10% solution in 0.01 M aqueous HCl), and incubated overnight. Afterward, the intensity of the absorbance at 590 nm was measured. MTT is converted in live cells to blue dye with the absorbance maximum λ_{max} at 590 nm. The absorbance at 690 nm was taken as a baseline value. The baseline corrected absorbance at 590 nm ($A_{(590\ nm)} - A_{(690\ nm)}$) was applied to calculate the relative number of viable cells. IC_{50} values were determined by fitting the experimental data expressing the number of viable cells (%, Y-axis) versus drug concentration (μM, X-axis) with a line curve and the X-axis value corresponding to 50% viability was considered as IC_{50} value according to the line curve (Figure S50, Table S2).

BL-2 cells: The cells were centrifuged, the medium was removed, and the cells were washed two times with DPBS and re-suspended in RPMI 1640 medium containing 5% (48 h incubation) or 10% FBS (96 h incubation), 1% L-glutamine, and 1% penicillin/ streptomycin. This suspension was spread in the wells of a 96-well microtiter plate (50000 cells per well per 100 μL). Other steps were identical to those was described in determination of viability of adherent cells (given above) (Table S3).

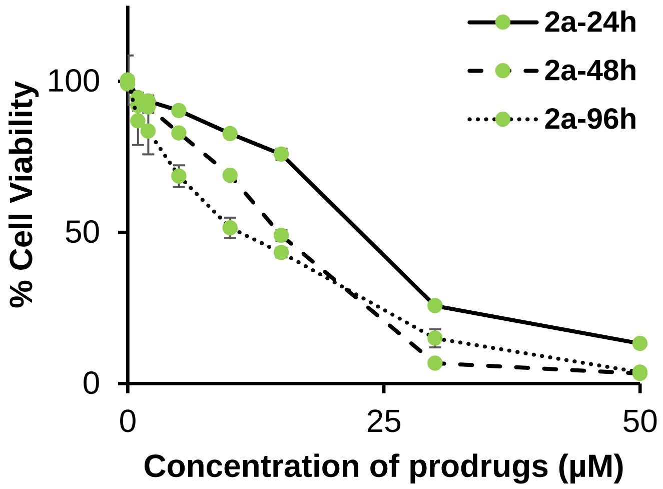
2a-24h
2a-48h
2a-96h
% Cell Viability
100
50
0
0
25
50
Concentration of prodrugs (μM)

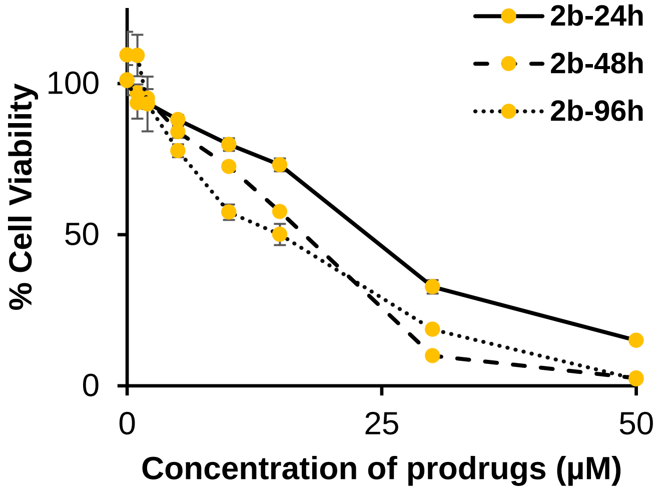
2b-24h
2b-48h
2b-96h
% Cell Viability
100
50
0
0
25
50
Concentration of prodrugs (μM)

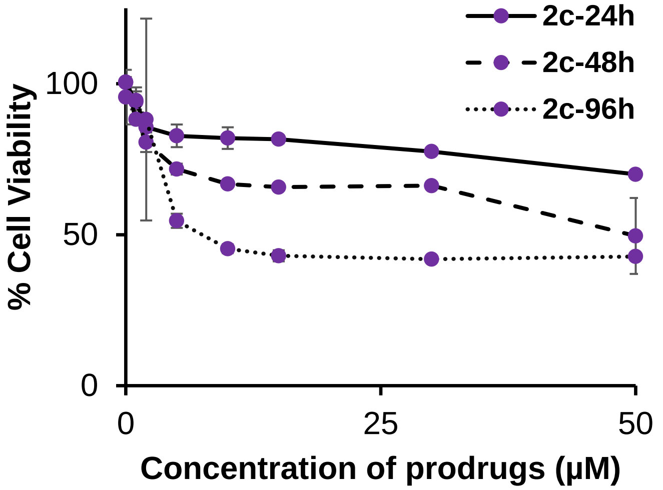
2c-24h
2c-48h
2c-96h
% Cell Viability
100
50
0
0
25
50
Concentration of prodrugs (μM)

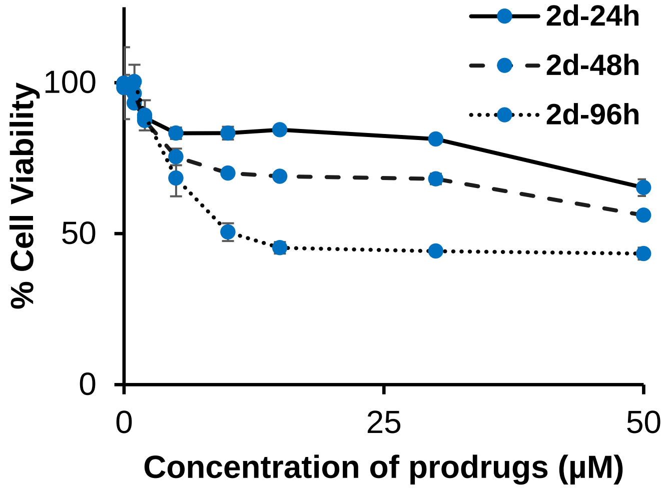

Figure **S50.** Effects of prodrugs **2a**-**2d** at different incubation times on viability of A2780 cells as determined by using the MTT assay described above. Incubation times are indicated next to prodrug codes.

Table **S2.** Effects of the prodrugs on the viability of A2780 cell lines (IC_{50} values) after different incubation times.

Compound	IC_{50} (μM)		
	24 h	48 h	96 h
**1**	-	32 ± 3	30 ± 5
**2a**	23.7 ± 0.6	16.2 ± 1.8	10.5 ± 0.7
**2b**	24.7 ± 0.6	18.5 ± 1.3	12.0 ± 3.0
**2c**	≥ 30	≥ 30	4.0 ± 0.5
**2d**	≥ 30	≥ 30	8.3 ± 1.5
**3**	-	>50	-
**4**	-	>50	-
**5b**	-	7 ± 2	7 ± 2
**5c**	10 ± 3	5 ± 2	5 ± 2
**6** (Fc-H, negative control)	>100	>100	>100
**7** (positive control)	3.5 ± 0.1	3.3 ± 0.3	2.2 ± 0.8

Table **S3.** Effects of the prodrugs on the viability of BL2 cell line (IC_{50} values) after 48 and 96h incubation.

Compound	IC_{50} (µM)	
	48 h	96 h
**1**	35 ± 2	16.9 ± 6.1
**2c**	40.1 ± 6.4	5.7 ± 2.7
**5b**	5 ± 2	5 ± 3
**5c**	5 ± 1	-
**6**	>100	>100
**7**	~1	2 ± 1

AsPC1 cells The cells (20.000/well) were seeded in a 12-well plate and incubated for 24 h. Afterwards, the medium was changed and the cells were further incubated for 72 h with medium containing different concentrations of compounds **2c** and **5b**. Viable cells were determined based on the morphological parameters by using flow cytometry (Table S4).

Table **S4.** Effects of the prodrugs on the viability of AsPC1 cell lines (IC_{50} values) after 72 h incubation.

Compound	IC_{50} (µM)
**2c**	7.1 ± 1.0
**5b**	6.7 ± 0.5

Jurkat cells The cells were counted in MUSE Cell Analyzer using MUSE® Count &Viability Assay Kit (Merck-Millipore, Billerica, MA, USA) and adjusted to a density of 1.0 x 10^5/ml in RPMI 1640 medium (with 10% FCS and 1% glutamine). 9.000 cells per well per 90 µL were seeded into 96 well culture plates. The 30 mM prodrug stock solutions in DMSO were diluted to 300 µM, 100 µM, 30 µM and 10 µM in cell culture medium and 10 µL of the dilutions was pipetted to the cells, receiving final test concentrations of 30 µM, 10 µM, 3 µM and 1 µM. Every concentration was tested in triplicates. Cells with corresponding amounts of DMSO served as negative controls. After 48 h cells were mixed and 25 µL of

the cell suspension was incubated with 200 µL of freshly prepared staining solution, consisting of AnnexinA5-Fitc (1 µL), 1,1′,3,3,3′,3′-hexamethylindodicarbocyanine iodide (DiIC1(5), 0.4 µL) (both from ThermoFisher Scientific, Waltham, MA, USA), propidium iodide (PI, 67 ng) and monobromobimane (MBB, 33 µM, both from Sigma-Aldrich, Taufkirchen, Germany) per 1 ml Ringer's solution (Fresenius Kabi AG, Bad Homburg, Germany). Cells were incubated for 20 min at 4°C and then analyzed in Gallios cytofluorometer™ (Beckman Coulter, Fullerton, CA, USA). Fitc and propidium iodide were excited at 488 nm; Fitc fluorescence was recorded on the FL1 sensor (525/38 nm band pass, BP) and propidium iodide fluorescence was detected on FL3 sensor (620/30 nm BP), DiIC1(5) fluorescence was excited at 638 nm and detected on FL6 sensor at 675/20 nm BP, and monobromobimane fluorescence was excited at 405 nm and detected on FL9 sensor at 430/40 nm BP. To eliminate any fluorescence bleed-through, electronic compensation was used. Data were analyzed employing Kaluza™ software Version 1.2 (Beckman Coulter, Fullerton, CA, USA) and processed in Microsoft Excel (Figures S51-S54, Table S5). Cells negative for Annexin A5-Fitc and propidium iodide were considered viable. IC_{50} values were determined by fitting the % values of Ax-PI- cells versus drug concentration with a sigmoidal curve using GraphPad Prism software. Reduced glutathione (an intracellular thiol) can be detected by monobromobimane, a dye that becomes fluorescent after non-enzymatic binding to thiols.[S3] For analysis of amount of mitochondrial membrane potential or reduced glutathione the mean fluorescence index (MFI) was determined from viable cells by gating on Ax-PI- events.

Table **S5.** Effects of the prodrugs on the viability of Jurkat cell lines (IC_{50} values) after 48 h incubation.

Compound	IC_{50} (µM)
**1**	30 ± 4
**2c**	5 ± 2
**5b**	7.2 ± 0.1
**5c**	20 ± 2
**6**	>100

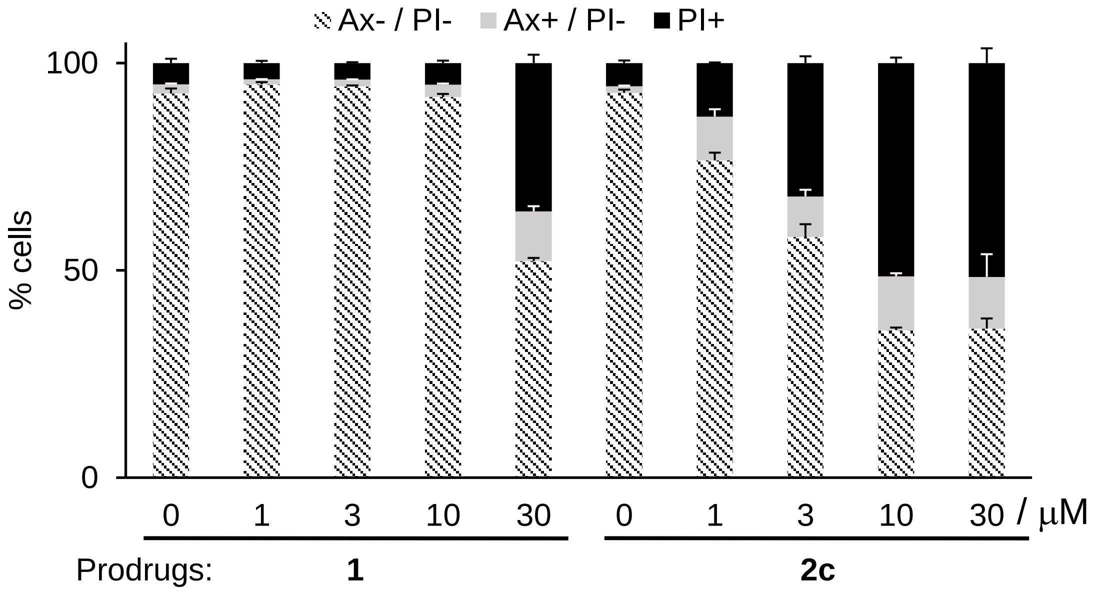

Figure **S51**. The mechanism of Jurkat cell death in the presence of prodrugs **1** and **2c** (incubation time: 48 h). Ax-/PI-: viable cells; Ax+/PI-: apoptotic cells; PI+: necrotic cells. Detailed experimental description is provided above.

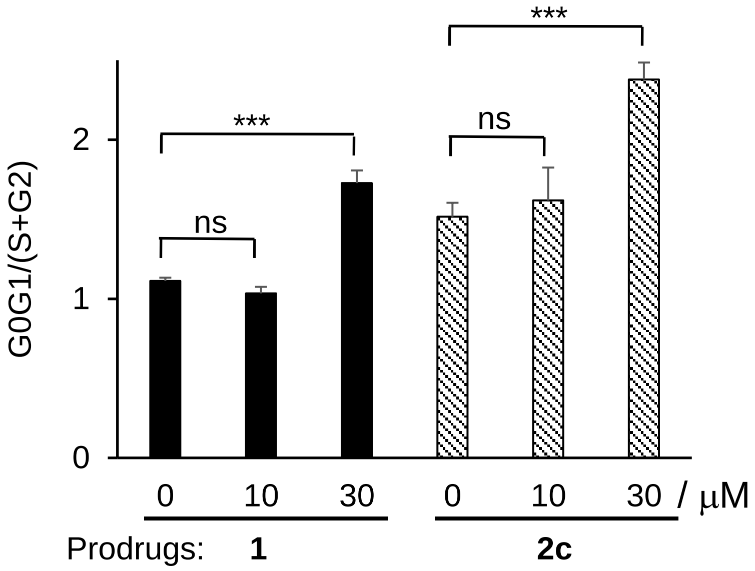

Figure **S52**. Effect of prodrugs **1** and **2c** (48 h incubation) on Jurkat cell cycle. ***: p< 0.001; ns: non-significant (Student's t test). Detailed experimental description is provided above.

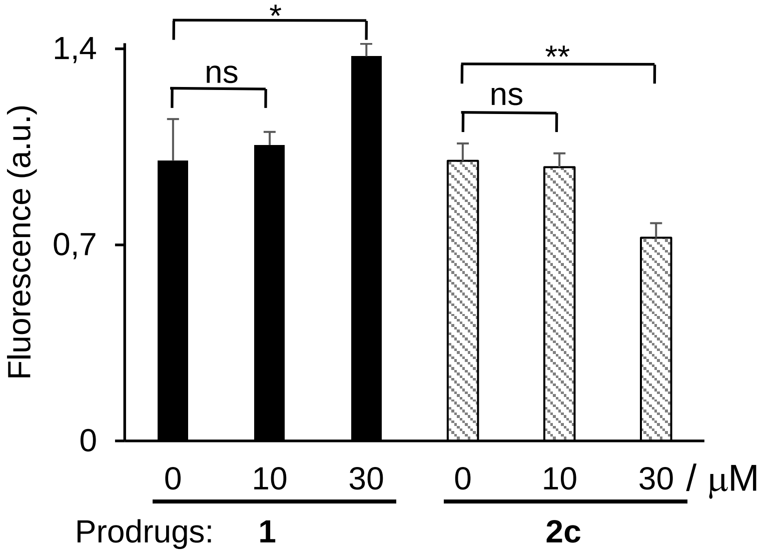

Figure **S53**. Mitochondrial membrane potential (MMP) of Jurkat cells after their incubation for 48 h with prodrugs **1** and **2c**. *: p< 0.05; **: p< 0.01; ns: non-significant (Student's t test). Detailed experimental description is provided above.

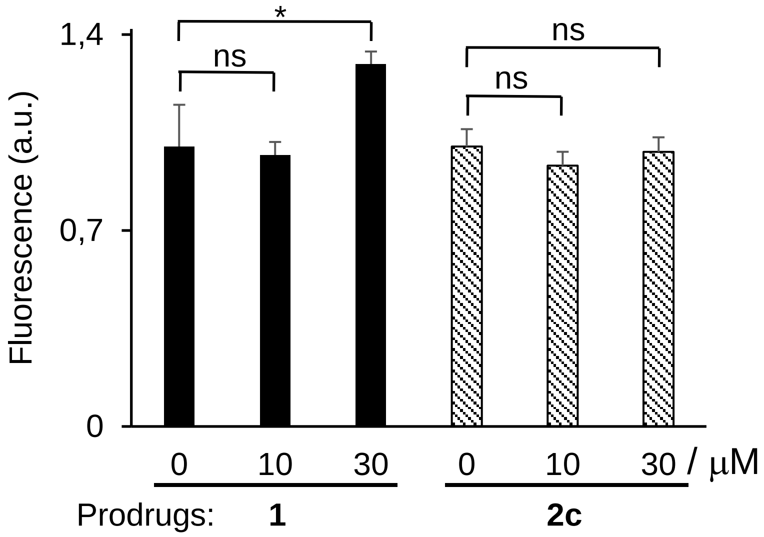

Figure **S54**. Effect of prodrugs **1** and **2c** (48 h incubation) on the intracellular amount of GSH in Jurkat cells. *: $p< 0.05$; ns: non-significant (Student's t test). Detailed experimental description is provided above.

Study of the mechanism of death of A2780 cells induced by prodrug **2c** and controls

A2780 cells were seeded into 96 well culture plates at the same cell concentration and in the same medium as applied for the above described MTT assay (48 h incubation with the prodrugs). The next day, stock solutions of prodrugs of different concentrations (1 µL, solvent DMSO, final concentrations in wells were 5, 10 and 30 µM) or carrier only (DMSO, 1 µL, final concentration 1 %, v/v) were added to the wells and incubated in the chamber filled with CO_2 (5%). After 48 h supernatants were transferred into 1.5 mL Eppendorf tubes and 100 µL of DPBS was added to transfer supernatant residue into tube. Trypsin (0.25%, 50 µL) was then added to well to detach the cells. All supernatants in Eppendorf tubes were combined back into wells. After gently pipetting up and down, 50 µL of cell solutions was transferred into a new Eppendorf tube and incubated with 200 µL of staining solution, consisting of Annexin A5-Fitc (5 µL) and propidium iodide (PI, 330 ng) per 1 mL buffer (conc. 1X). The cells were incubated for 20 min at 22°C in dark and then put on ice followed by analysis using flow cytometry (λ_{ex} = 488 nm, λ_{em} = 530 nm for Annexin A5-

Fitc and λ_{em} = 664 nm for PI). The cells negative for Annexin (Ax-) and propidium iodide (PI-) were considered viable. The cells positive for Annexin (Ax+) and negative for PI (PI-) were considered apoptotic. The cells positive for PI (PI+) were considered necrotic. (N=3, Figure S55).

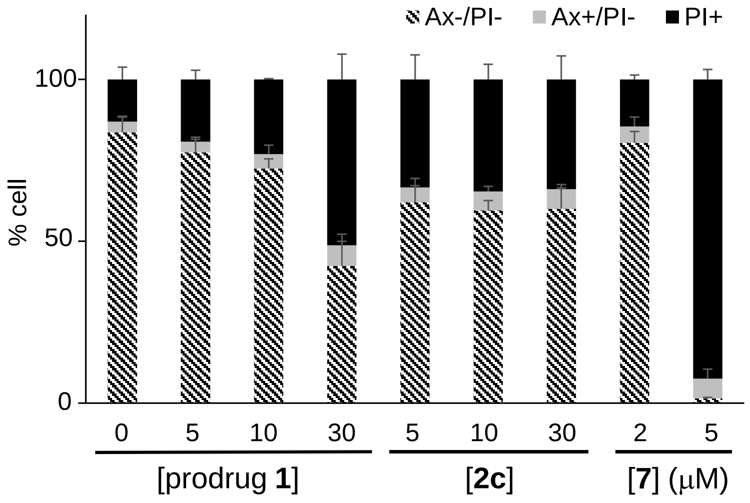

Figure **S55**. Effects of the prodrugs on the viability of A2780 cell lines after 48 h incubation. Viable cells are indicated as Ax-/PI-, apoptotic as Ax+/PI- and necrotic as PI+.

Effect of prodrugs and controls on cellular organelles in A2780 cells: lysosomes (LY), endoplasmic reticulum (ER), Golgi (G) and mitochondria (Mit) (Figures 3, S56)

A2780 cells were seeded in RPMI 1640 medium containing 5% FBS, 1% L-glutamine and 1% penicillin/streptomycin in a 24-well microtiter plate (total volume 0.5 mL) at a density of 200 cells/µL one day before the experiment. On the day of the experiment, prodrugs ("LY/ER/G"-experiments: stock solution - 3 mM, 5 µL, final concentration: 30 µM; "Mit"-experiment: stock solutions - 0.5, 1 and 3 mM, 5 µL, final concentrations: 5, 10, 30 µM) or carrier only (DMSO, 1%, v/v) were added to cells and incubated for 1, 4 and 24 h in a dark chamber filled with CO_2 (5 %) at 37 °C. The cells were washed twice with DPBS buffer and the medium was replaced with HBSS (Hanks' Balanced Salt solution, 500 µL). Stock solutions of different dyes (5 µL) were added: concentrations of the stock solutions were 4 mg/L for Acridine orange (AO), 10 mg/L for Rhodamine 123 (R123), 1 µM for ER-Tracker-Green (ERG) and 10 µM for Golgi-Staining-Green (GO). AO, R123, ERG were

dissolved in DMSO, whereas GO was dissolved in H_2O. Final concentrations of solutions were 40 µg/L for AO, 100 µg/L for R123, 0.01 µM for ERG and 0.1 µM for GO. The cells were incubated in the dark chamber filled with CO_2 (5%) at 37 °C for 20 min. Afterwards the supernatant was removed, the cells were washed with DPBS buffer twice and detached from the culture dish using trypsin/EDTA (0.025%/0.01%, v/v, 200 µL). Next, the cells were re-suspended in fresh medium. Finally, the fluorescence of live cells (the number of total events was 5000, λ_{ex} = 488 nm, λ_{em} = 530 nm) in the suspensions was determined by using flow cytometry (N=3, Figure 3, S56).

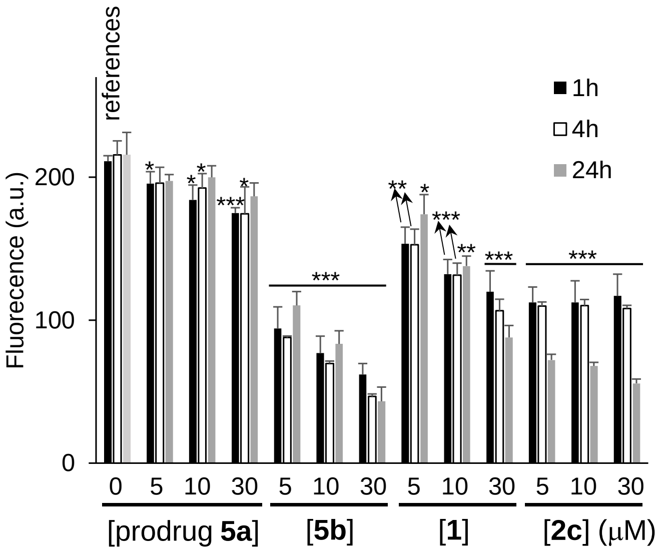

Figure **S56**. Effects of the prodrugs on mitochondria membrane potential (MMP) of A2780 cell lines after 1-48 h incubation. *: p< 0.05; **: p< 0.01; ***: p< 0.001, Student's t test. Non-labelled data are not significantly different (p≥ 0.05) from the corresponding reference (labelled on the plot).

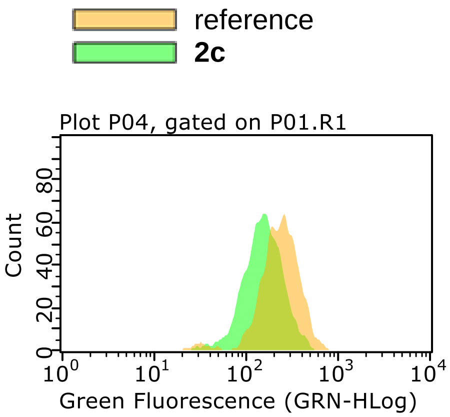

Figure 3 A

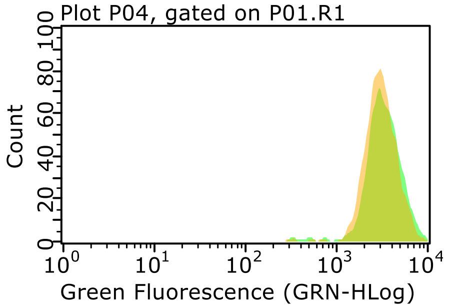

Figure 3 B

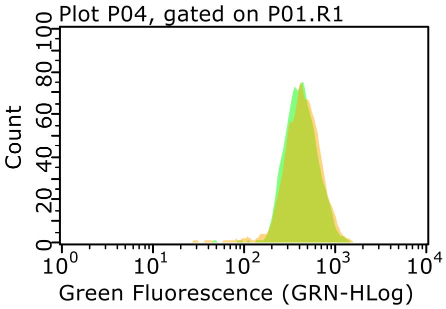

Figure 3 C

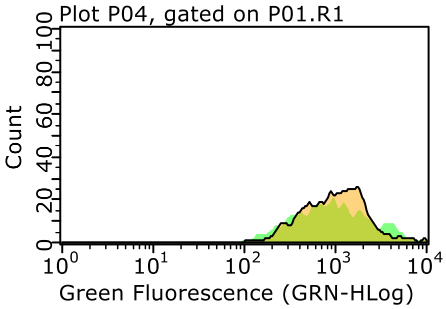

Figure 3 D

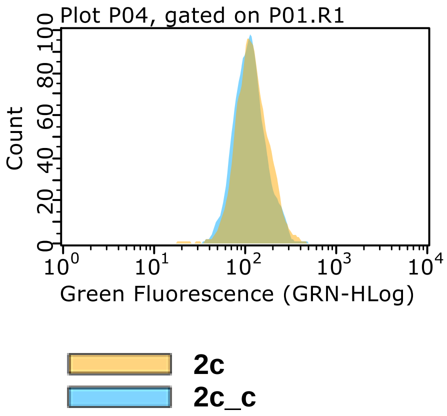

Figure 5 A

Figure **S57**. Representative flow cytometric histograms used for setting up Figures 3A-D (main text) and 5A.

Monitoring total intracellular ROS (tROS)

A2780 cells were seeded in RPMI 1640 medium containing 5% FBS, 1% L-glutamine and 1% penicillin/streptomycin in a 24-well microtiter plate (total volume 0.5 mL) at a density of 200 cells/µL one day before the experiment. On the day of the experiment, the cells were washed twice with DPBS buffer and the medium was replaced with HBSS (Hanks' Balanced Salt solution, 500 µL). CM-DCFH-DA (5-(6-)chloromethyl-2',7'-dichlorodihydrofluorescein diacetate, Thermo- Fisher) solution (0.1 mM in DMSO, 5 µL) was added (final concentration 1 µM) and the cells were incubated in the dark chamber filled with CO_2 (5%) at 37 °C for 15 min. Afterwards the supernatant was removed, the cells were washed with DPBS buffer and a fresh portion of RPMI 1640 medium containing 5% FBS, 1% L-glutamine and 1% penicillin/streptomycin (500 µL) was added followed by addition of prodrugs (5 µL of stock solutions in DMSO, final concentration of prodrugs 5, 10 and 30 µM) or carrier only (DMSO, 5 µL, final concentration 1 %, v/v) and incubation for 2 h in the dark chamber filled with CO_2 (5%) at 37 °C. Next, the cells were detached from the culture dish using trypsin/EDTA (0.025%/0.01%, v/v, 200 µL). The cells were re-suspended in RPMI 1640 medium containing 5% FBS, 1% L-glutamine and 1% penicillin/streptomycin (200 µL). Finally, the fluorescence of live cells (λ_{ex} = 488 nm, λ_{em} = 525 nm) in the suspensions was determined by using flow cytometry (Figure 4A, 5C). Intracellular ROS release in Jurkat cells was monitored analogously (Figure S58).

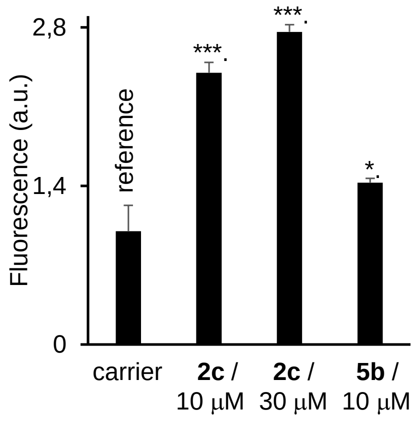

Figure **S58**. Effects of prodrugs **2c** and **5b** on the intracellular total ROS amount (tROS) in Jurkat cells. OY-axis “Fluorescence”: the increase of the mean fluorescence (λ_{ex} = 488

nm, λ_{em} = 525 nm) of (CM-DCFH-DA)-loaded Jurkat cells incubated with the prodrugs for 1 h relative to that of the cells incubated with the carrier only. *: p< 0.05; ***: p< 0.001 (Student's t test with respect to the "carrier" probe: indicated with "reference" on the plot).

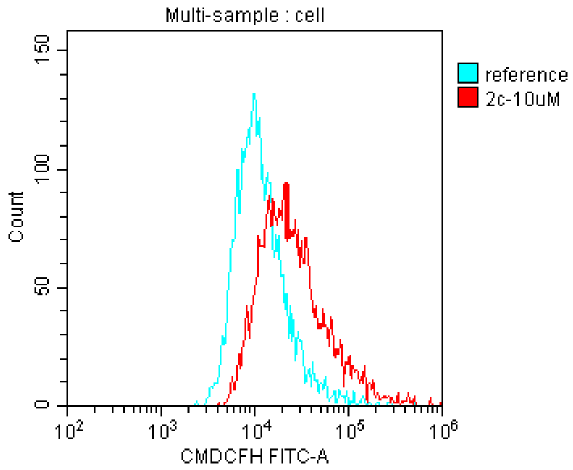

Figure 4 A

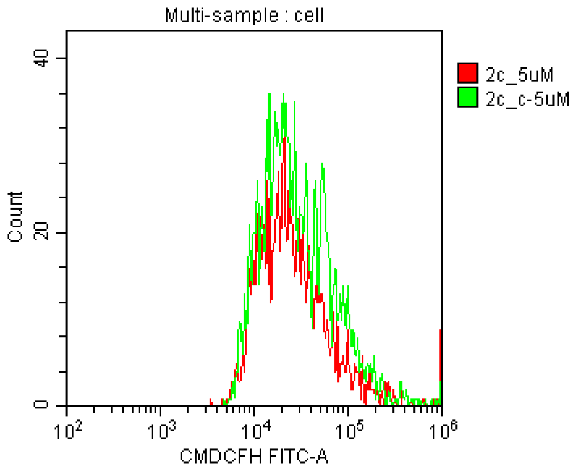

Figure 5 C

Figure **S59**. Representative flow cytometric histograms used for setting up Figures 4A and 5C.

Monitoring mitochondrial ROS (mROS)

A2780 cells were seeded in RPMI 1640 medium containing 5% FBS, 1% L-glutamine and 1% penicillin/streptomycin in a 96-well microtiter plate (total volume 0.1 mL) at a density of 250 cells/µL one day before the experiment. On the day of the experiment, the cells were washed with DPBS buffer and a fresh portion of RPMI 1640 medium containing 5% FBS, 1% L-glutamine and 1% penicillin/streptomycin (100 µL) was added followed by addition of compounds (1 µL of stock solutions in DMSO, final concentration of the prodrugs was 5, 10 µM and 30 µM) or carrier only (DMSO, 1 µL, final concentration 1 %, v/v) and incubation for 1 or 2 h in the dark chamber filled with CO_2 (5%) at 37 °C. After that, the cells were washed twice with DPBS buffer and the medium was replaced with HBSS (Hanks' Balanced Salt solution, 50 µL) containing MitoSOX (Red Mitochondrial Superoxide Indicator, Thermo-Fisher, Catalog number M36008, final concentration 5 µM obtained by adding 0.5 µL of the 0.5 mM stock solution of MitoSOX in DMSO to the HBSS)

and the cells were incubated in the dark chamber filled with CO_2 (5%) at 37 °C for 20 min. Next, the cells were detached from the culture dish using trypsin/EDTA (0.025%/0.01%, v/v, 50 µL). The cells were re-suspended in RPMI 1640 medium containing 5% FBS, 1% L-glutamine and 1% penicillin/streptomycin (100 µL). Finally, the fluorescence of live cells (λ_{ex} = 488 nm, λ_{em} = 578 nm) in the suspensions was determined by using flow cytometry (Figure 4B, 5D).

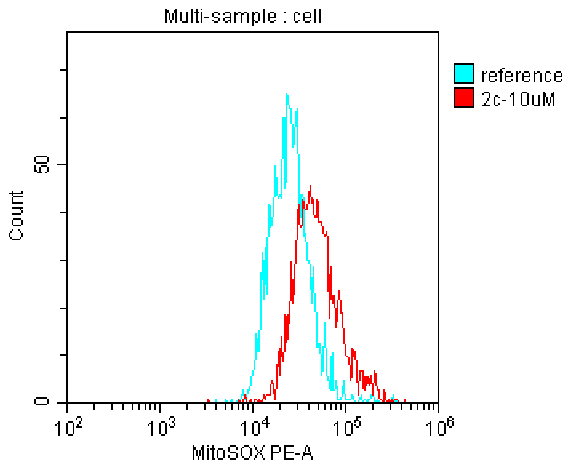

Figure 4 B

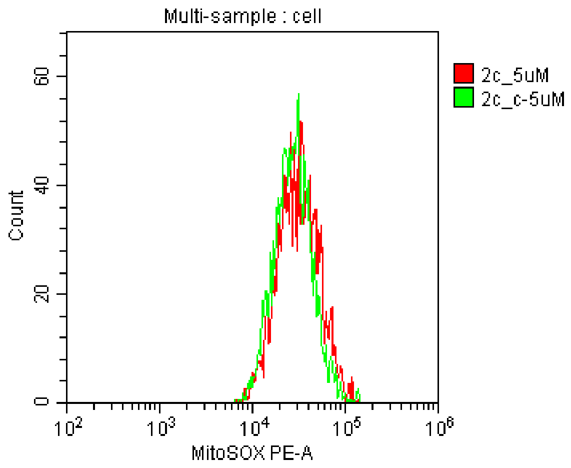

Figure 5 D

Figure **S60**. Representative flow cytometric histograms used for setting up Figures 4B and 5D.

Monitoring intracellular carbonyl derivatives

A2780 cells were seeded into 24 well culture plates at the same cell concentration and in the same medium as applied for the assay for monitoring tROS described above. The next day, stock solutions of prodrugs of different concentrations (5 µL of stock solutions in DMSO, final concentration of prodrugs 5 and 10 µM; control **7** 2 and 5 µM) or carrier only (DMSO, 5 µL, final concentration 1 %, v/v) were added to the wells and incubated in the chamber filled with CO_2 (5%). After 24 h, the cells were washed twice with DPBS buffer and the medium was replaced with HBSS (Hanks' Balanced Salt solution, 500 µL). 7-(Diethylamino)coumarin-3-carbohydrazide (DCCH) solution (8 mM in DMSO, 5 µL) was added (final concentration 80 µM) and the cells were incubated in the dark chamber filled

with CO_2 (5%) at 37 °C for 2 h. Next, the cells were detached from the culture dish using trypsin/EDTA (0.025%/0.01%, v/v, 200 µL). The cells were re-suspended in RPMI 1640 medium containing 5% FBS, 1% L-glutamine and 1% penicillin/streptomycin (200 µL). Finally, the fluorescence of live cells (λ_{ex} = 488 nm, λ_{em} = 530 nm) in the suspensions was determined by using flow cytometry (Figure 4C).

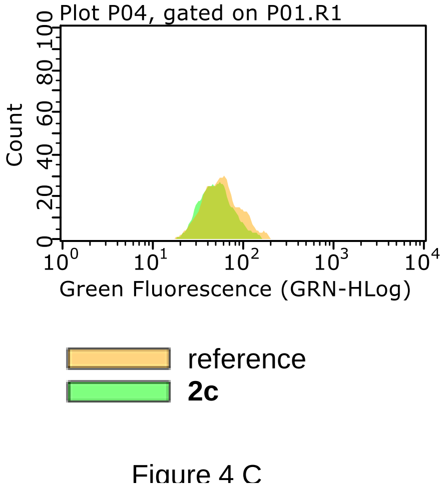

Figure **S61**. Representative flow cytometric histograms used for setting up Figure 4C.

Generation of nitric oxide in A2780 cells

A2780 cells were seeded into 96 well culture plates at the same cell concentration and in the same medium as applied for the assay for monitoring mROS described above. On the day of the experiment, the cells were washed twice with DPBS buffer and the medium was replaced with HBSS (100 µL). DAF-FM diacetate (4-Amino-5-Methylamino-2',7'-Difluorofluorescein Diacetate, Catalog number: D23844, Thermo-Fisher) solution (0.5 mM in DMSO, 1 µL) was added (final concentration 5 µM) and the cells were incubated in the dark chamber filled with CO_2 (5%) at 37 °C for 20 min. Afterwards the supernatant was removed, the cells were washed with DPBS buffer and a fresh portion of RPMI 1640 medium containing 5% FBS, 1% L-glutamine and 1% penicillin/streptomycin (100 µL) was added followed by addition of prodrug or carrier only (DMSO, 1 µL, final concentration 1 %, v/v) and incubation for 2 h in the dark chamber filled with CO_2 (5%) at 37 °C. Next, the cells were detached from the culture dish using trypsin/EDTA (0.025%/0.01%, v/v, 50 µL).

The cells were re-suspended in RPMI 1640 medium containing 5% FBS, 1% L-glutamine and 1% penicillin/streptomycin (100 µL). Finally, the fluorescence of live cells (λ_{ex} = 488 nm, λ_{em} = 530 nm) in the suspensions was determined by using flow cytometry (Figure S62).

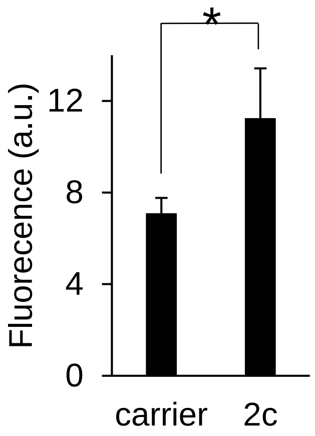

Figure **S62**. Fluorescence of DAF-FM diacetate-loaded A2780 cells treated either with **2c** or the carrier. *: p<0.05, Student's t test.

Effect of prodrug 2c on mitochondrial reactive oxygen species in representative cancer (A2780) and normal cells (SBLF9 fibroblasts) (Figure 6)

A2780 cells were seeded in of RPMI 1640 medium (500 µL) supplemented with 5% FBS, 1% L-glutamine, and 1% penicillin/streptomycin on a 35 mm imaging dish (µ- Dish 35 mm, high, ibidi GmbH, Germany) at a cell density of 80 cells/µL and allowed to attach to the surface of the dish for overnight. Next, N-acetylcysteine (NAC, 0.5 µL, 5 mM in water, final concentration: 5 µM) in fresh medium (0.5 mL, RPMI 1640 medium supplemented with 5% FBS, 1% L-glutamine, and 1% penicillin/streptomycin) was added to replace old medium and incubated for 24 h at 37 °C and 5% CO_2. Then prodrug (5 µL, 0.5 mM in DMSO, final concentration: 5 µM) or carrier (DMSO, 5 µL, 1%, v/v) were added and incubated for 2 h under same conditions. The cells were washed with DPBS (2 x 2 mL) and MitoSOX (5 µL, 0.5 mM in DMSO, final concentration: 5 µM) in Hanks' Balanced Salt solution (0.5 mL) was added, followed by incubating for 20 min 37 °C and 5% CO_2.

Afterwards, the cells were washed with DPBS and a fresh portion of RPMI 1640 medium containing 5% FBS, 1% L-glutamine and 1% penicillin/streptomycin (1 mL) was added. The fluorescence images were taken with a Zeiss Axio Vert.A1 fluorescence microscope (λ_{ex}: 538-562 nm; λ_{em}: 570-640 nm; Objective: 40x/1.30 Oil DIC).

For SBLF9 the same procedure was applied except that Ham's F-12K (Kaighn's)-Medium supplemented with 15 % FBS, 2 % non-essential amino acid mix, and 1 % penicillin/streptomycin was used for cell seeding and incubation with the prodrug or the carrier. A cell density of 40 cells/µL was used.

Determination of viability of primary cancer (CLL) and normal (MNC) cells in the presence of prodrugs

Clinical samples from chronic lymphocytic leukemia (CLL) patients (N=6, CLL cells) and healthy donors (N=6; peripheral blood mononuclear cells, MNCs) were collected at the University Hospital Heidelberg, Germany. Informed consent was obtained in accordance with the Declaration of Helsinki. Sample collection and analysis was approved by the ethics committee of the University of Heidelberg (S-254/2016). Blood was separated by a Ficoll gradient (GE Healthcarey), and cells were cryopreserved. Drug sensitivity screening was performed in 384-well format (Greiner Bio One) with 10 different prodrug concentrations (30, 10, 3.3, 1.1, 0.37, 0.123, 0.041, 0.014, 0.005 and 0.002 µM) and $4x10^4$ cells per well. Cells were cultured in RPMI-1640 (Invitrogen) supplemented with penicillin/streptomycin (Invitrogen), L-glutamine (Invitrogen) and 10% pooled and heat inactivated AB-type human serum (Sigma-Aldrich). Cell viability was assessed by quantification of ATP (CellTiter Glo®, Promega) 48 hours after drug application. Luminescence was measured on a Tecan Infinite F200 Microplate Reader. Viability was calculated as % of solvent (DMSO) treated control. Representative plots of cell viability versus prodrug concentration are provided in Figure 7.

Study of synergy of prodrugs with radiotherapy for SAS and FaDu cells

Optimization of concentration of prodrugs and controls / MTT assay

The MTT assay was also applied to assess the impact of various drugs (0.01-100 µM) on the viability of the two human head and neck squamous carcinoma cell (HNSCC) lines SAS and FaDu as well as retinal pigment epithelial ARPE-19 cells and human fibroblasts (HF). Cells were seeded into 96-well plates at concentrations of 4000 and 2000 cells per well, respectively, for the 24 h and 48 h treatment intervals, and were pre-cultured for 24 h before exposure to the drugs. All drug dilutions were prepared from 20 mM stock solutions in DMSO. The final DMSO concentration was set to 0.5% in all samples including control. After completion of the treatment, the wells were processed according to the routine MTT protocol as described above; the formazan absorbance was then measured on a Biotek Epoch Microplate Spectrophotometer at 590 nm and corrected for the background signal at 620 nm. N≥3 independent experiments were performed for each drug candidate, cell type and treatment interval. Cell survival was recorded as function of the drug concentration, and dose-response curves were fitted with the Boltzmann equation where appropriate. We calculated the half-maximal response (EC_{50}) as well as the EC_{20} (80% survival) according to the line curve (see Tables S6-S8). EC_{20}-values were used as a basis to select the compound-specific non-toxic drug concentrations for combination with radiotherapy. Figure S63 representatively documents the cell survival data for the 48 h exposure time.

Table **S6**. EC_{50} values (50% survival) in cancer SAS and FaDu cells determined via the MTTassay after 48 h of exposure to the prodrugs.

**Compound**	**Cytotoxicity Assay (MTT) – 50% cell survival (EC_{50} in µM)**			
	**SAS**		**FaDu**	
	**24h**	**48h**	**24h**	**48h**
**2c**	ND (>50)	ND (>50)	ND (>50)	ND (>50)
**5b**	18.7 ± 4.7	5.8 ± 0.8	26.3 ± 3.4	19.8 ± 0.3
**6**	ND (>50)	ND (>50)	ND (>50)	ND (>50)
**7**	1.7 ± 0.1	1.6 ± 0.3	1.5 ± 0.1	1.5 ± 0.1

[i] ND= not detectable.

Table **S7**. EC_{50} values (50% survival) in representative normal ARPE-19 and human fibroblasts (HF) cells determined via the MTTassay after 48 h of exposure to the prodrugs.

Compound	Cytotoxicity Assay (MTT) – 50% cell survival (EC_{50} in μM)			
	**ARPE-19**		**human fibroblasts (HF)**	
	**24h**	**48h**	**24h**	**48h**
**2c**	ND (>50)	ND (>50)	ND (>50)	ND (>50)
**5b**	40.4 ± 2.5	26.6 ± 8.3	35.5 ± 14.9	20.8 ± 4.6
**6**	ND (>50)	ND (>50)	ND (>50)	ND (>50)
**7**	2.0 ± 0.4	1.7 ± 0.2	1.7 ± 0.2	1.6 ± 0.3

[i] ND= not detectable.

Table **S8**. EC_{20} values (80% survival) in cancer SAS and FaDu cells determined via the MTTassay after 24 h and 48 h of exposure to the prodrugs.[i]

Compound	80% cell survival (EC_{20} in μM)			
	**SAS**		**FaDu**	
	**24h**	**48h**	**24h**	**48h**
**2c**	ND (>50)	10.4 ± 6.8	ND (>50)	18.0 ± 5.1
**5b**	7.0 ± 1.5	2.4 ± 1.3	11.5 ± 1.3	7.3 ± 1.5
**6**	ND (>50)	ND (>50)	ND (>50)	ND (>50)
**7**	1.2 ± 0.1	1.0 ± 0.5	0.8 ± 0.2	0.9 ± 0.1

[i] ND= not detectable.

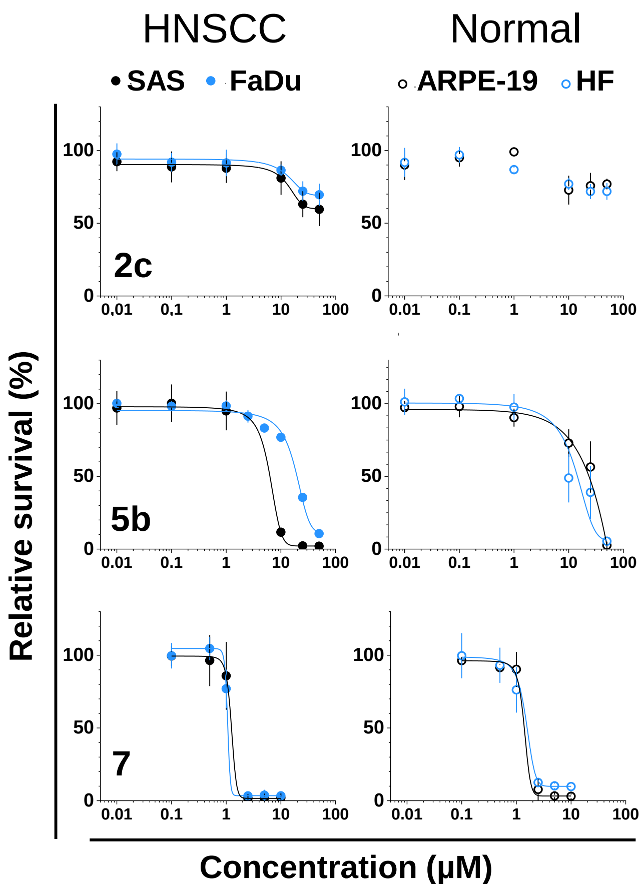

Figure **S63**. Representative experimental data showing relative survival as function of drug concentration in two HNSCC cell lines and two normal cell lines as determined via the MTT assays upon 48 h of exposure to various aminoferrocene-based prodrugs (mean values ± SD from N≥3 independent experiments are documented); where possible, dose-response curses were fitted with a Boltzmann equation.

<u>Optimization of concentration of prodrugs and controls / clonogenic assay</u>

The clonogenic survival assay is a classical approach to monitor radioresponse and drug- induced radiosensitization in cancer cells. We therefore initially determined whether the supposedly non-toxic drug concentrations defined for combinatorial treatment testing affect single cell colony formation capacity. For this purpose, exponentially growing SAS and FaDu cells were dissociated, single cell suspensions were prepared in standard DMEM and seeded in 6-well plates at

defined low density (150-300 cells in 1 ml per well). After 8 h of incubation to allow cell adherence, 50% of the supernatant was exchanged by media containing the respective prodrugs at 2x concentration and exposed for 48 h. Treatment was then terminated by medium removal, and cells were allowed to grow for a period of ≥5-6 cell divisions estimated from the cell line-specific doubling times of the respective untreated controls (~7 days for SAS and ~10 days for FaDu cells). Control cells were always handled in parallel to the treated cells and thus exposed to the corresponding DMSO concentration(s). After the defined growth intervals, cells were fixed, stained and colonies of >50 cells were manually counted at low magnification to calculate plating efficiencies as the number of counted colonies relative to the number of seeded cells. Drug effect on clonogenic survival was then estimated by normalizing the colony formation of control cells to 1 (Table S9).

The colony formation assay was also applied for monitoring radioresponse. Here, increasing numbers of single cells relative to the irradiation dose (0-10 Gy) were seeded into 6-well plates (150-2400 SAS cells/well and 300-4800 FaDu cells/well). Cells were treated with the drugs upon attachment as described. The total period of drug exposure was 48 h, and single dose irradiation was carried out 24 h after addition of the compounds. X-ray was performed at room temperature with a YxlonY.TU 320 system (Yxlon.international, 200 kV, 0.5 mm Cu filter) and a dose rate of ~1.3 Gy/min. Drug exposure was terminated by medium exchange and cultures were then incubated for the additional time period highlighted earlier. Plating efficiencies were assessed and normalized for each treatment arm to the respective unirradiated control. Data were reproduced (N=3 each with n=3 wells per experiment and treatment condition). The relative clonogenic cell survival (= survival fraction, SF) was then plotted as function of the irradiation dose, and clonogenic survival curves were fitted employing the linear quadratic model as described elsewhere.[S4] All results are summarized in Table S9.

Table **S9.** Effects of the prodrugs on the capacity of SAS and FaDu cells to form colonies; data are given relative to untreated control cells (= relative clonogenic survival) and are documented as means ± SD from N=3 independent experiments.

**Compound**	**Concentration (µM)**	**Relative clonogenic survival**	
		**SAS**	**FaDu**
**2c**	10	0.78 ± 0.13	0.86 ± 0.13
	25	0.70 ± 0.10	0.20 ± 0.08
**5b**	0.5	0.96 ± 0.06	0.71 ± 0.07
	1	0.76 ± 0.06	0.75 ± 0.11
	2.5	0.36 ± 0.15	0.36 ± 0.10
**6**	25	0.88 ± 0.10	0.99 ± 0.08
**7**	0.1	0.94 ± 0.12	0.97 ± 0.10

N=3; ∑n=9

References

[S1] J. Sangster, Octanol‐water partition coefficients of simple organic compounds, J. Phys. Chem. Ref. Data 18 (1989) 1111-1229.

[S2] LogP value of phenylboronic acid was obtained from RSC ChemSpider website.

[S3] A. Cossarizza *et al*, Simulataneous analysis of reactive oxygen species and reduced glutathione content in living cells using polychromatic flow cytometry, Nature protocols 4 (2009) 1790-1797.

[S4] C. N. Hinrichs, Kunz-Schughart *et al*, Mol. Cancer Ther. 17 (2018) 393-406.
